# Photoswitchable Bis(amidopyrroles): Modulating Anion Transport Activity Independent of Binding Affinity

**DOI:** 10.1021/acs.joc.3c01018

**Published:** 2023-07-13

**Authors:** David Villarón, Jasper E. Bos, Fabien Kohl, Stefan Mommer, Jorn de Jong, Sander J. Wezenberg

**Affiliations:** Leiden Institute of Chemistry, Leiden University, Einsteinweg 55, 2333 CC Leiden, The Netherlands

## Abstract

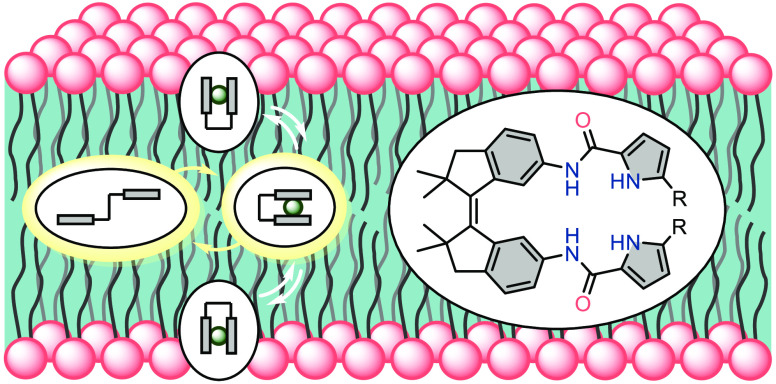

Toward photocontrol of anion transport across the bilayer membrane, stiff-stilbene, which has dimethyl substituents in the five-membered rings, is functionalized with amidopyrrole units. UV–vis and ^1^H NMR studies show high photostability and photoconversion yields. Where the photoaddressable (*E*)- and (*Z*)-isomers exhibit comparable binding affinities, as determined by ^1^H NMR titrations, fluorescence-based transport assays reveal significantly higher transport activity for the (*Z*)-isomers. Changing the binding affinity is thus not a necessity for modulating transport. Additionally, transport can be triggered *in situ* by light.

Driven by the important role of anions in many biological processes, a large number of artificial anion receptors have been developed.^1^ These receptors have found applications in analyte sensing,^2^ wastewater extraction,^3^ and transmembrane transport.^4^ With respect to the latter, defects in anion transport by proteins have been linked to serious illnesses (e.g., cystic fibrosis), and synthetic systems with transport capabilities therefore have therapeutic potential.^5^ Although a number of synthetic receptors have been shown to facilitate anion transport, they usually do not exhibit stimulus-controlled conformational changes, which are a hallmark of proteins. To endow them with stimulus-responsive properties remains a fundamental challenge,^6,7^ and current approaches are primarily based on the control of the binding affinity, which presumably translates into transport activity. The use of light to control transport activity is advantageous, as it can be applied with high spatiotemporal precision and does not produce chemical waste.^8^ Indeed, a significant amount of light-responsive anion receptors have been developed over the past decade,^9^ and a small number of them were shown to be capable of mediating transmembrane transport.^7^ The groups of Jeong^7a^ and Langton,^7b^ for example, demonstrated light-controlled chloride transport using azobenzene appended with (thio)urea and squaramide groups, respectively. Furthermore, in collaboration with the group of Gale, we recently described the photocontrol of membrane transport as well as the potential use of stiff-stilbene-derived bis(thio) urea receptors.^7f^ Nevertheless, crucial design parameters for light-controllable anion receptors still need to be identified, and furthermore exploration of other binding motifs as well as improvement of photoswitching properties are needed in order to get closer to practical (and biological) applications.

Among extensively studied pyrrole-containing receptors,^10^ amidopyrroles have been used successfully in anion binding and, in one case, also in transmembrane transport.^11^ We envisioned that functionalizing stiff-stilbene with amidopyrrole units in the 6,6′-positions would create a suitable anion binding pocket in the (*Z*)-isomer, whereas in the (*E*)-isomer the binding units would be far apart from each other, thus leading to inferior binding and transport behavior. Our group demonstrated previously that stiff-stilbene provides an excellent scaffold for designing photoswitchable receptors due to its high structural rigidity, large geometrical change upon isomerization, and very good thermal stability.^7f,12b^ In the present design, dimethyl substituents were incorporated into the five-membered rings to increase steric crowding in the vicinity of the central double bond, which was shown in two other cases to improve photostationary state (PSS) ratios as well as resistance to fatigue.^13^

Herein, we describe bis(amidopyrrole) receptors **1** and **2** (Scheme 1), where the electron-withdrawing CF_3_ groups were introduced into the latter compound to enhance NH proton acidity. Both receptors display robust bidirectional photoswitching, and while their (*E*)- and (*Z*)-isomers have virtually no difference in anion (i.e., acetate and chloride) binding affinity, they do display significantly different chloride transport activity. As a result, transmembrane transport can be activated *in situ* by light irradiation. Our results illustrate that affinity control is not a necessity for modulating transport activity, which is important to consider in future designs of photoresponsive transmembrane transporters. Such transporters could potentially be applied as light-controlled physiological tools or therapeutic agents.

**Scheme 1:**
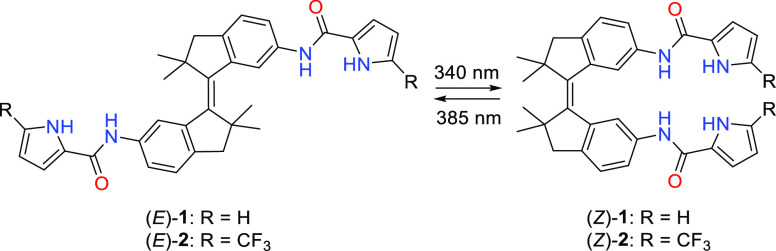
Photoisomerization of Bis(amidopyrroles) **1** and **2**

The synthesis of bis(amidopyrrole) receptors **1** and **2** is outlined in Scheme 2. The starting 6-bromo-2,2-dimethyl-1-indanone (**3**) was prepared according to a procedure described by the group of Diederich.^14^ McMurry homocoupling of this indanone yielded dibromo-substituted (*E*)-**4**. Subsequent Buchwald–Hartwig amination gave compound (*E*)-**5**, which was reacted with the respective pyrrole-carboxylic acid using HBTU to afford the bis(amidopyrrole) receptors (*E*)-**1** and (*E*)-**2**. Where pyrrole-2-carboxylic acid is commercially available, its trifluoromethylated derivative **7** was obtained by treatment of methyl 2-pyrrole-carboxylate with sodium triflinate in the presence of *tert*-butyl hydroperoxide, which was followed by cleavage of the methyl ester using TMSCl/NaI. The (*Z*)-isomers of the bis(amidopyrrole) receptors were generated from the corresponding (*E*)-isomers by 340 nm irradiation in a chloroform solution. The resulting *E*/*Z* mixture was separated by column chromatography (see the SI for synthetic details and characterization).

**Scheme 2:**
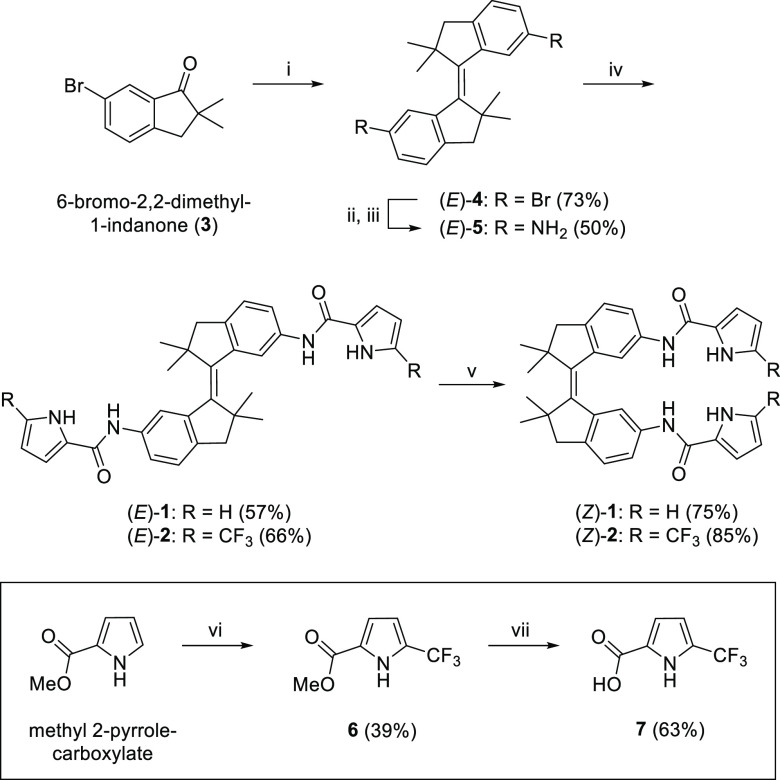
Synthesis of Bis(amidopyrrole) Receptors (i) Zn, TiCl_4_, THF, reflux; (ii) benzophenone imine, Pd(OAc)_2_, DPPF, NaO*t*Bu, toluene, 90 °C; (iii) 2 M aqueous HCl, THF; (iv) pyrrole-2-carboxylic acid or compound **7**, HBTU, DIPEA, CH_2_Cl_2_; (v) 340 nm light irradiation, CHCl_3_; (vi) CF_3_SO_2_Na, *t*BuOOH, CH_2_Cl_2_/H_2_O 7:3; (vii) NaI, TMSCl, MeCN, reflux.

The photoswitching behavior of **1** and **2** was first studied by UV–vis spectroscopy in a DMSO solution. Both (*E*)-isomers showed absorption maxima at around 290 and 350 nm (Figure 1A and B). Irradiation with 365 nm light led to a decrease in these maxima and a small increase in the longer wavelength absorption, indicating formation of the respective (*Z*)-isomers.^12b^ The spectra changed further by subsequent 340 nm irradiation, revealing higher conversion toward the (*Z*)-isomers with this wavelength. The opposite spectral changes were observed when 385 nm was then used, demonstrating reversibility of the isomerization process. In all cases, irradiation was halted when no further changes in absorption were observed, indicating that the photostationary states (PSS) had been reached. Importantly, clear isosbestic points were maintained during irradiation, illustrative of a unimolecular isomerization process (Figures S21–S24). Furthermore, alternation of 340 and 385 nm irradiation showed excellent fatigue resistance (Figure 1C and D).^15^

**Figure 1:**
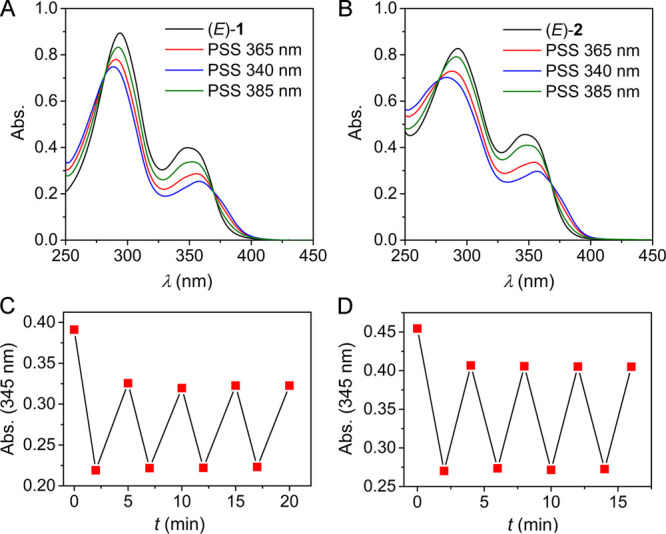
(A) UV–vis spectral changes of (*E*)-**1** upon sequential irradiation with 365 (10 s), 340 (140 s), and 385 nm (180 s) light and (B) UV–vis spectral changes of (*E*)-**2** upon sequential irradiation with 365 (10 s), 340 (120 s), and 385 nm (120 s) light (*c* = 2.0 × 10^–4^ M in degassed DMSO). The absorption change (at λ = 345 nm) upon multiple 340/385 nm irradiation cycles starting with (C) (*E*)-**1** and (D) (*E*)-**2** is also shown.

Next, ^1^H NMR studies were performed to determine the PSS ratios. Irradiation of the (*E*)-isomers in DMSO-*d*_6_ with 340 nm light led to the formation of new sets of ^1^H NMR signals, which were assigned to the respective (*Z*)-isomers (Figures S25 and S26). By subsequent irradiation with 385 nm light, the (*E*)-isomers were partially recovered. The PSS_340_ and the PSS_385_ (*E*/*Z*) ratios were determined as 19:81 and 82:18 for **1** and as 23:77 and 83:17 for **2**, respectively. Using the UV–vis absorbance data, PSS_365_ (*E/Z*) ratios of 40:60 for **1** and 45:55 for **2** were derived. Similar ratios have been reported for other stiff-stilbene derivatives containing dimethyl substituents in the five-membered rings.^9a,12b,13,16^

The possibility of the (*Z*)-isomer to form 1:1 complexes with either acetate or chloride was first assessed by DFT geometry optimizations (Figure 2 and Tables S1 and S2 for details). These anions were chosen because they have previously been found to give 1:1 binding with structurally related stiff-stilbene-based urea receptors, which additionally were shown to be capable of mediating transmembrane chloride transport.^7f,9g^ The energy-minimized structure of (*Z*)-**1**⊂AcO^–^ displayed amide and pyrrole N(H)···O hydrogen bond distances of 2.89 and 2.76 Å, respectively, and a central C_Ph_—C=C—C_Ph_ dihedral angle of ϕ = 20.6°. For the chloride-bound complex, hydrogen bond lengths were longer (i.e., N(H)···Cl^–^ distances of 3.38 and 3.27 Å for amide and pyrrole, respectively), while the dihedral angle was slightly smaller (ϕ = 18.8°). From these calculations, 1:1 binding of the (*Z*)-isomer with both anions thus seemed viable.

**Figure 2:**
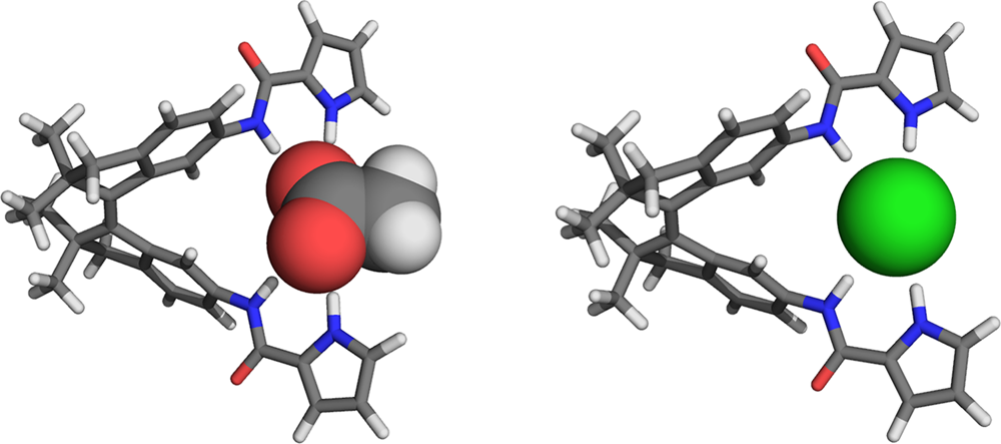
DFT-optimized molecular geometries of (*Z*)-**1**⊂AcO^–^ (left) and (*Z*)-**1**⊂Cl^–^ (right) at the B3LYP/6-31G++(d,p) level of theory using an IEF-PCM (DMSO) solvation model.

The binding strength of these anions was quantified by ^1^H NMR spectroscopic titrations in DMSO-*d*_6_/0.5%H_2_O. Addition of their NBu_4_^+^ salts to the bis(amidopyrrole) receptors resulted in downfield shifting of the signals belonging to the amide and pyrrole NH protons, illustrating involvement in hydrogen bonding (Figures S27–S34). Furthermore, small chemical shift changes of the aromatic signals were noted. An exception was the titration of (*E*)-**2** and (*Z*)-**2** with AcO^–^, which resulted in the disappearance of the pyrrole NH signal, most likely as a sign of deprotonation.^10a^ In contrast to what was expected based on the DFT modeling, modified Job’s plot analysis for the titration of AcO^–^ to (*Z*)-**1** hinted at a 1:2 binding stoichiometry. The same stoichiometry was deduced for (*E*)-**1** (Figures S36–S37). The titration data were therefore fitted to a 1:2 binding model (using HypNMR)^17^ by treating the two amidopyrrole binding sites as equal (cooperativity factor α = 1, see Figure S35 and Figures S38–S43 in the SI for details). Interestingly, the obtained binding constants for both isomers were comparable [*K*_11_ = 98 M^–1^ and 102 M^–1^ for (*E*)-**1** and (*Z*)-**1**, respectively].

Fitting the data for Cl^–^ binding using a 1:2 binding model also gave virtually the same association constants for both isomers and, moreover, revealed very weak binding [*K*_11_ ∼ 3–4 M^–1^ for (*E*)-**1** and (*Z*)-**1**]. Nevertheless, the binding strength was slightly enhanced by the presence of electron-withdrawing CF groups, as can be expected on the basis of increased NH proton acidities [*K*_11_ ∼ 6 M^–1^ for (*E*)-**2** and (*Z*)-**2**]. For the (*E*)-isomer, 1:2 binding was anticipated, since the binding motifs are too far apart from each other to bind a single anion simultaneously. It appears that for the (*Z*)-isomer such simultaneous binding is also disfavored, which contrasts our previous findings with stiff-stilbene-based bis(urea) receptors. The difference may be ascribed tentatively to an enlarged dihedral angle as compared to that of regular stiff-stilbene,^9g^ which is caused by the steric crowding of the dimethyl-substituents attached to the five-membered rings.

In spite of the weak binding and comparable affinities for the photoaddressable isomers, the capability of receptors **1** and **2** to mediate chloride transport was validated in an 8-hydroxypyrene-1,3,6-trisulfonate (HPTS) assay (see the SI for details).^18^ Initially, the compounds were added as DMSO solutions to POPC vesicles (200 nm mean diameter), which were loaded with the pH-sensitive HPTS fluorescent dye in an aqueous NaCl solution buffered to pH 7.0, whereafter a NaOH base pulse was applied to generate a pH gradient. In the presence of sufficient amounts of receptor, the pH gradient dissipated, which occurs by receptor-mediated Cl^–^/H^+^ symport or Cl^–^/OH^–^ antiport (as indicated by the change in HPTS emission; see Figures S44–S51). Concentration-dependent runs and fitting of the data to the Hill equation revealed the half-maximal effective concentration values (EC_50_; see Table 1). From these studies, both receptors appeared to have a moderate activity. However, for the CF_3_-functionalized receptor **2**, 100% chloride efflux was never reached, even at the highest loading (10 mol %), which could indicate issues with membrane solubility or deliverability.^18b^

**Table 1:** Chloride Transport Activity (EC_50_)^a^ of Compounds **1** and **2**

	postadded		preincorporated	
compound	EC_50(*E*)_ (mol %)	EC_50(*Z*)_ (mol %)	EC_50(*E*)_ (mol %)	EC_50(*Z*)_ (mol %)
**1**	3.11	1.71	3.20	1.69
**2**	1.70	0.53	0.26	0.062

a Defined as the transporter-to-lipid molar ratio (mol %) needed to reach 50% of the maximum possible chloride efflux.

The HPTS assay was therefore repeated with the compounds preincorporated into the POPC lipid bilayer. For receptor **1**, the activities were virtually unaltered with respect to postaddition, and it was confirmed that the (*Z*)-isomer is more active than the (*E*)-isomer (1.9-fold). For **2** instead, now maximum efflux was reached (at 1 mol % loading) and, gratifyingly, the (*Z*)-isomer turned out to be an active transporter, whereas the (*E*)-isomer displayed a 4.1-fold lower activity. Interestingly, the EC_50_ value of (*Z*)-**2** is in the same range as that determined previously for our stiff-stilbene bis(thio)ureas,^7f^ as well as for azobenzene-based bis(squaramides),^7b,7e^ while its chloride binding affinity is lower. Furthermore, in contrast to previously reported photoswitchable transporters, the binding affinities of both isomers are similar in this case, and still the (*Z*)-isomer is significantly more active than the (*E*)-isomer (Figure 3A). This higher activity is proposed to originate from other factors contributing to transport efficiency such as a better mobility and partioning in the membrane,^7,19^ in addition to an improved anion encapsulation ability.^20^

**Figure 3:**
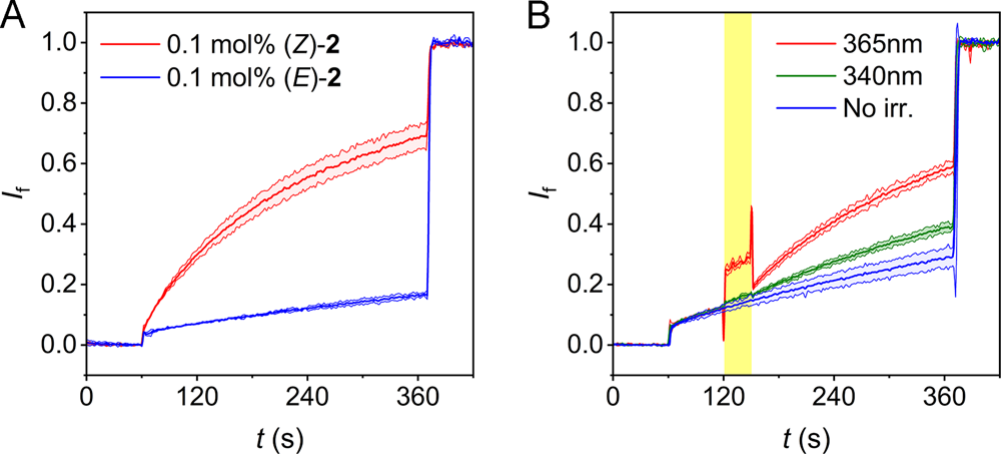
Plot of the fractional fluorescence intensity (*I*_f_) as a measure of chloride transport (A) facilitated by (*Z*)-**2** and (*E*)-**2** (0.1 mol %, preincorporated) and (B) starting with (*E*)-**2** (0.1 mol %) and activation by *in situ* irradiation using 340 or 365 nm light for 30 s.^21^

In the same HPTS assay, control over transport activity by *in situ* irradiation was demonstrated. Compound (*E*)-**2** was preincorporated into POPC vesicles and, 60 s after the base pulse was applied, irradiation at 340 or 365 nm for 30 s led to the enhancement of transport (Figure 3B), which is explained by isomerization to the more active (*Z*)-**2**. The largest effect was observed with the longer wavelength, which is likely explained by higher conversion to the active isomer within the short irradiation time. It should be noted here that although the PSS ratio is lower upon irradiation with 365 nm light than that with 340 nm light, the PSS is reached much faster with the former wavelength (Figure 1B).^22^

Finally, chloride transport facilitated by the more active (*Z*)-isomers was additionally studied in a cationophore-coupled ion-selective electrode (ISE) assay using POPC vesicles (200 nm mean diameter) with an internal buffered KCl solution and suspended in a buffered KGlu solution (pH 7.2, see the SI for details).^18a^ The chloride gradient was dissipated by preincorporated (*Z*)-isomers after the addition of either monensin or valinomycin, showing that they are capable of both electroneutral and electrogenic transport (Figures S55 and S56). Only slightly faster efflux was observed when coupled to the latter cationophore, revealing that there is no significant Cl^–^ uniport selectivity.

In summary, two bis(amidopyrrole)-functionalized stiff-stilbene derivatives, having dimethyl-substituted five-membered rings, were synthesized. These derivatives could be effectively switched between (*E*)- and (*Z*)-isomers using 340/385 nm light, showing improved PSS ratios and fatigue resistance in comparison to regular stiff-stilbene derivatives. Although similar binding affinities were determined for the photoaddressable isomers, they exhibited distinct chloride transport activities. Consequently, transport could be activated *in situ* by light. In this system, the change in transport activity upon isomerization is clearly governed by factors other than binding affinity, which is important to take into account in the future design of light-responsive transporters. Hence, our results open a new perspective on the development of photoactivatable transporters, which could potentially be applied as physiological tools or therapeutic agents to study and treat diseases by facilitating the passage of anions across the lipid bilayer membrane.

## Experimental Section

### General Methods and Materials

THF, MeCN, and CH_2_Cl_2_ were dried using a Pure Solve 400 solvent purification system from Innovative Technology. Dry DMSO and toluene were purchased from Acros Organics, and DMSO-*d*_6_, MeCN-*d*_3_ and CDCl_3_ were purchased from Eurisotop. DMSO-*d*_6_ was stored under N_2_ over molecular sieves (4 Å). The degassing of the solvents was carried out by purging with N_2_ for 30 min unless noted otherwise. 6-Bromo-2,2-dimethyl-1-indanone (**3**) was prepared using a procedure reported in the literature.^14^ All other chemicals were commercial products and were used without further purification. Column chromatography was performed using silica gel (SiO_2_) purchased from Screening Devices BV (Pore diameter 55–70 Å, surface area 500 m^2^ g^–1^). Thin-layer chromatography (TLC) was carried out on aluminum sheets coated with silica 60 F254 and neutral aluminum oxide obtained from Merck. Compounds were visualized with UV light (254 nm) or by staining with potassium permanganate. ^1^H, ^13^C, and ^19^F NMR spectra were recorded on Bruker AV 400 and Bruker 500 Ultra Shield instruments at 298 K unless indicated otherwise. Chemical shifts (δ) are denoted in parts per million (ppm) relative to residual protiated solvent (DMSO-*d*_6_, δ = 2.50 and 39.52 ppm for ^1^H detection and ^13^C detection, respectively; CDCl_3_, δ = 7.26 and 77.16 ppm; for ^1^H detection and ^13^C detection, respectively). The splitting pattern of peaks is designated as follows: s (singlet), d (doublet), t (triplet), q (quartet), p (quintet), h (septet), m (multiplet), and br (broad). High-resolution mass spectrometry (ESI-MS) was performed on a Thermo Scientific Q Exactive HF spectrometer with ESI ionization. IR spectra were recorded on a PerkinElmer Spectrum Two FT-IR spectrometer. The wavenumber (ν) is in units of reciprocal centimeters (cm^–1^), and the intensity is designated as follows: s (strong), m (medium), w (weak), very w (very weak), br (broad), and sh (shoulder). Melting points were determined with a Büchi M560 apparatus. UV–vis spectra were recorded on an Agilent Cary 8454 spectrometer using 1 cm or 1 mm quartz cuvettes. Fluorescence was measured on a JASCO FP-8500 spectrofluorimeter using 1 cm PS cuvettes. Irradiation of samples was carried out using Thorlabs model M340F3 (0.85 mW, λ_em_ = 340 ± 6 nm), M365F1 (4.1 mW, λ_em_ = 365 ± 4 nm), and M385F1 (9.0 mW, λ_em_ = 385 ± 5 nm) instruments positioned at a distance of 1 cm to the sample unless noted otherwise.

### (*E*)-6,6′-Dibromo-2,2,2′,2′-tetramethyl-2,2′,3,3′-tetrahydro-1,1′-biindenylidene [(*E*)-**4**]

First, TiCl_4_ (5.50 mL, 50.1 mmol) was slowly added to a vigorously stirred suspension of Zn (6.56 g, 100 mmol) in dry THF (60 mL) under a N_2_ atmosphere. The solution was stirred at reflux for 2 h using an oil bath and then cooled to rt. Subsequently, compound **3** (5.99 g, 25.1 mmol) was added to the black suspension, and the mixture was stirred at reflux for 16 h using and oil bath, cooled to rt, treated with saturated aqueous NH_4_Cl solution (60 mL), and extracted with CHCl_3_ (3 × 150 mL). The combined organic layers were dried over Na_2_SO_4_ and concentrated. Purification by column chromatography (SiO_2_, pentane) afforded (*E*)-**4** (4.06 g, 73%) as a white solid; *R*_f_ = 0.63 (SiO_2_, pentane); mp 162.5–163.7 °C; ^1^H NMR (400 MHz, CDCl_3_) δ = 7.57 (d, *J* = 1.9 Hz, 2H), 7.29 (dd, *J* = 7.9, 1.9 Hz, 2H), 7.06 (d, *J* = 7.9 Hz, 2H), 2.72 (s, 4H), 1.31 (s, 12H) ppm; ^13^C{^1^H} NMR (100 MHz, CDCl_3_) δ = 146.2, 144.7, 144.2, 130.8, 130.2, 125.9, 118.7, 51.5, 51.3, 27.8 ppm; IR (ATR) ν = 2971 (w, sh), 2955 (m), 2926 (w), 2901 (w), 1591 (m), 1564 (w), 1463 (s), 1402 (m), 1382 (m), 1369 (m), 1320 (m), 1274 (m), 1252 (w), 1164 (m), 1095 (w), 1067 (s), 889 (m, sh), 882 (s), 652 (m), 627 (m), 609 (m) cm^–1^.

### (*E*)-2,2,2′,2′-Tetramethyl-2,2′,3,3′-tetrahydro-[1,1′- biindenylidene]-6,6′-diamine [(*E*)-**5**]

Compound (*E*)-**4** (2.03 g, 4.55 mmol), palladium(II) acetate (0.16 g, 0.73 mmol), DPPF (0.25 g, 0.45 mmol), and sodium *tert*-butoxide (0.88 g, 9.1 mmol) were placed in a Schlenck tube and brought under N_2_ via three vacuum/N_2_ cycles. Then, to the reaction mixture was added dry and degassed toluene (25 mL), followed by benzophenone imine (1.91 mL, 11.4 mmol), and the mixture was stirred at 90 °C for 21 h using an oil bath, cooled to rt, and diluted with water (30 mL). The aqueous layer was extracted with CHCl_3_ (3 × 125 mL). The combined organic layers were dried over Na_2_SO_4_ and concentrated. Purification by column chromatography (SiO_2_, 0.1% NEt_3_ in CH_2_Cl_2_) afforded the imine intermediate as a yellow oil. Subsequently, this imine intermediate was redissolved in THF (100 mL), and to the mixture was added a 2 M aqueous HCl solution (50 mL). The mixture was stirred for 1 h at rt, diluted with H_2_O (100 mL), and extracted with Et_2_O (3 × 60 mL) to remove excess of benzophenone imine formed during the reaction. The resulting water layer was treated with K_2_CO_3_ (pH ∼ 10) and extracted with EtOAc (3 × 60 mL). The combined organic layers were dried over Na_2_SO_4_ and concentrated to afford (*E*)-**5** (0.72 g, 50%) as a white solid. *R*_f_ = 0.59 (SiO_2_, CH_2_Cl_2_/EtOAc 1:1); mp 182.2–183.3 °C; ^1^H NMR (400 MHz, DMSO-*d*_6_) δ = 6.84 (d, *J* = 7.9 Hz, 2H), 6.72 (d, *J* = 2.1 Hz, 2H), 6.39 (dd, *J* = 7.9, 2.0 Hz, 2H), 4.84 (s, 4H), 2.58–2.53 (m, 4H), 1.25 (s, 12H) ppm; ^13^C{^1^H} NMR (100 MHz, DMSO-*d*_6_) δ = 145.9, 145.4, 143.0, 132.3, 124.0, 113.6, 113.5, 51.0, 50.2, 27.6 ppm; IR (ATR) ν = 3423 (w), 3330 (w), 2975 (w, sh), 2953 (m, sh), 2943 (m), 2921 (m), 2900 (m), 2856 (w, sh), 2844 (w, sh), 1695 (very w) 1618 (m, sh), 1607 (m), 1583 (m), 1486 (s), 1466 (w, sh), 1454 (m), 1379 (w), 1361 (m), 1328 (m), 1252 (m), 1189 (m), 1066 (br, m), cm^–1^; HRMS (ESI) *m/z* 319.2165 ([M + H]^+^, calcd for C_22_H_27_N_2_^+^ 319.2168).

### (*E*)-*N*,*N*′-(2,2,2′,2′-Tetramethyl-2,2′,3,3′-tetrahydro-[1,1′-biindenylidene]-6,6′-diyl) Bis(1*H* -pyrrole-2-carboxamide) [(*E*)-**1**]

Compound (*E*)-**5** (100 mg, 0.314 mmol) and 1*H*-pyrrole-2-carboxylic acid (84 mg, 0.75 mmol) were placed in an oven-dried two-neck flask under a N_2_ atmosphere. Then, dry and degassed CH_2_Cl_2_ (5 mL) was added, followed by HBTU (272 mg, 0.716 mmol) and DIPEA (263 μL, 1.51 mmol). The mixture was stirred at rt for two days, diluted with H_2_O (10 mL) and extracted with CH_2_Cl_2_ (5 × 15 mL). The combined organic phase was washed with 1M aqueous HCl solution (10 mL) and brine (10 mL), dried over Na_2_SO_4_, and concentrated. Purification by column chromatography (SiO_2_, pentane/EtOAc 70:30) afforded (*E*)-**1** (90 mg, 57%) as a white solid; *R*_f_ = 0.30 (SiO_2_, CH_2_Cl_2_/MeOH 98:2); mp 285.8–286.7 °C; ^1^H NMR (400 MHz, DMSO-*d*_6_) δ = 11.74 (s, 2H), 9.71 (s, 2H), 8.24 (d, *J* = 1.9 Hz, 2H), 7.31 (dd, *J* = 8.2, 1.9 Hz, 2H), 7.17 (d, *J* = 8.0 Hz, 2H), 7.07–7.03 (m, 2H), 6.96–6.91 (m, 2H), 6.18–6.13 (m, 2H) 2.72 (s, 4H), 1.35 (s, 12H) ppm; ^13^C{^1^H} NMR (100 MHz, DMSO-*d*_6_) δ = 159.3, 149.9, 142.5, 139.5, 136.6, 126.2, 124.0, 122,4, 119.4, 119.2, 111.2, 108.9, 51.2, 50.8, 27.4 ppm; IR (ATR) ν = 3297 (w), 2957 (very w), 1648 (m), 1626 (m), 1614 (w, sh), 1587 (m), 1572 (w, sh), 1549 (m), 1524 (s), 1486 (m), 1444 (m), 1420 (m), 1345 (m), 1315 (m), 1260 (m), 1214 (w), 1197 (m), 1122 (m), 1045 (w) cm^–1^; HRMS (ESI) *m/z* 505.2596 ([M + H]^+^, calcd for C_32_H_33_N_4_O_2_^+^ 505.2598).

### (*Z*)-*N*,*N*′-(2,2,2′,2′-Tetramethyl-2,2′,3,3′-tetrahydro-[1,1′-biindenylidene]-6,6′-diyl) Bis(1*H*-pyrrole-2-carboxamide) [(*Z*)-**1**]

Compound (*E*)-**1** (16 mg, 0.032 mmol) was placed in an open round-bottom flask under a constant N_2_ flow and dissolved in degassed CHCl_3_ (38 mL). The solution was irradiated with a Thorlab model M340F3 LED (0.85 mW) through the opening of the round-bottom flask while stirring for 3 h at rt and keeping the volume constant by adding more CHCl_3_. Purification by column chromatography (SiO_2_, CH_2_Cl_2_/MeOH 98:2) afforded (*Z*)-**1** (12 mg, 75%) as a yellow solid. *R*_f_ = 0.41 (SiO_2_, CH_2_Cl_2_/MeOH 98:2); mp 320.1–320.9 °C; ^1^H NMR (400 MHz, DMSO-*d*_6_) δ = 11.46 (s, 2H), 9.43 (s, 2H), 7.81 (d, *J* = 2.0 Hz, 2H), 7.45 (dd, *J* = 8.0, 2.0 Hz, 2H), 7.16 (d, *J* = 8.0 Hz, 2H), 6.90–6.82 (m, 4H), 6.07–6.02 (m, 2H), 3.08–3.01 (m, 2H), 2.56–252 (m, 2H), 1.62 (s, 6H), 1.16 (6H) ppm; ^13^C{^1^H} NMR (100 MHz, DMSO-*d*_6_) δ = 158.8, 144.5, 143.0, 139.7, 136.2, 126.1, 124.4, 122.1, 120.5, 118.6, 111.1, 108.8, 51.4, 50.0, 28.7, 26.2 ppm; IR (ATR) ν = 3424 (very w), 3243 (br, m), 2988 (w, sh), 2923 (m), 1639 (w, sh), 1616 (m), 1594 (m), 1569 (m), 1551 (m), 1514 (br. s), 1440 (s), 1416 (m, sh), 1403 (m), 1333 (br. s), 1291 (m, sh), 1269 (m), 1229 (m), 1170 (m), 1143 (m), 1107 (m), 1092 (m), 1082 (m), 1046 (m), 1031 (m) cm^–1^; HRMS (ESI) *m/z* 505.2596 ([M + H]^+^, calcd for C_32_H_33_N_4_O_2_^+^ 505.2598).

### Methyl 5-(Trifluoromethyl)-1*H*-pyrrole-2-carboxylate (**6**)

Methyl 2-pyrrolecarboxylate (1.0 g, 8.0 mmol) and sodium triflinate (4.99 g, 32.0 mmol) were dissolved in 10 mL of CH_2_Cl_2_/H_2_O (7:3 v/v). Subsequently, a *tert*-butyl hydroperoxide solution (70 wt % in H_2_O, 8.81 mL, 91.4 mmol) was added dropwise at rt. The reaction mixture was stirred at rt for 24 h, treated with a saturated aqueous Na_2_SO_3_ solution (50 mL) and extracted with CH_2_Cl_2_ (3 × 100 mL). The combined organic layers were washed with brine (50 mL), dried over Na_2_SO_4_ and concentrated. Purification by column chromatography (SiO_2_, petroleum ether/EtOAc 90:10) afforded **6** (0.60g, 39%) as a white solid; *R*_f_ = 0.30 (SiO_2_, petroleum ether/EtOAc 90:10); mp 87.6–89.0 °C; ^1^H NMR (400 MHz, CDCl_3_) δ = 10.18 (s, 1H), 6.91–6.83 (m, 1H), 6.63–6.55 (m, 1H), 3.91 (s, 3H) ppm; ^13^C{^1^H} NMR (100 MHz, CDCl_3_) δ = 161.5, 125.1, 121.9, 115.1, 111.0, 52.3; ^19^F NMR (500 MHz, CDCl_3_) δ = −60.15 ppm; IR (ATR) ν = 3262 (m), 2965 (very w) 2916 (w), 2849 (w), 1699 (s), 1578 (w), 1461 (w), 1437 (m), 1279 (s), 1259 (m, sh), 1204 (m), 1155 (s), 1117 (w, sh), 1104 (s), 1047 (m) cm^–1^. HRMS (ESI) *m/z* 194.0422 ([M + H]^+^, calcd for C_7_H_7_F_3_NO_2_^+^ 194.0423).

### 5-(Trifluoromethyl)-1*H*-pyrrole-2-carboxylic acid (**7**)

Compound **6** (0.50 g, 2.6 mmol) and NaI (0.97 g, 6.5 mmol) were placed in an oven-dried three-neck flask and brought under a N_2_ atmosphere. Then, dry and degassed MeCN (30 mL) was added, followed by TMSCl (0.82 mL, 6.5 mmol). The mixture was stirred at reflux for 41 h using an oil bath, after which the solvent was evaporated. The resulting brown solid was triturated with EtOAc (150 mL) and filtered. The filtrate was washed with brine (3 × 30 mL), dried over Na_2_SO_4_, and concentrated. Purification by column chromatography (SiO_2_, petroleum ether/EtOAc/AcOH 79:20:1) afforded a yellow oil, which was dissolved in H_2_O (10 mL). Then, to the mixture was added saturated aqueous Na_2_CO_3_ solution (30 mL) (pH ∼ 10), and organic impurities were extracted with CH_2_Cl_2_ (3 × 50 mL). The aqueous layer was then treated with 6 M aqueous HCl solution (pH ∼ 1) and extracted with EtOAc (3 × 100 mL), and the combined organic layers were dried over Na_2_SO_4_ and concentrated to afford **7** (294 mg, 63%) as a white solid; *R*_f_ = 0.30 (SiO_2_, petroleum ether/EtOAc/AcOH 70:29:1); mp 106.5–108.3 °C; ^1^H NMR (400 MHz, CDCl_3_) δ = 10.32 (s, 1H), 9.65 (s, 1H), 7.06–6.99 (m, 1H), 6.67–6.61 (m, 1H) ppm; ^13^C{^1^H} NMR (100 MHz, CDCl_3_) δ = 165.4, 124.1, 121.6, 118.9, 117.3, 111.5; ^19^F NMR (470 MHz, CDCl_3_) δ = −60.32 ppm; IR (ATR) ν = 3196 (br. m), 1675 (s), 1645 (m, sh), 1571 (m), 1527 (w), 1508 (w), 1459 (w), 1432 (very w, sh), 1404 (m), 1327 (m), 1281 (w, sh), 1264 (s), 1257 (s), 1233 (s), 1200 (w, sh), 1169 (s), 1112 (s), 1103 (w, sh), 1043 (s) cm^–1^; HRMS (ESI) *m/z* 178.0115 ([M – H]^−^, calcd for C_6_H_3_F_3_NO_2_^–^ 178.0121).

### (*E*)-*N*,*N*′-(2,2,2′,2′-Tetramethyl-2,2′,3,3′-tetrahydro-[1,1′-biindenylidene]-6,6′-diyl) Bis(5-(trifluoromethyl)-1*H* -pyrrole-2-carboxamide) [(*E*)-**2**]

Compound (*E*)-**4** (100 mg, 0.314 mmol) and acid **6** (135 mg, 0.754 mmol) were placed in an oven-dried two-neck flask and brought under a N_2_ atmosphere. Then, dry and degassed CH_2_Cl_2_ (5 mL) was added, followed by HBTU (272 mg, 0.717 mmol) and DIPEA (263 μL, 1.51 mmol). The resulting mixture was stirred at rt for three days, diluted with H_2_O (10 mL), and extracted with CH_2_Cl_2_ (3 × 30 mL). The organic phase was washed with a 1 M aqueous HCl solution (10 mL) and H_2_O (10 mL). The combined organic layers were dried over Na_2_SO_4_ and concentrated. Purification by column chromatography (SiO_2_, pentane/EtOAc 90:10) afforded (*E*)-**2** as a white solid (133 mg, 66%); *R*_f_ = 0.40 (SiO_2_, pentane/*i*PrOH 95:5); mp 237.8–238.7 °C; ^1^H NMR (400 MHz, DMSO-*d*_6_) δ = 12.99 (s, 2H), 10.02 (s, 2H), 8.23 (d, *J* = 1.6 Hz, 2H), 7.32 (dd, *J* = 8.1, 1.9 Hz, 2H), 7.21 (d, *J* = 8.1, Hz, 2H), 7.12–7.08 (m, 2H), 6.71–6.67 (m, 2H), 2.74 (s, 4H), 1.36 (s, 12H) ppm; ^13^C{^1^H} NMR (100 MHz, DMSO-*d*_6_) δ = 158.4, 145.9, 142.5, 140.1, 136.0, 130.1, 124.1, 122.6, 122.2 119.6, 119.5, 110.9, 110.3, 51.1, 50.7, 27.3 ppm; ^19^F NMR (470 MHz, CDCl_3_) δ = −57.75 ppm; IR (ATR) ν = 3319 (w), 3154 (very w), 2925 (w), 1604 (s), 1583 (w, sh), 1545 (s), 1484 (m), 1414 (m), 1366 (m, sh), 1350 (m), 1325 (w, sh), 1311 (s), 1261 (s), 1172 (s), 1144 (m), 1116 (s), 1105 (s), 1039 (m) cm^–1^; HRMS (ESI) *m/z* 641.2341 ([M + H]^+^, calcd for C_34_H_31_F_6_N_4_O_2_^+^ 641.2345).

### (*Z*)-*N*,*N*′-(2,2,2′,2′-Tetramethyl-2,2′,3,3′-tetrahydro-[1,1′-biindenylidene]-6,6′-diyl) Bis(5-(trifluoromethyl)-1*H* -pyrrole-2-carboxamide) [(***Z***)-2]

Compound (*E*)-**2** (20 mg, 0.031 mmol) was dissolved in degassed CHCl_3_ (38 mL) in an open round-bottom flask under constant N_2_. The solution was irradiated with a Thorlab model M340F3 LED (0.85 mW) through the opening of the round-bottom flask while stirring for 3 h at rt and keeping the volume constant by adding more CHCl_3_. Purification by column chromatography (SiO_2_, pentane/*i*PrOH 95:5) afforded (*Z*)-**2** (17 mg, 85%) as a yellow solid; *R*_f_ = 0.57 (SiO_2_, pentane/*i*PrOH 95:5); mp 232.0–233.4 °C; ^1^H NMR (400 MHz, DMSO-*d*_6_) δ = 12.74 (s, 2H), 9.72 (s, 2H), 7.88 (d, *J* = 1.6 Hz, 2H), 7.47 (dd, *J* = 8.1, 1.8 Hz, 2H), 7.19 (d, *J* = 8.1 Hz, 2H), 6.94–6.87 (m, 2H), 6.60–6.54 (m, 2H), 3.10–3.0 (m, 2H), 2.57–2.52 (m, 2H), 1.62 (s, 6H), 1.16 (s, 6H) ppm; ^13^C{^1^H} NMR (125 MHz, DMSO-*d*_6_) δ = 157.9, 144.6, 143.1, 140.2, 135.8, 130.0, 124.6, 122.3, 121.9, 120.4, 119.6, 118.4, 110.8, 110.2, 51.5, 50.1, 28.6, 26.2 ppm; ^19^F NMR (470 MHz, CDCl_3_) δ = −57.76 ppm; IR (ATR) ν = 3200 (br. w), 2926 (w), 1601 (s), 1544 (s), 1485 (m), 1404 (m), 1311 (s), 1258 (s), 1170 (s), 1119 (s), 1037 (m) cm^–1^; HRMS (ESI) *m/z* 641.2343 ([M + H]^+^, calcd for C_34_H_31_F_6_N_4_O_2_^+^: 641.2345).

## Data Availability

The data underlying this study are available in the published article and its Supporting Information.

## Supporting Information Available

Synthetic methods and characterization of new compounds, ^1^H NMR and UV–vis photoisomerization studies, ^1^H NMR spectroscopic titrations and data fitting, DFT calculations, and anion transport studies (PDF)
