# Supplementary material for: Photoswitchable Bis(amidopyrroles): Modulating Anion Transport Activity Independent of Binding Affinity
Source: J Org Chem. 2023 Jul 13;88(15):11328–34. doi: 10.1021/acs.joc.3c01018 (PMC10407928; doi:10.1021/acs.joc.3c01018)
Supplement: Supplementary file 1 — jo3c01018_si_001.pdf [file jo3c01018_si_001.pdf]

SUPPORTING INFORMATION

Photoswitchable Bis-amidopyrroles: Modulating Anion Transport Activity Independent of Binding Affinity

David Villarón,† Jasper E. Bos,† Fabien Kohl, Stefan Mommer,
Jorn de Jong, and Sander J. Wezenberg*

Leiden Institute of Chemistry, Leiden University,
Einsteinweg 55, 2333 CC Leiden, The Netherlands

† These authors contributed equally to this paper

Email: s.j.wezenberg@lic.leidenuniv.nl

Table of contents

^{1}H, ^{13}C and ^{19}F NMR Spectra of new compounds S2

UV-vis photoisomerization studies S22

^{1}H NMR photoisomerization studies S24

^{1}H NMR titration experiments S26

^{1}H NMR titration data fitting S34

Transmembrane transport experiments S39

Geometry optimization by DFT S50

References S54

^{1}H, ^{13}C and ^{19}F NMR Spectra of new compounds

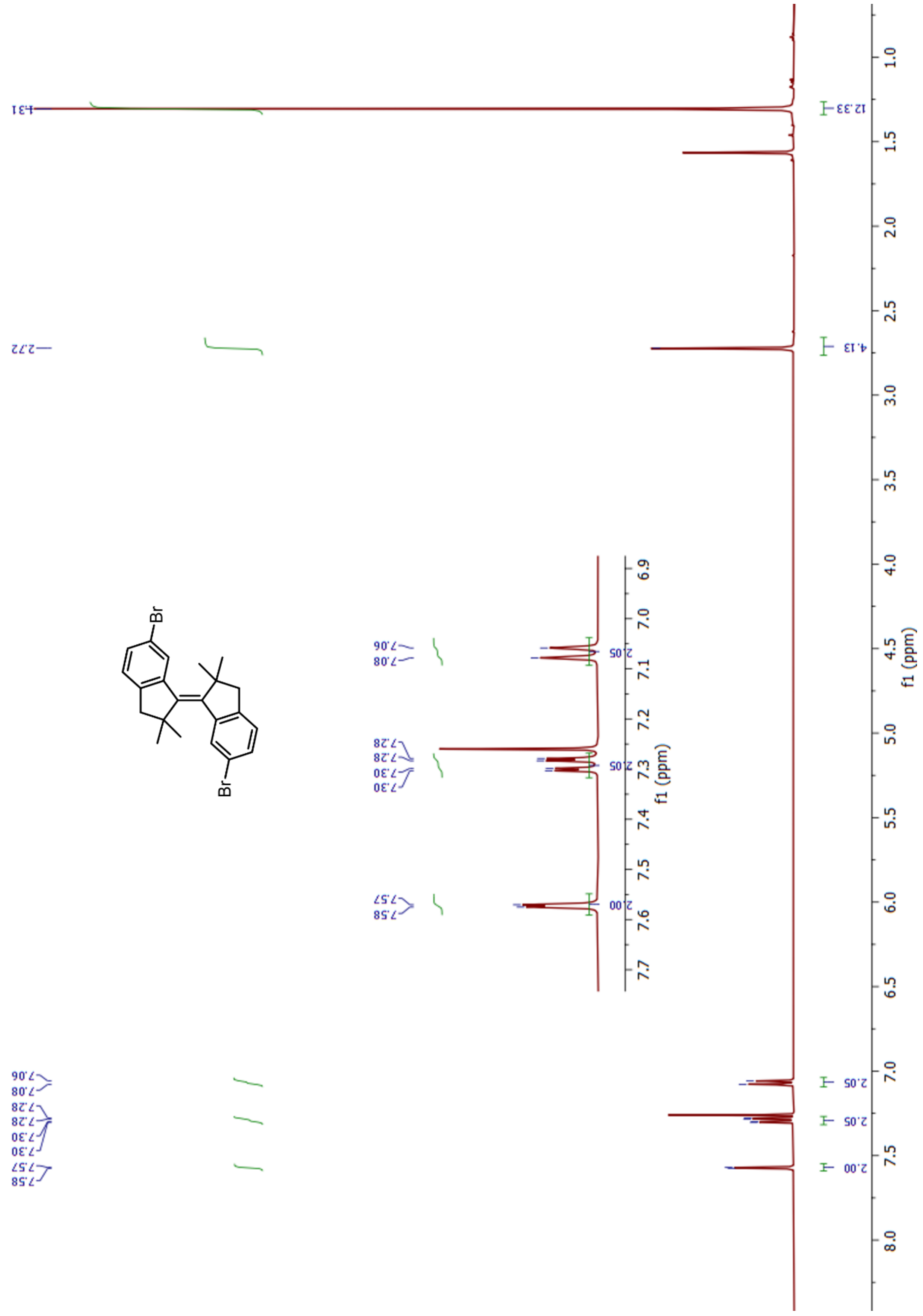

Figure S1. 400 MHz ^{1}H NMR spectrum of (*E*)-**4** measured at 298 K in $CDCl_3$.

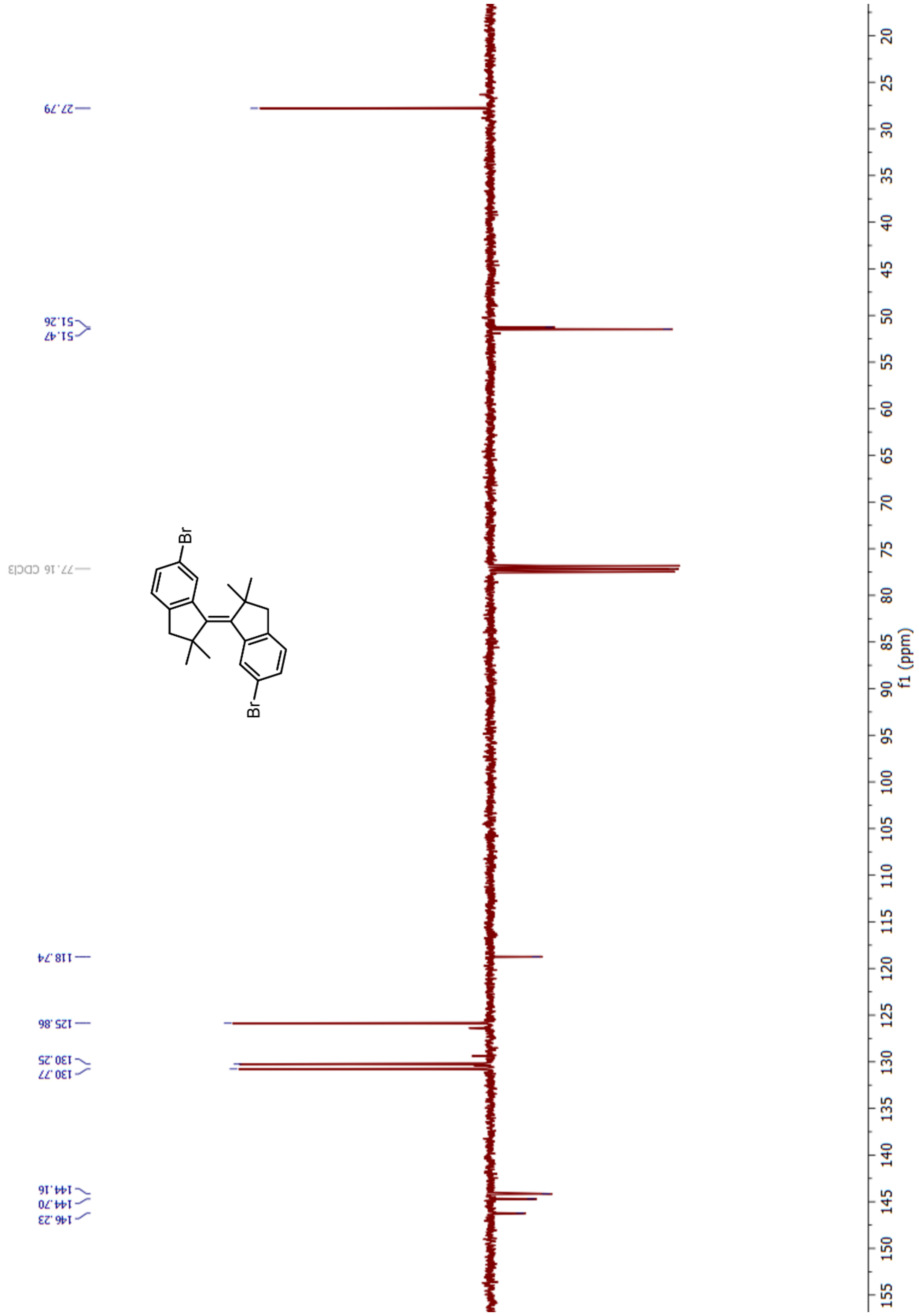

Figure S2. 100 MHz $^{13}C\{^{1}H\}$ APT NMR spectrum of (*E*)-**4** measured at 298 K in $CDCl_3$: CH and CH_3 signals positive and quaternary carbon and CH_2 signals negative.

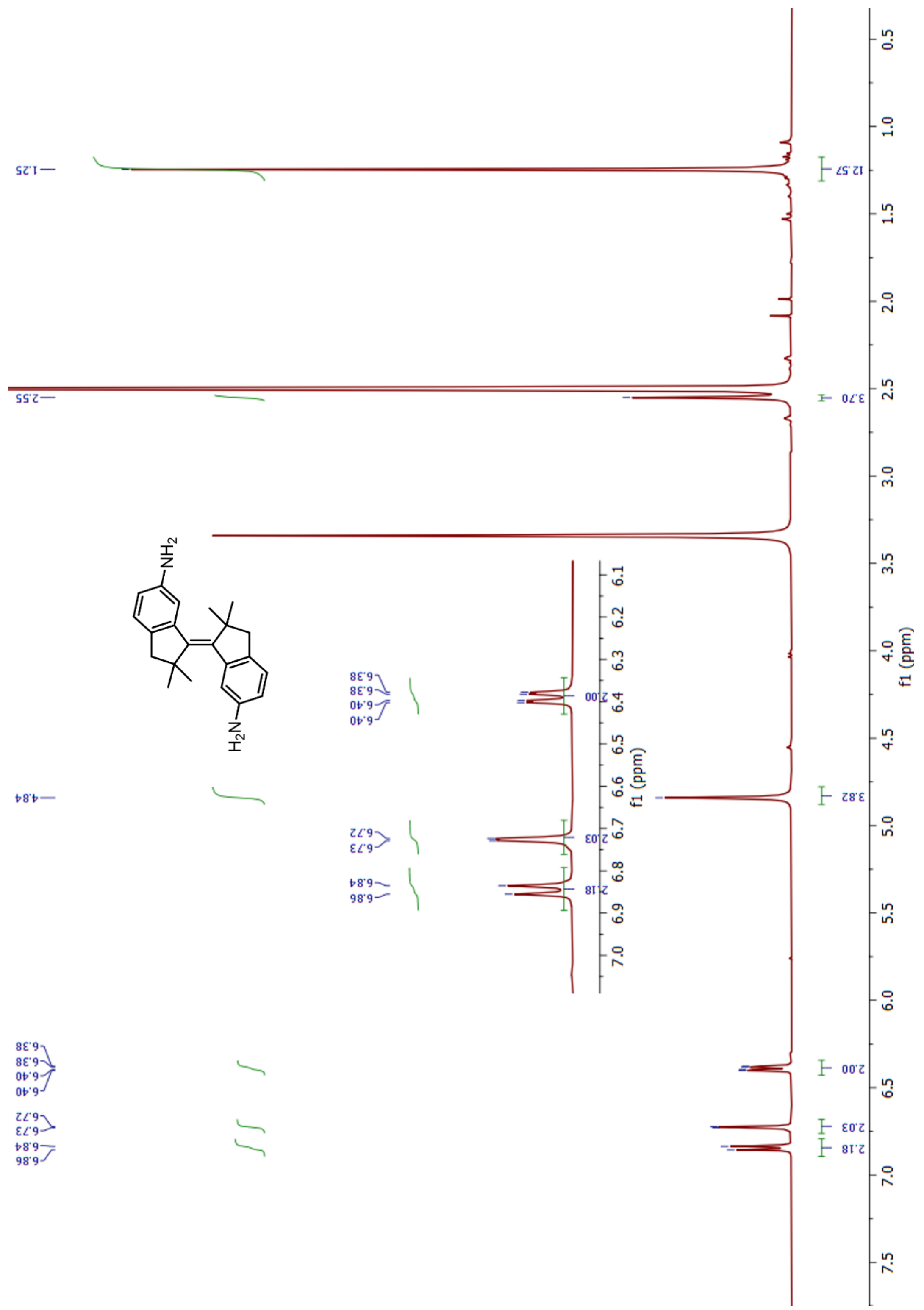

Figure S3. 400 MHz ^{1}H NMR spectrum of (*E*)-**5** measured at 298 K in DMSO-d_6.

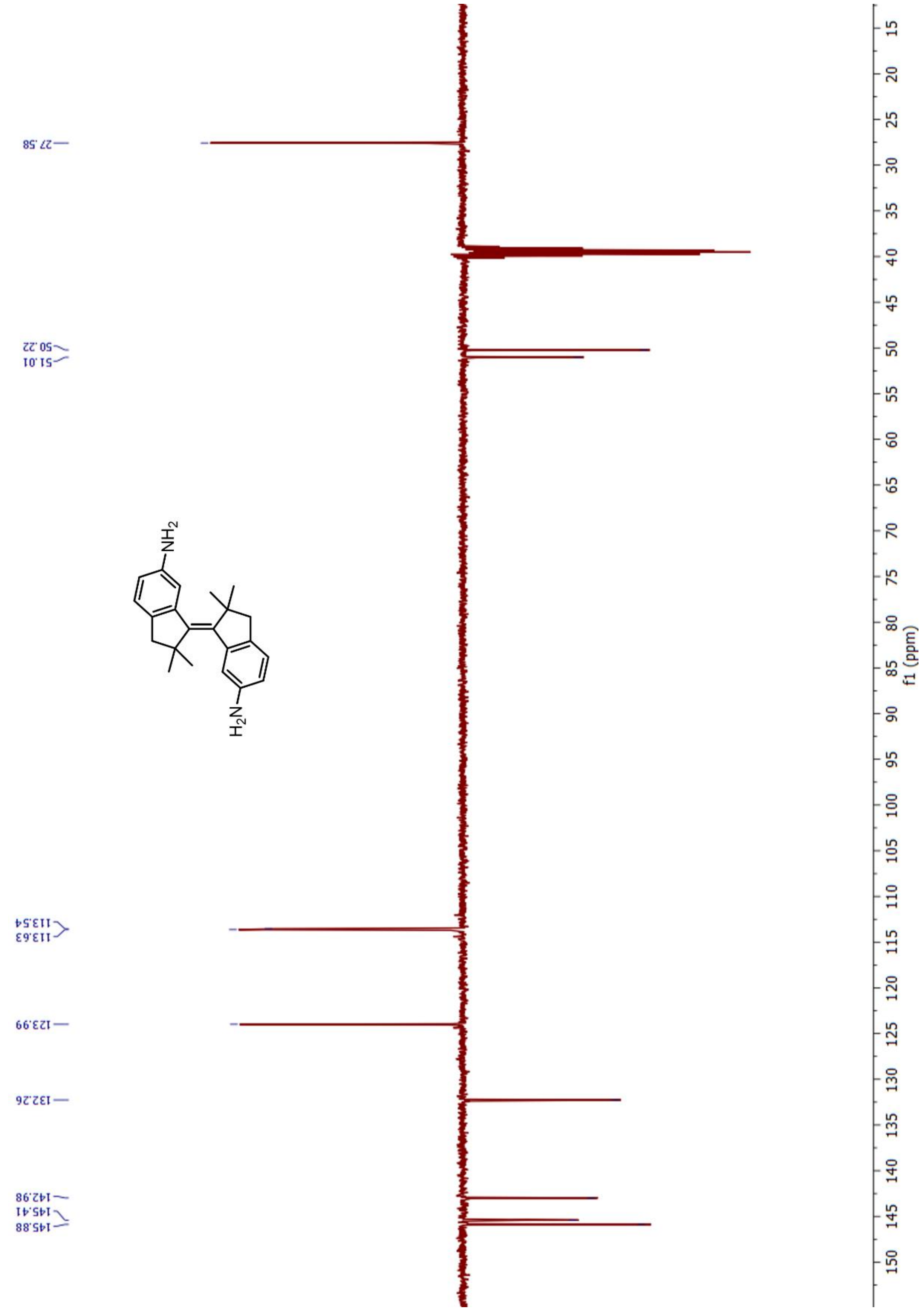

Figure S4. 100 MHz $^{13}C\{^1H\}$ APT NMR spectrum of (*E*)-**5** measured at 298 K in DMSO-d_6: CH and CH_3 signals positive and quaternary carbon and CH_2 signals negative.

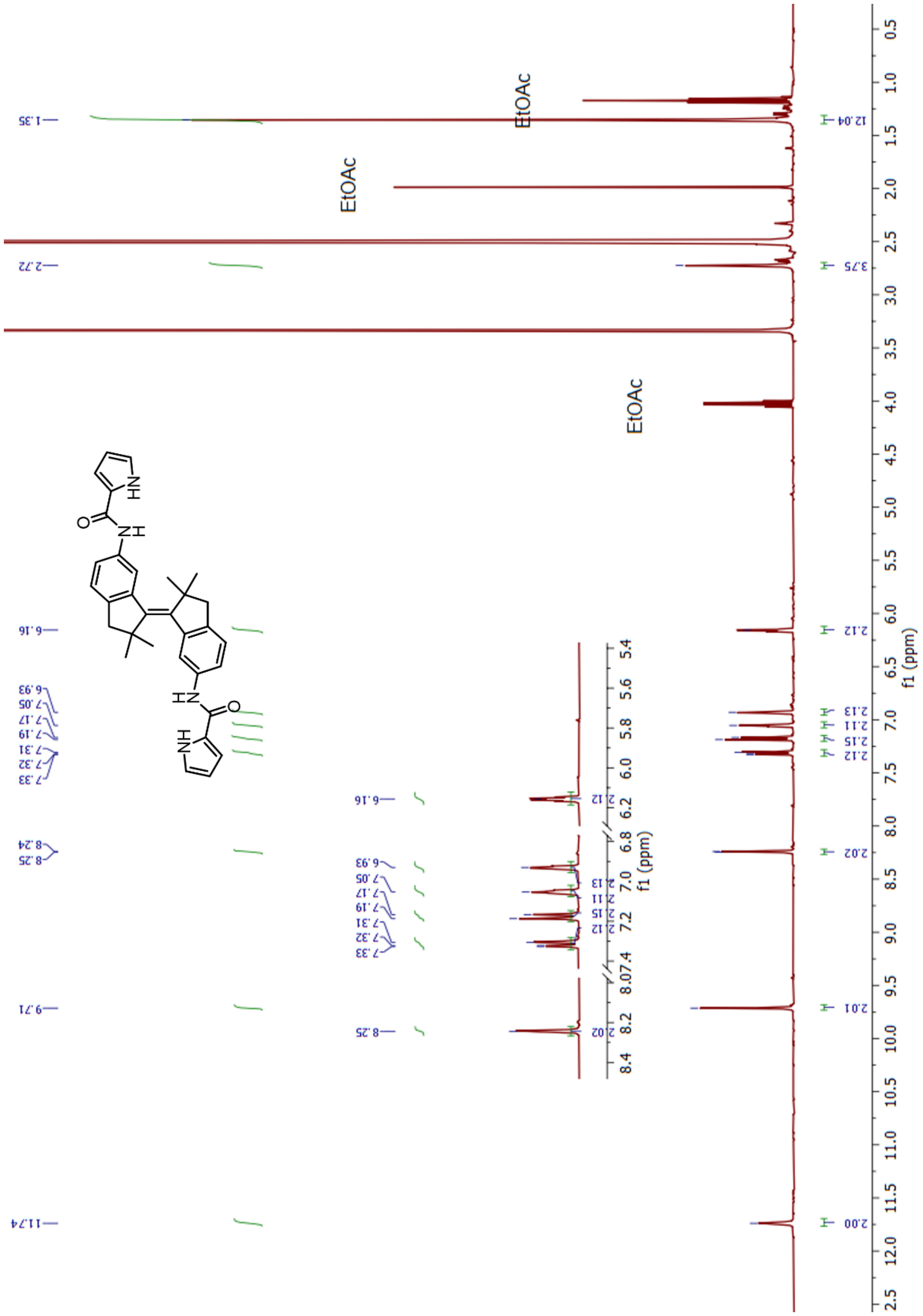

Figure S5. 400 MHz ^{1}H NMR spectrum of (*E*)-**1** measured at 298 K in DMSO-d_6.

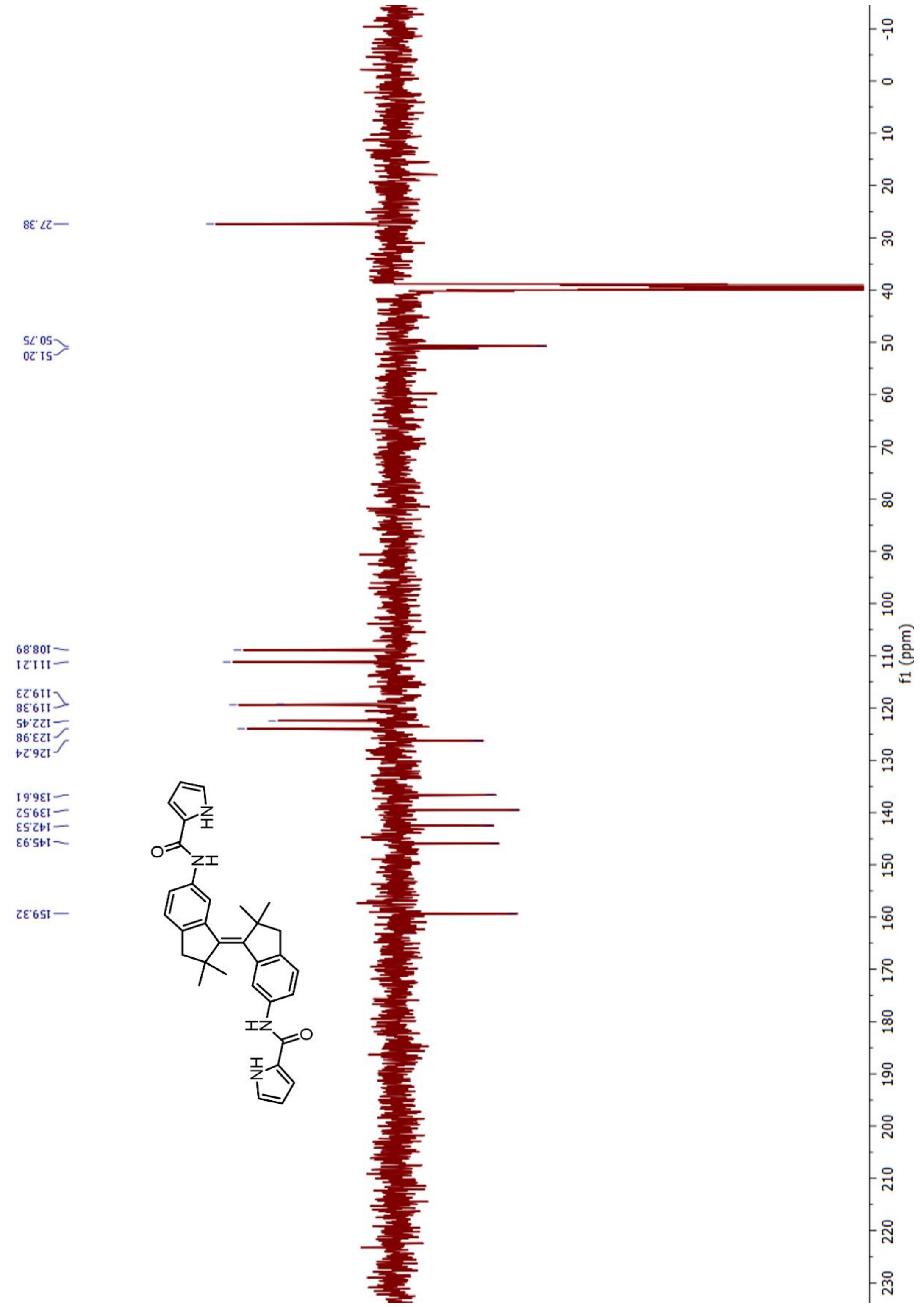

Figure S6. 100 MHz $^{13}C\{^{1}H\}$ APT NMR spectrum of (*E*)-**1** measured at 298 K in DMSO-d_6: CH and CH_3 signals positive and quaternary carbon and CH_2 signals negative.

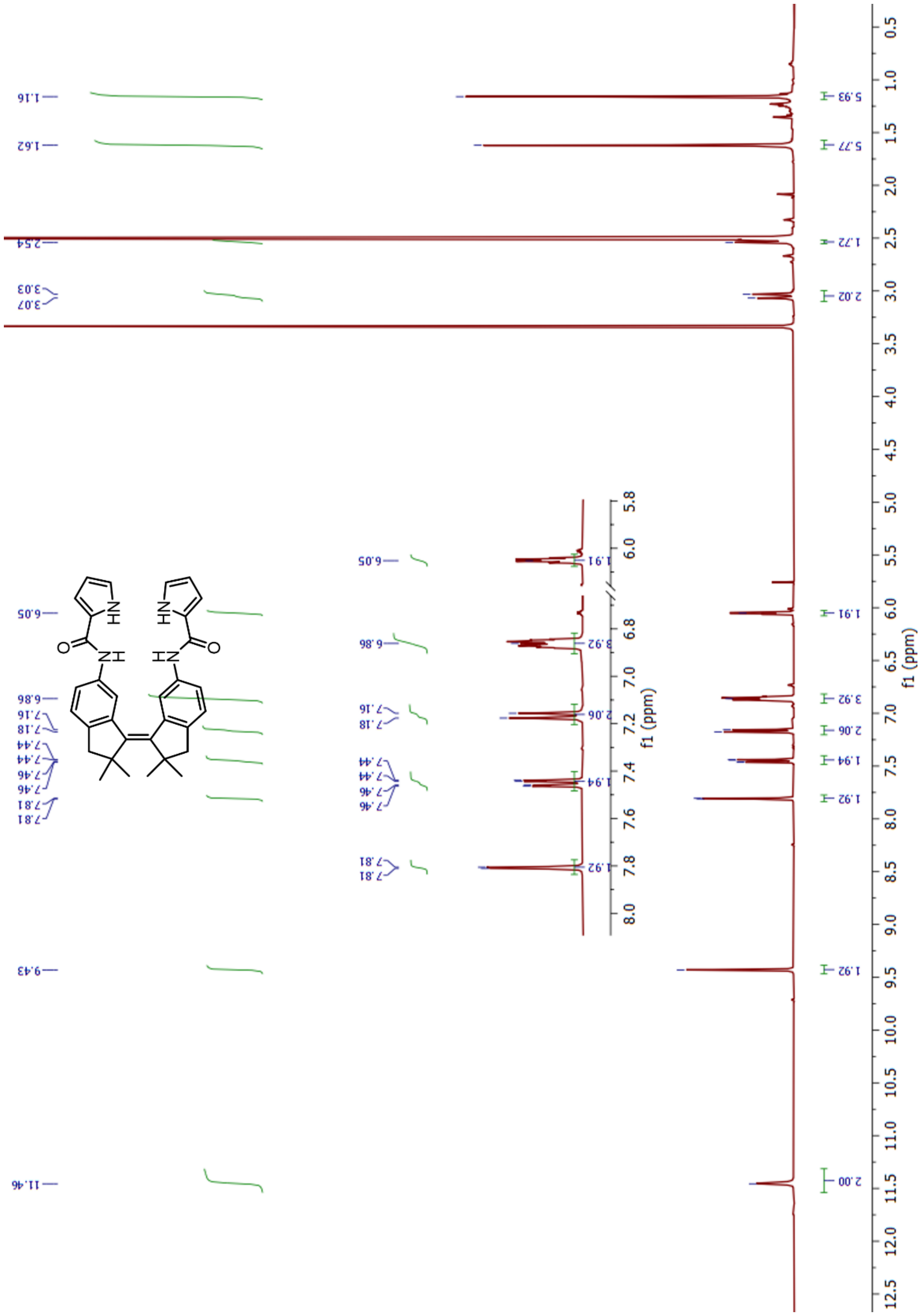

Figure S7. 400 MHz ^{1}H NMR spectrum of (*Z*)-**1** measured at 298 K in DMSO-d_6.

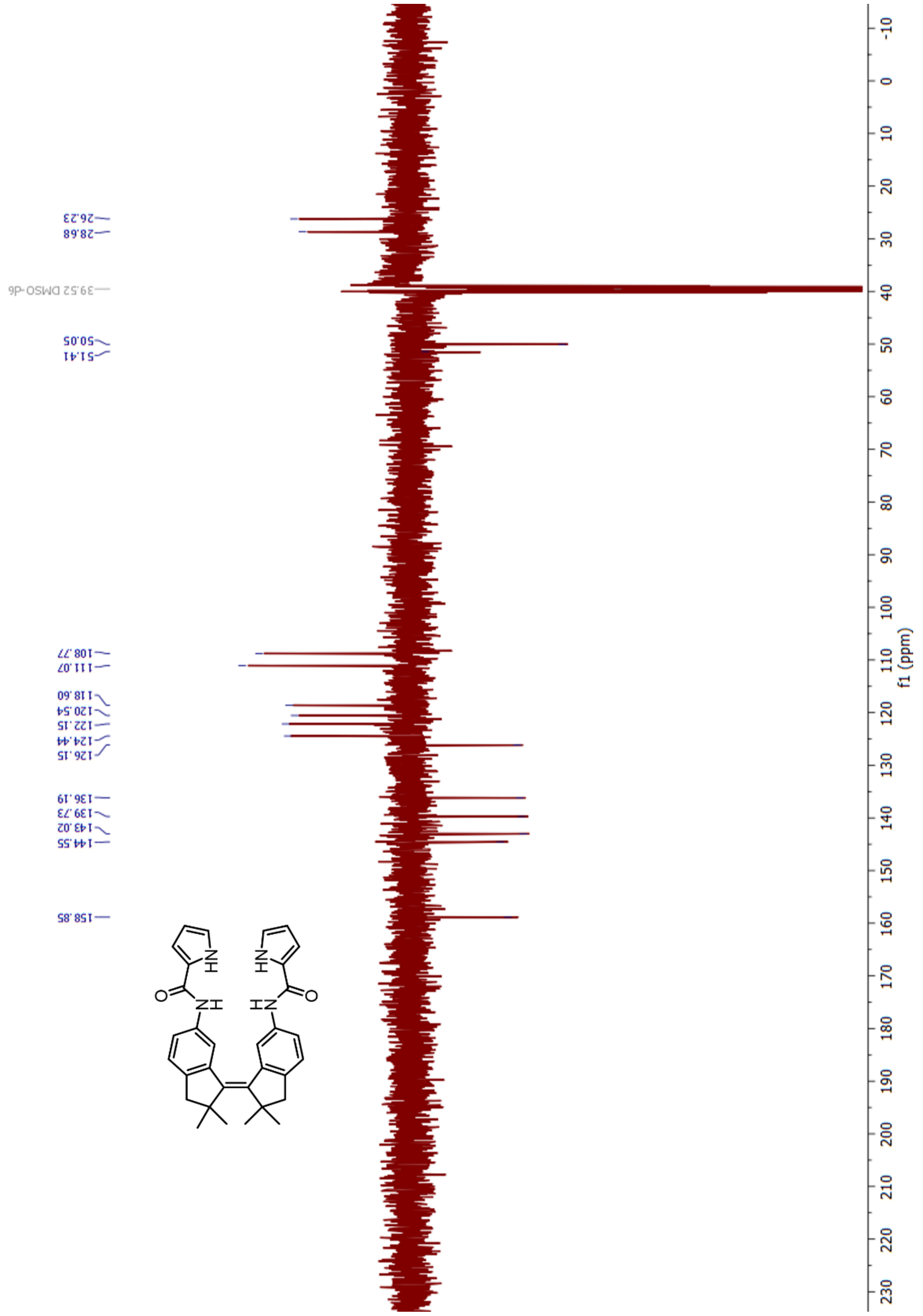

Figure S8. 100 MHz $^{13}C\{^{1}H\}$ APT NMR spectrum of (*Z*)-**1** measured at 298 K in DMSO-d_6: CH and CH_3 signals positive and quaternary carbon and CH_2 signals negative.

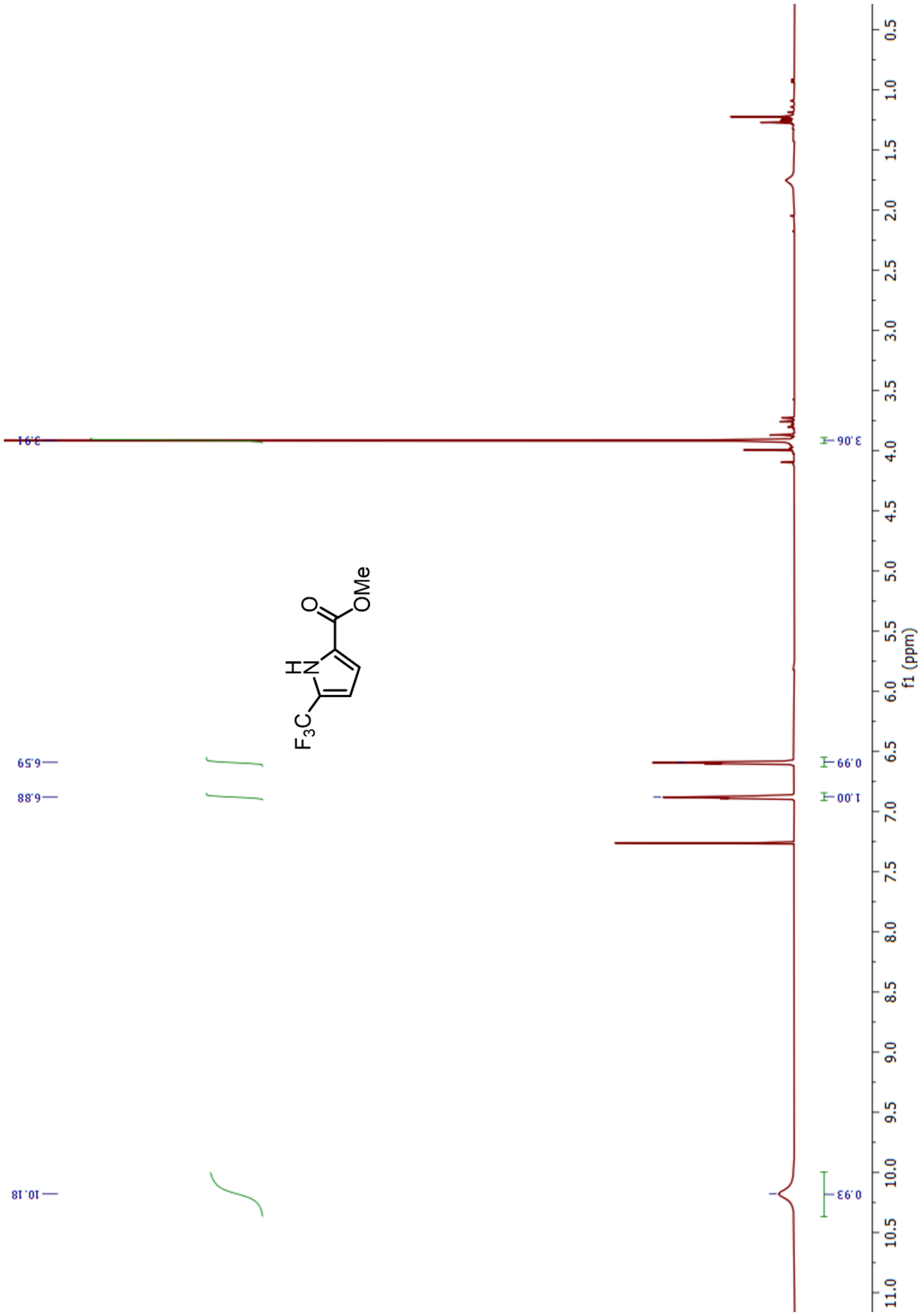

Figure S9. 400 MHz ^{1}H NMR spectrum of **6** measured at 298 K in $CDCl_3$.

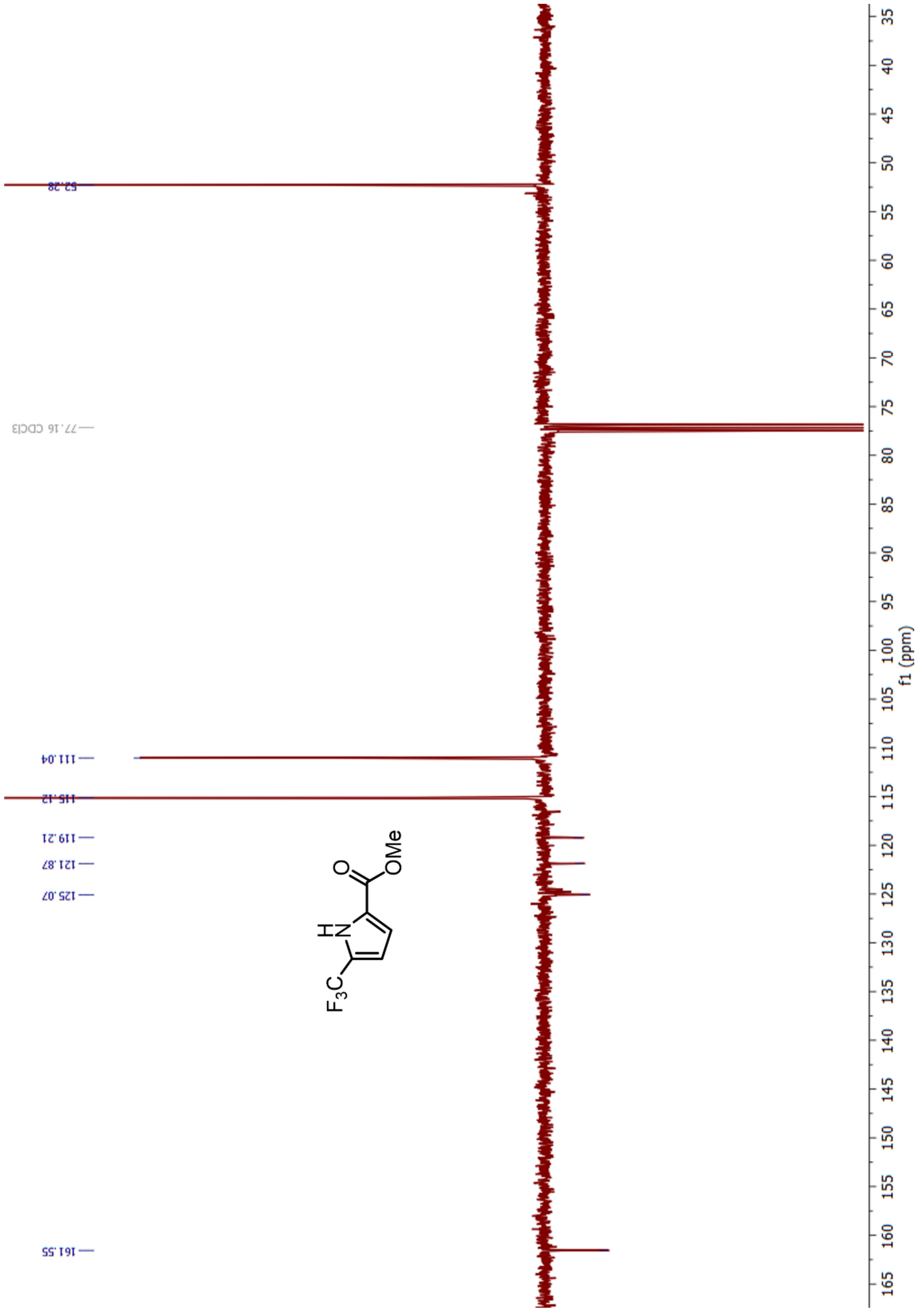

Figure S10. 100 MHz $^{13}C\{^{1}H\}$ APT NMR spectrum of **6** measured at 298 K in $CDCl_3$.

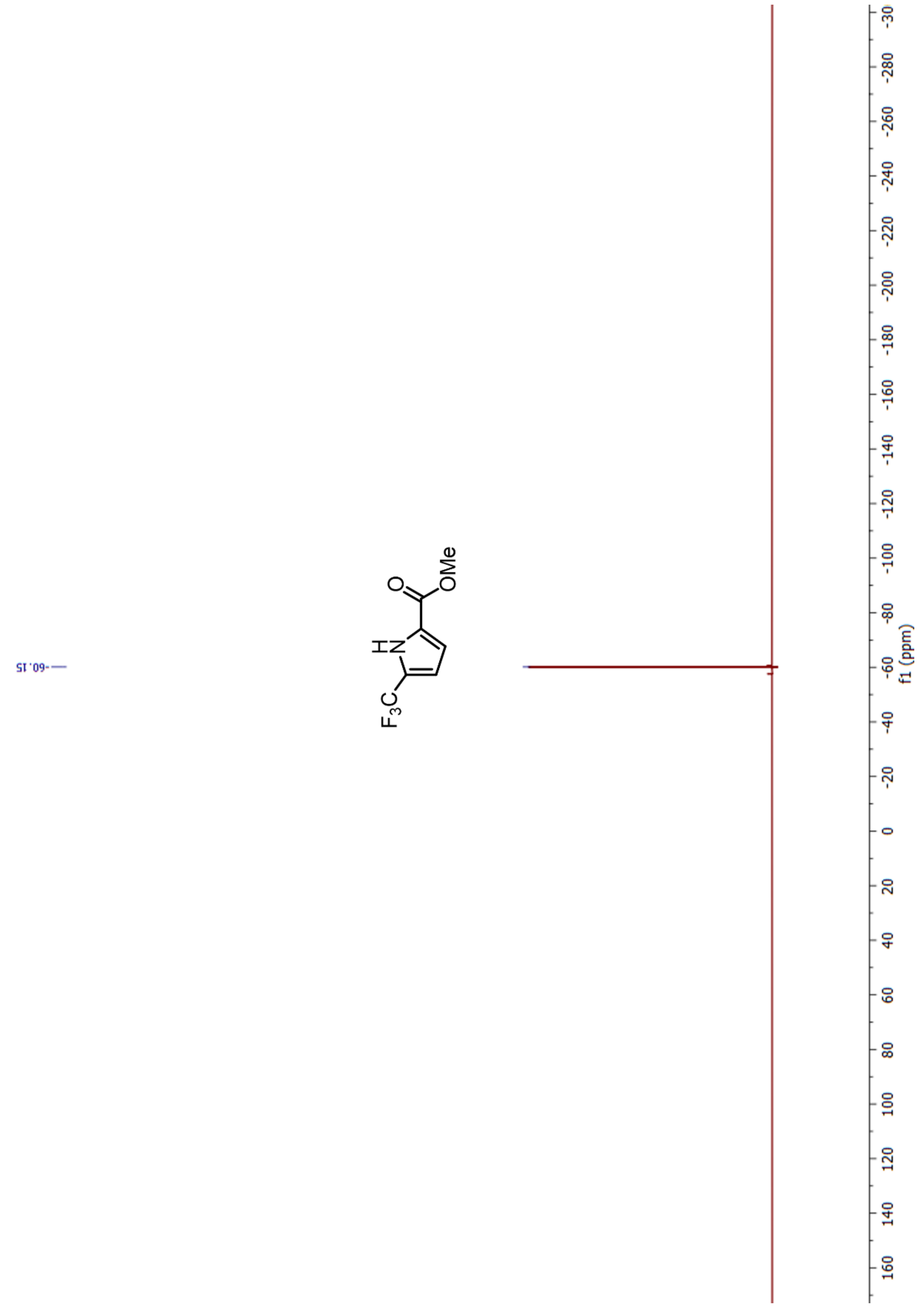

Figure S11. 470 MHz ^{19}F NMR spectrum of **6** measured at 298 K in $CDCl_3$.

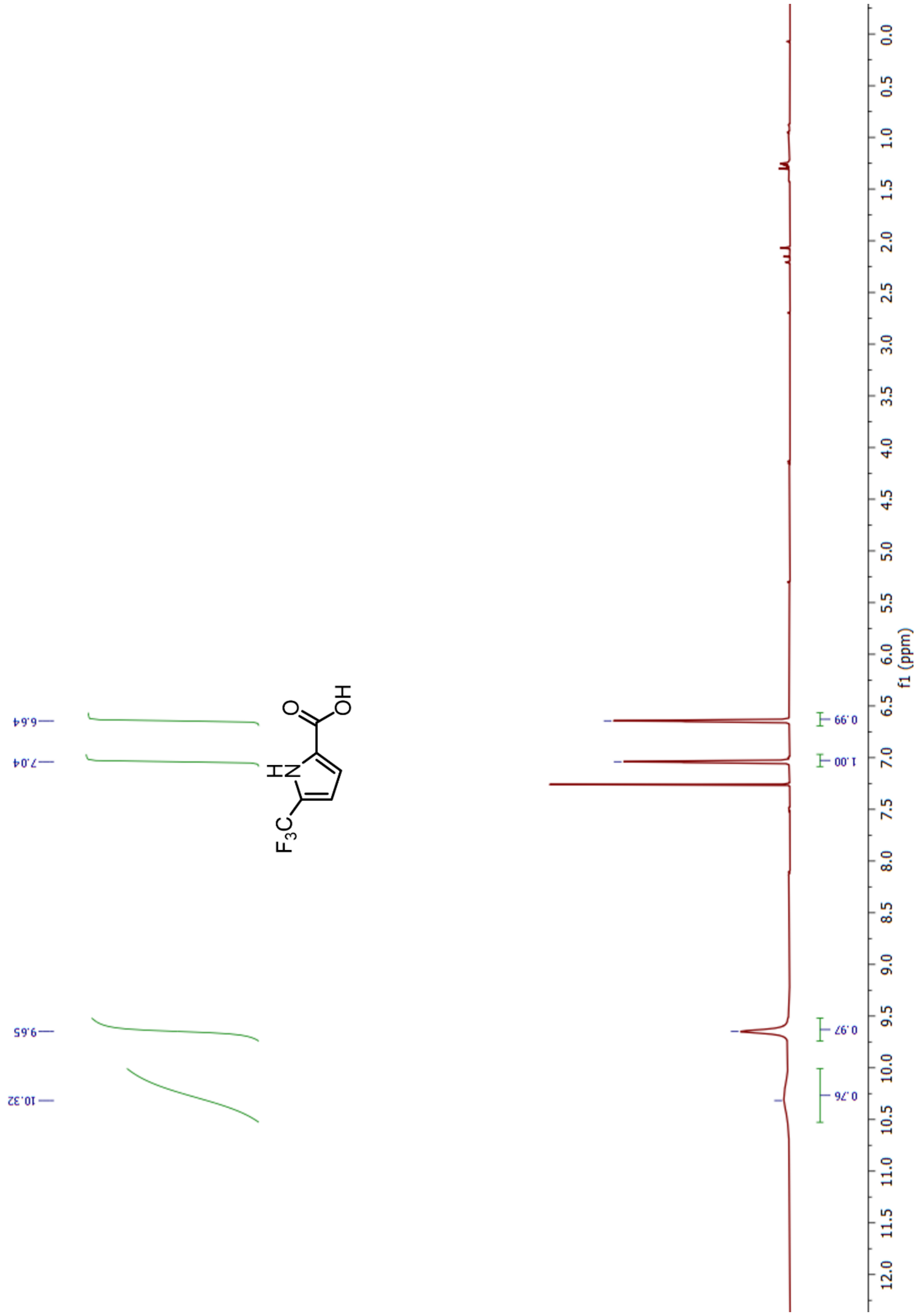

Figure S12. 400 MHz ^{1}H NMR spectrum of **7** measured at 298 K in $CDCl_3$.

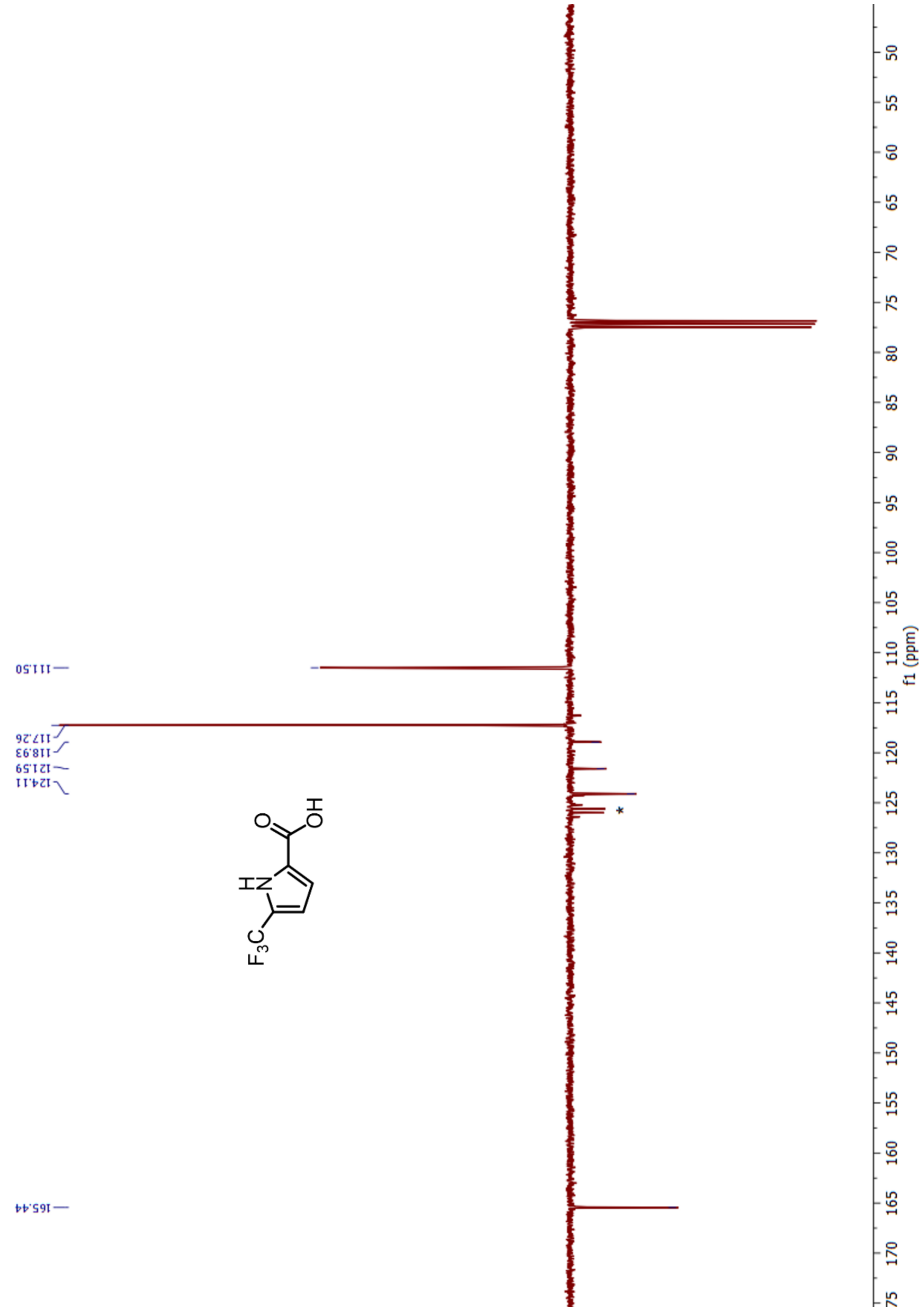

Figure S13. 100 MHz $^{13}C\{^{1}H\}$ APT NMR spectrum of **7** measured at 298 K in $CDCl_3$. (*: impurity)

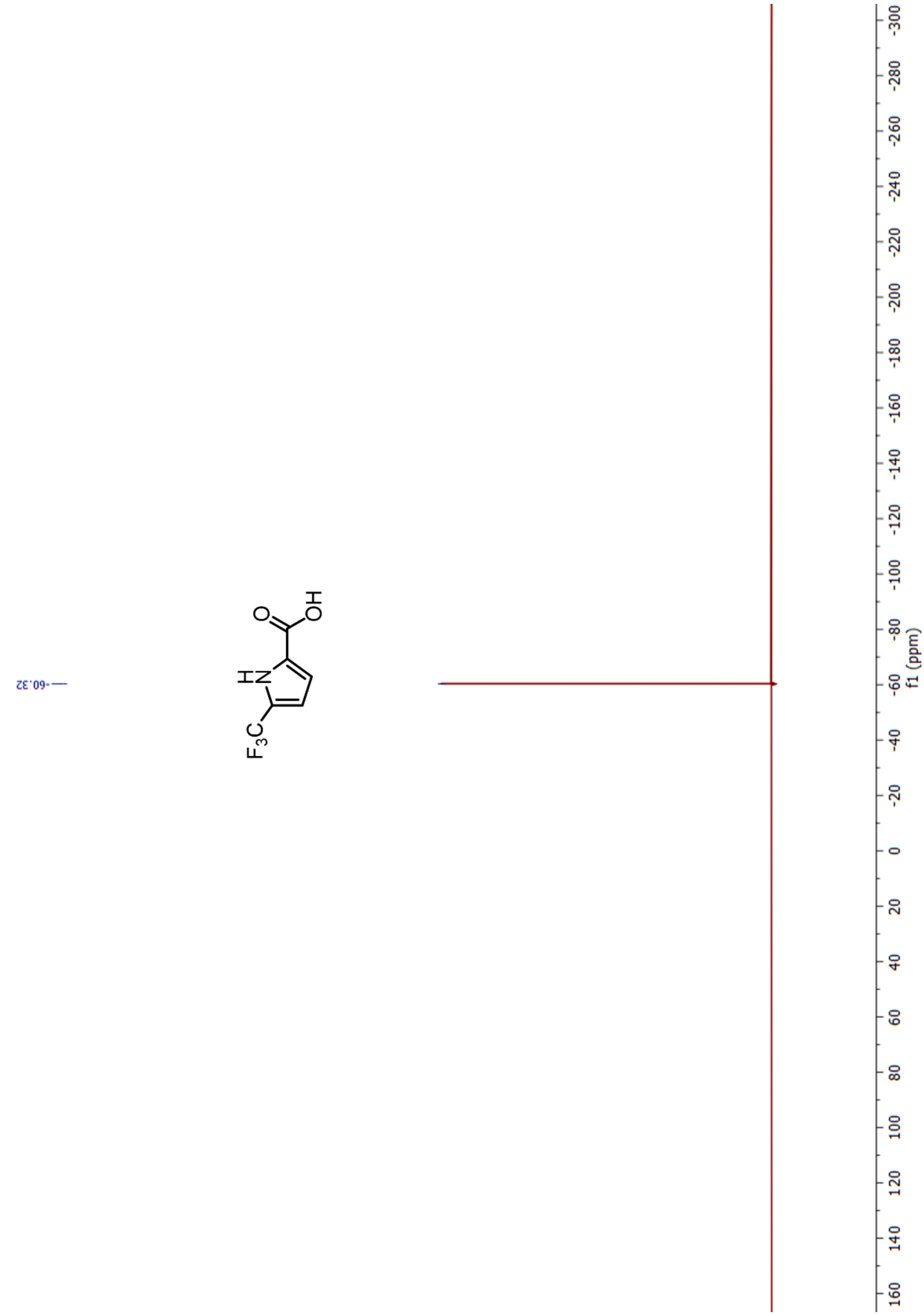

Figure S14. 470 MHz ^{19}F NMR spectrum of **7** measured at 298 K in $CDCl_3$.

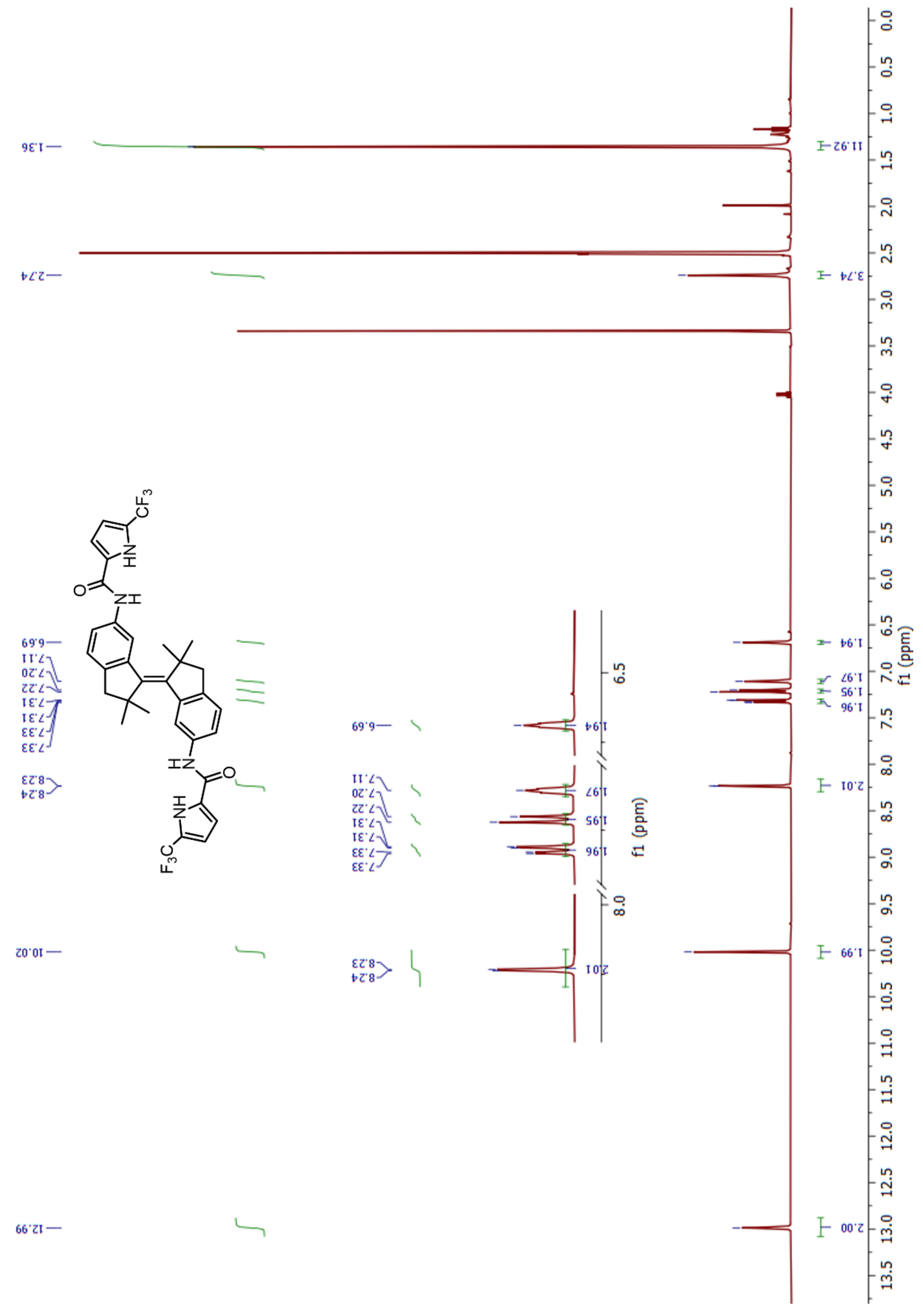

Figure S15. 400 MHz ^{1}H NMR spectrum of (*E*)-**2** measured at 298 K in DMSO-d_6.

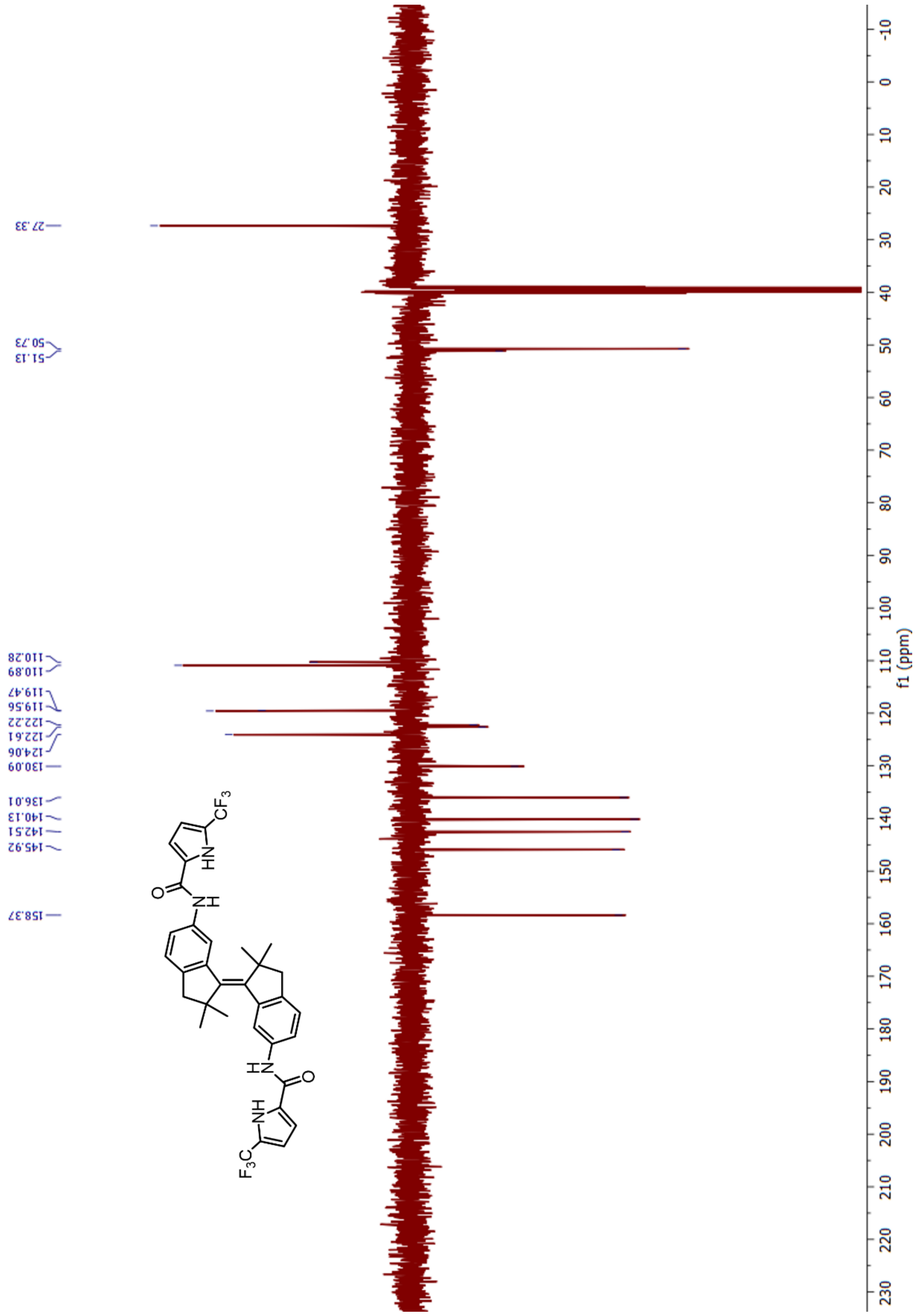

Figure S16. 100 MHz $^{13}C\{^1H\}$ APT NMR spectrum of (*E*)-**2** measured at 298 K in DMSO-d_6: CH and CH_3 signals positive and quaternary carbon and CH_2 signals negative.

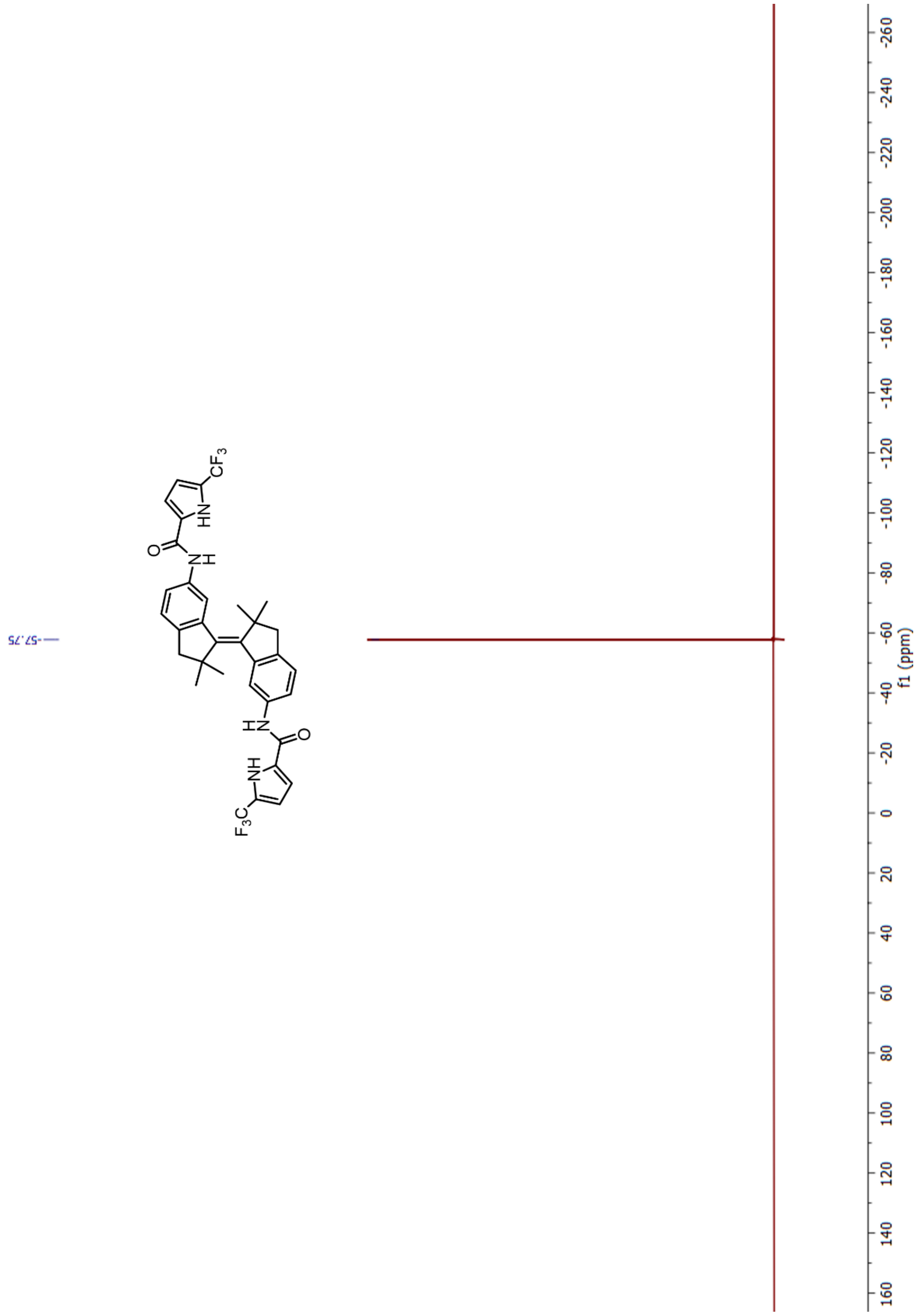

Figure S17. 470 MHz ^{19}F NMR spectrum of (*E*)-**2** measured at 298 K in $CDCl_3$.

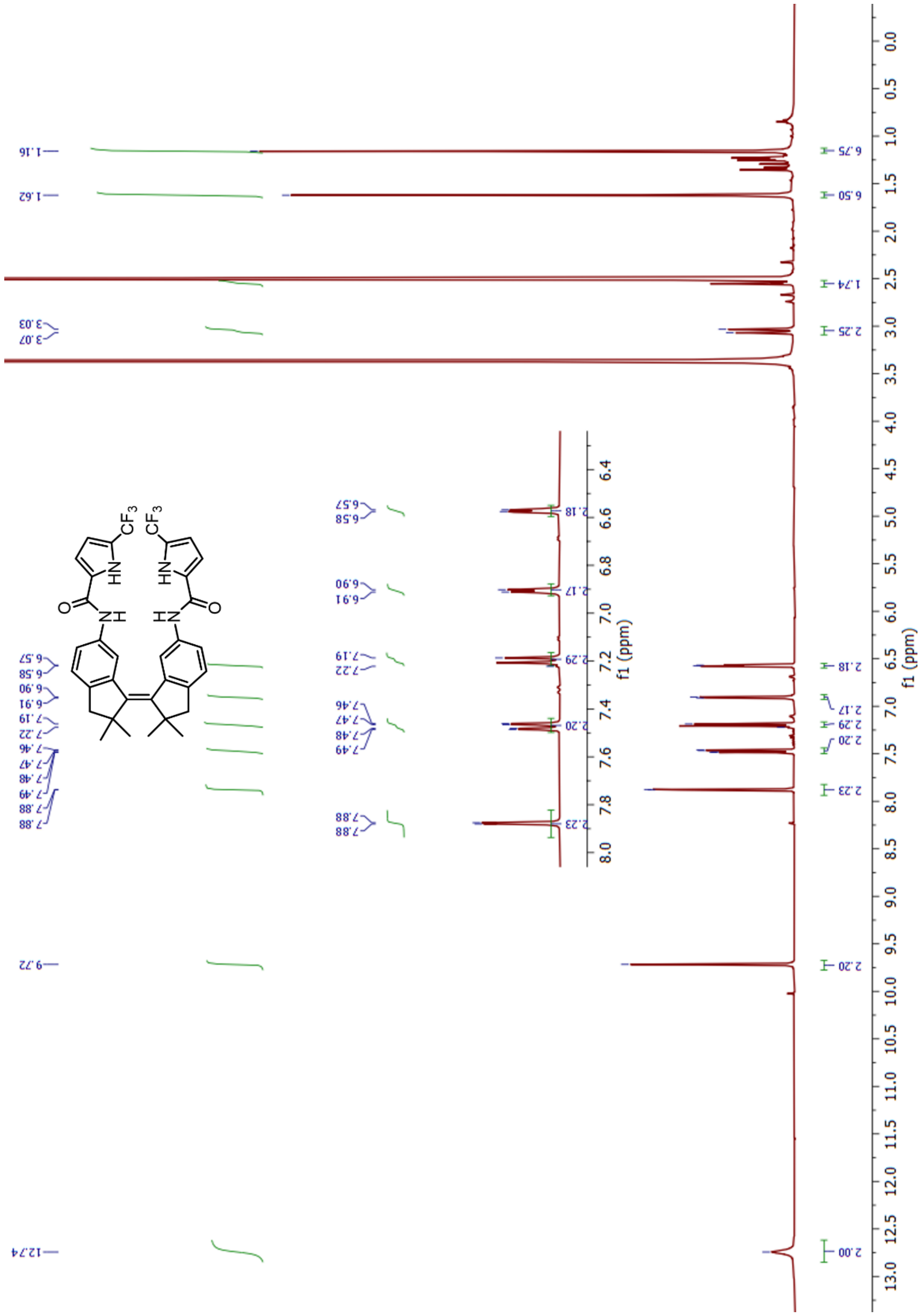

Figure S18. 500 MHz ^{1}H NMR spectrum of (*Z*)-**2** measured at 298 K in DMSO-*d*$_6$.

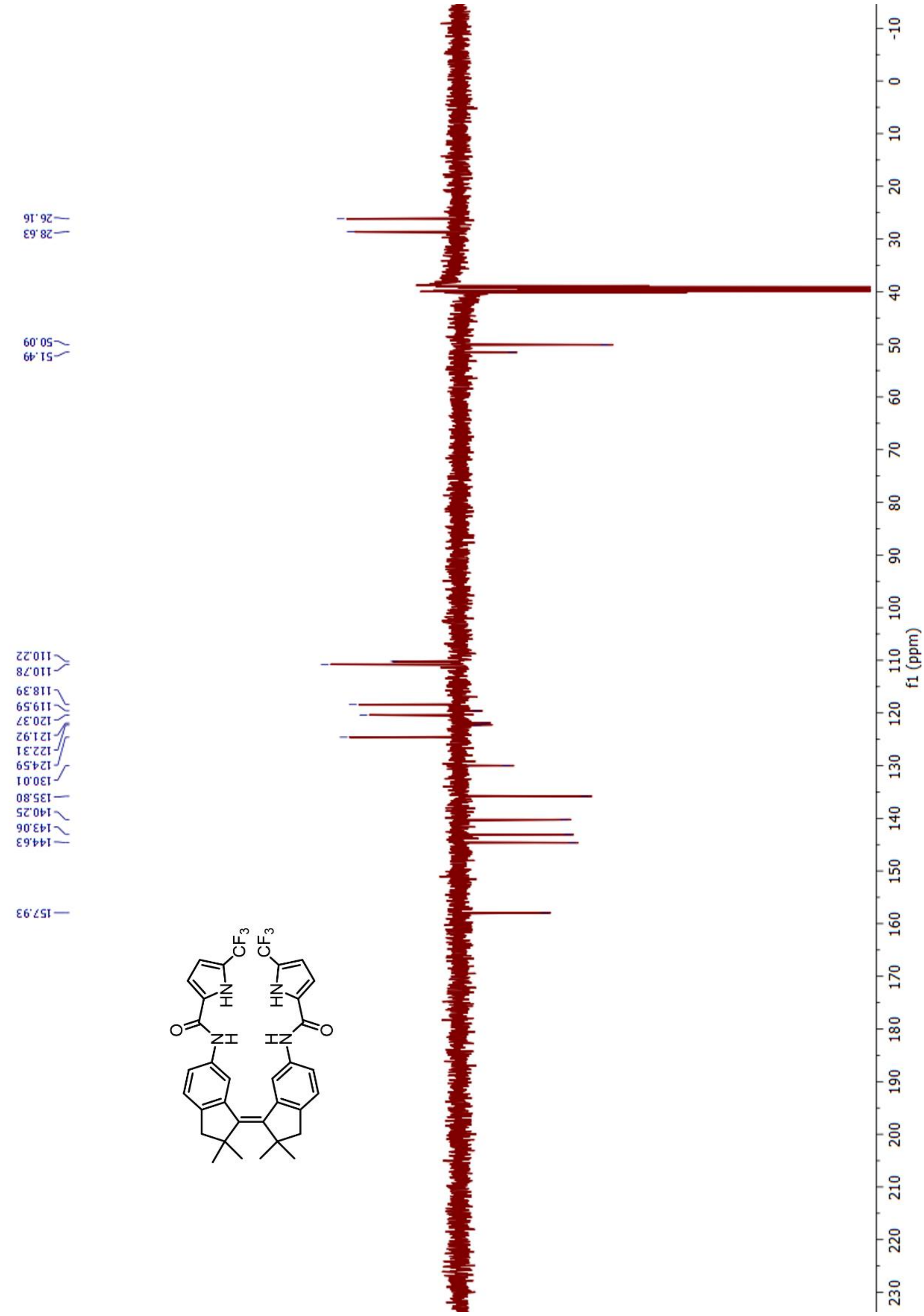

Figure S19. 100 MHz $^{13}C\{^{1}H\}$ APT NMR spectrum of (*Z*)-**2** measured at 298 K in DMSO-d_6: CH and CH_3 signals positive and quaternary carbon and CH_2 signals negative.

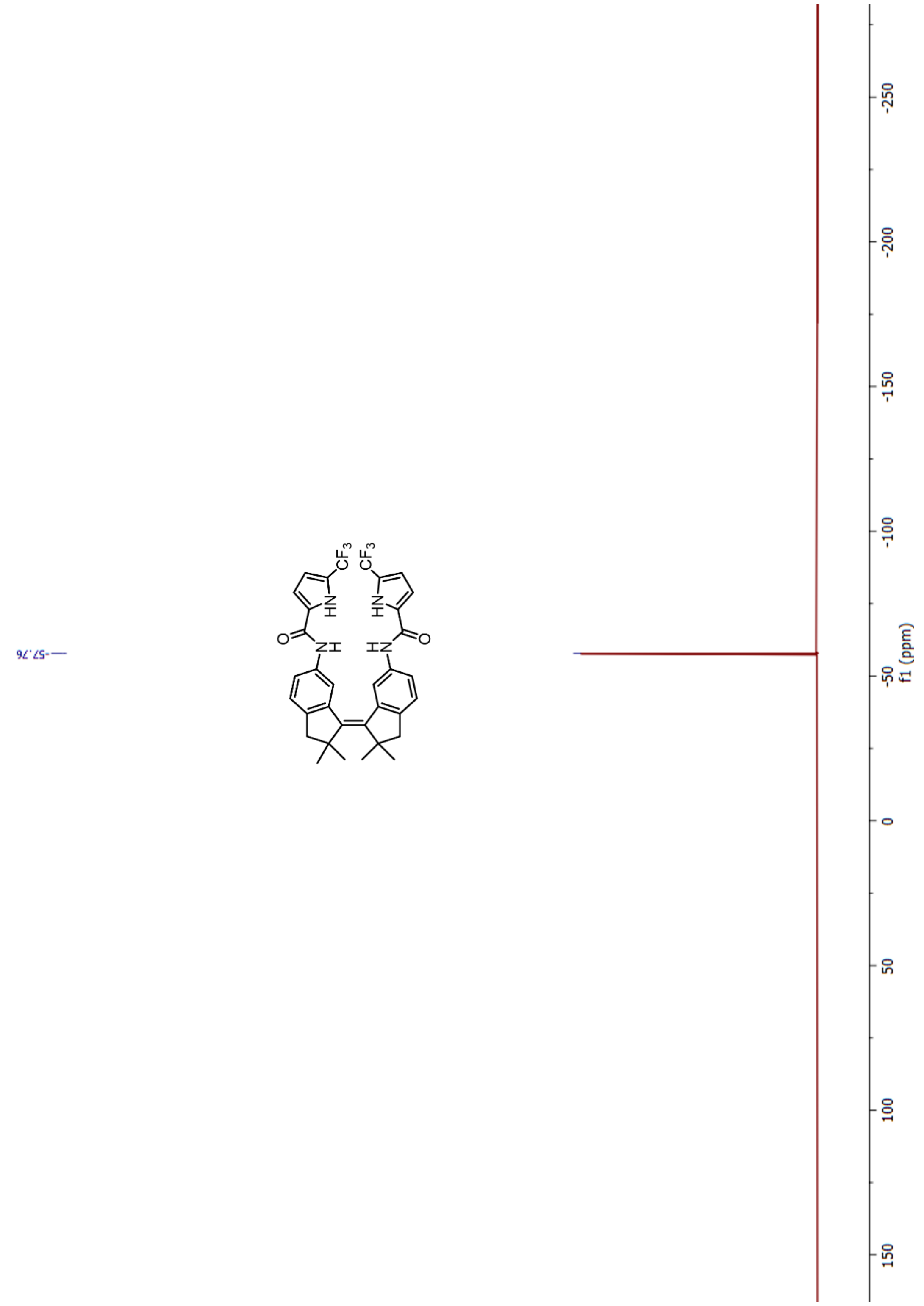

Figure S20. 470 MHz ^{19}F NMR spectrum of (*E*)-**2** measured at 298 K in $CDCl_3$.

UV-vis photoisomerization studies

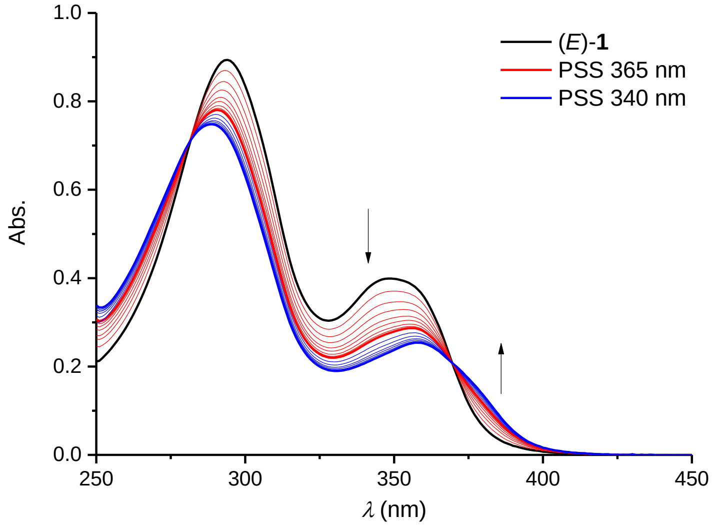

Figure S21. UV-vis spectral changes of (*E*)-**1** (2.0×10^{-4} M in DMSO, 1 mm quartz cuvette) upon 365 nm (10 s) and 340 nm (140 s) irradiation showing an isosbestic point at $\lambda = 372$ nm.

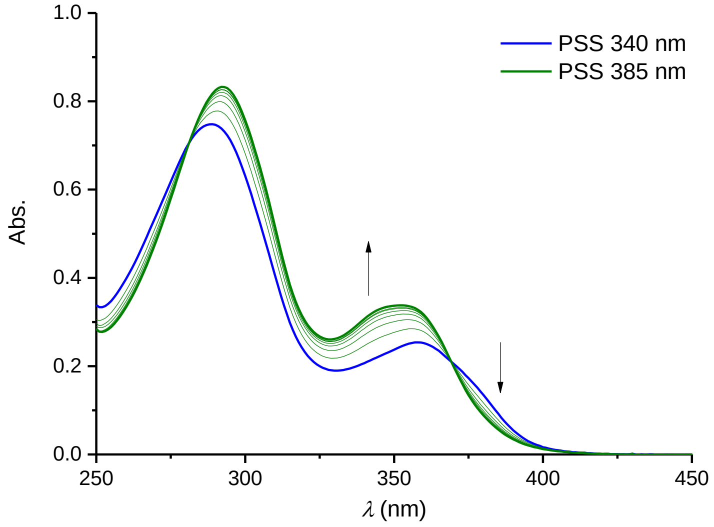

Figure S22. UV-vis spectral changes starting with the PSS_{340} mixture (shown above) upon 385 nm irradiation (180s) showing that the isosbestic point at $\lambda = 372$ nm is maintained.

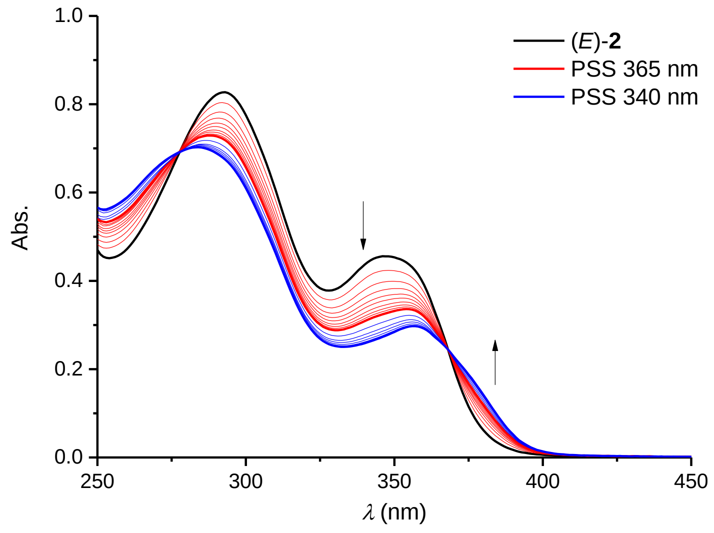

Figure S23. UV-vis spectral changes of (*E*)-**2** (2.0×10^{-4} M in DMSO, 1 mm quartz cuvette) upon 365 nm (10 s) and 340 nm (120 s) irradiation showing an isosbestic point at $\lambda = 370$ nm.

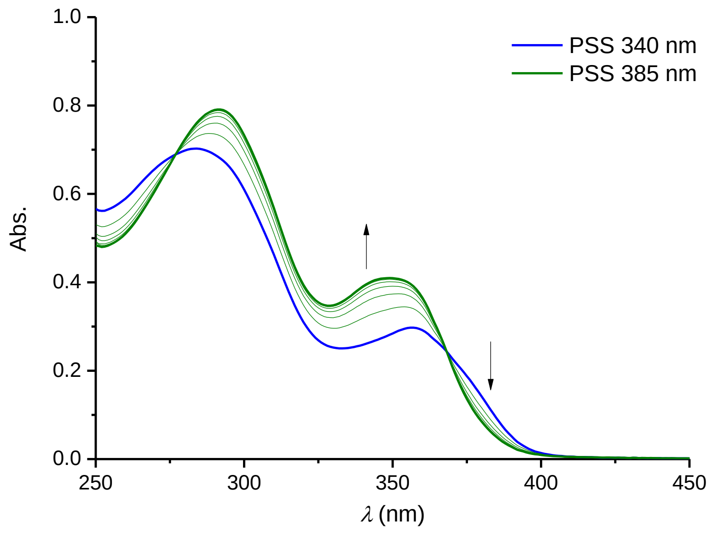

Figure S24. UV-vis spectral changes starting with the PSS_{340} mixture (shown above) upon 385 nm irradiation (120s) showing that the isosbestic point at $\lambda = 370$ nm is maintained.

^{1}H NMR photoisomerization studies

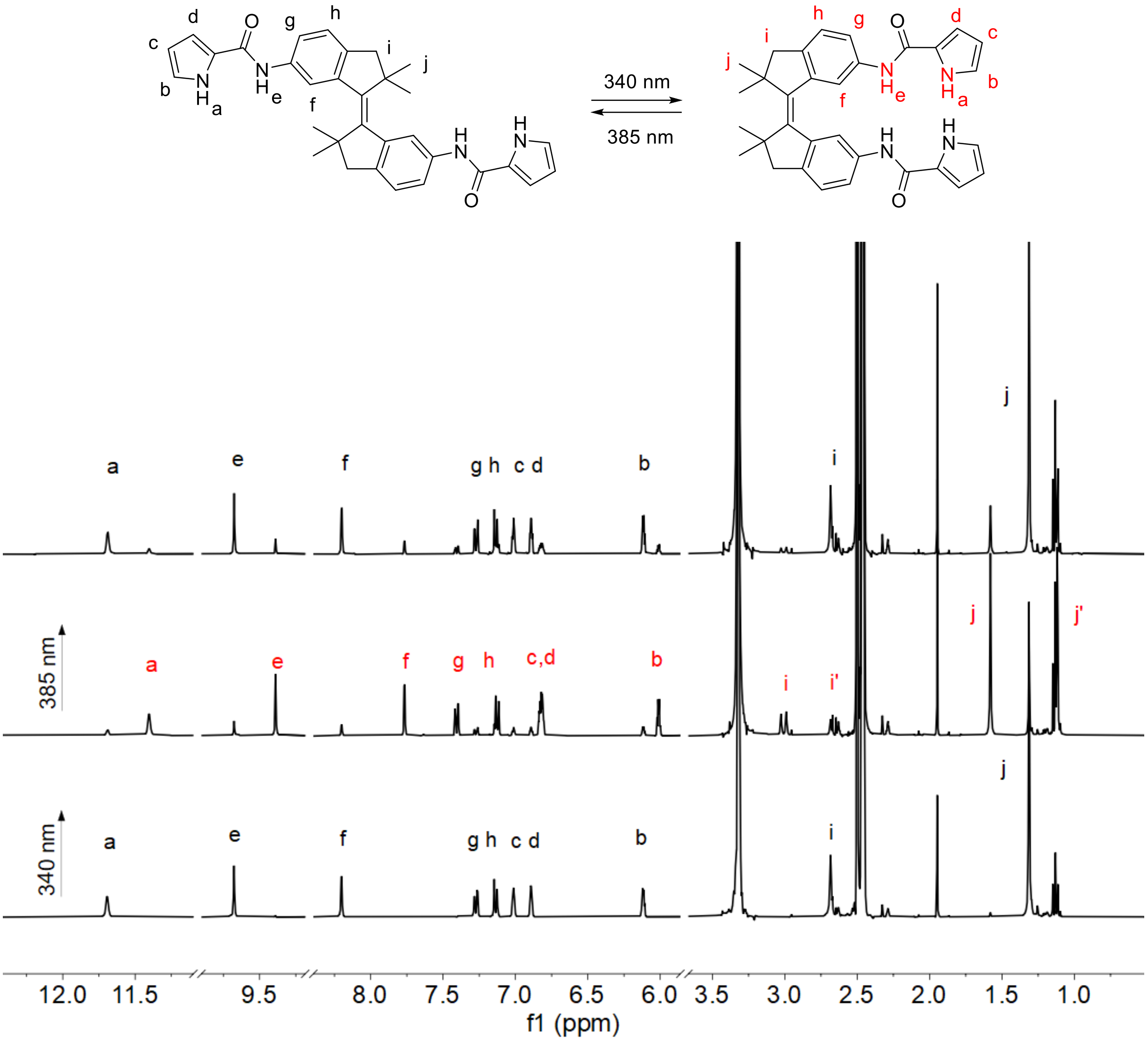

Figure S25. ^{1}H NMR spectrum (400 MHz, 293 K) of (*E*)-**1** in degassed DMSO-d_6 (4 mM) before (bottom) and after irradiation with 340 nm light for 4 h (middle), followed by irradiation with 385 nm light for 2 h (top). For determination of the PSS ratios, the integrals of the H_a, H_b, H_c, H_d, H_e, H_f and H_g signals were averaged giving a PSS_{340} ratio (*E*/*Z*) of 19:81 and a PSS_{385} ratio (*E*/*Z*) of 82:18. Assignment of protons is based on 2D NMR spectroscopy.

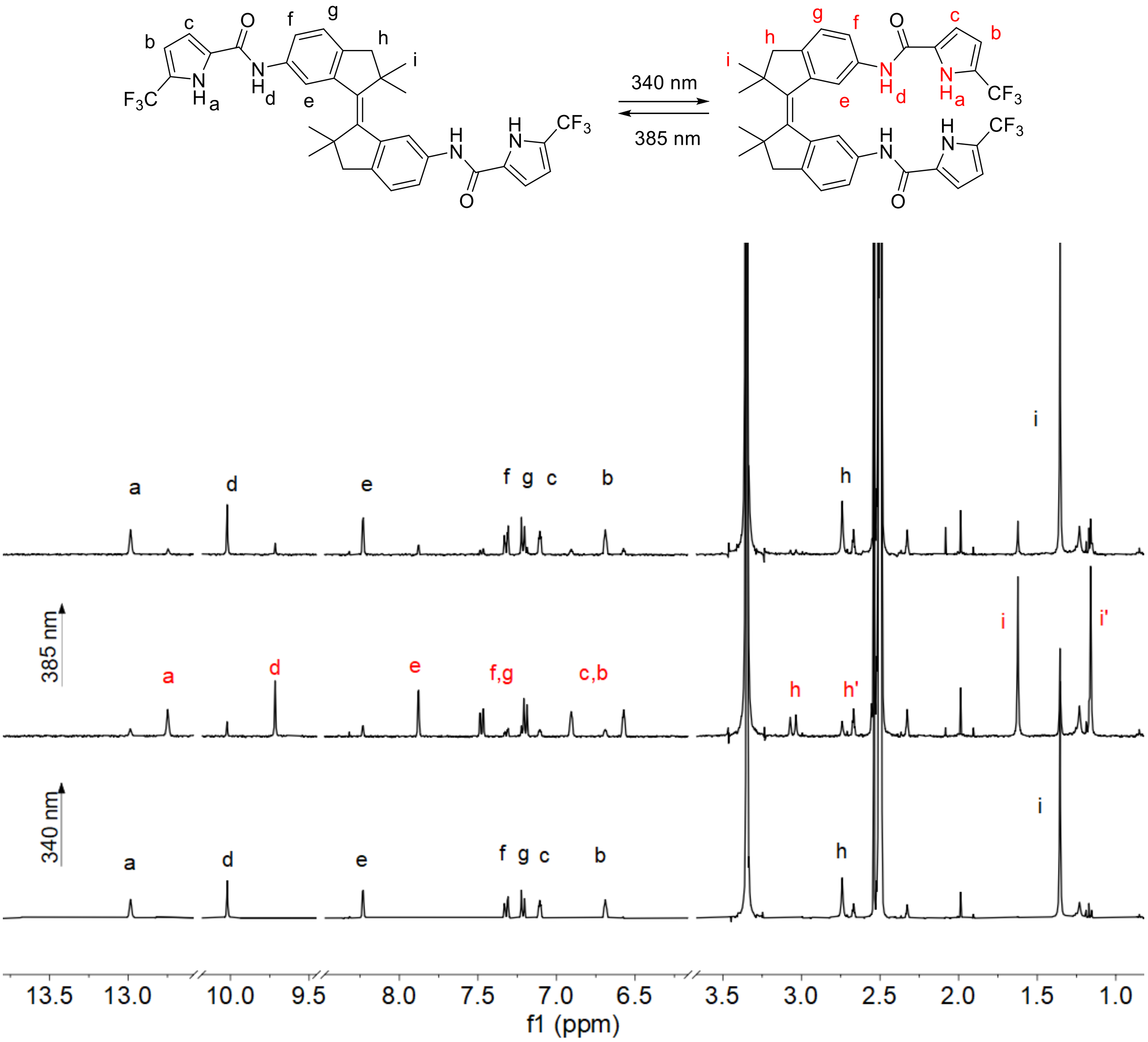

Figure S26. ^{1}H NMR Spectrum (400 MHz, 293 K) of (*E*)-**2** in degassed DMSO-d_6 (2 mM) before (bottom) and after irradiation with 340 nm light for 3 h (middle), followed by irradiation with 385 nm light for 2 h (top). For determination of the PSS ratios, the integrals of the H_a, H_b, H_c, H_d, H_e, H_f and H_g signals were averaged giving a PSS_{340} ratio (*E*/*Z*) of 23:77 and a PSS_{385} ratio (*E*/*Z*) of 83:17. Assignment of protons is based on 2D NMR spectroscopy.

^{1}H NMR titration experiments

First, a solution of bis-amidopyrrole receptor was prepared in DMSO-d_6/0.5%H_2O (concentrations are specified in the captions). This solution was then used to prepare the $[Bu_4N]^+[Cl]^-$ or $[Bu_4N]^+[CH_3COO]^-$ solution, which was added stepwise to 0.5 mL of the receptor solution. After each addition a ^{1}H NMR spectrum (500 MHz) was recorded.

Addition of $[Bu_4N]^+[CH_3COO]^-$ to (*Z*)-1:

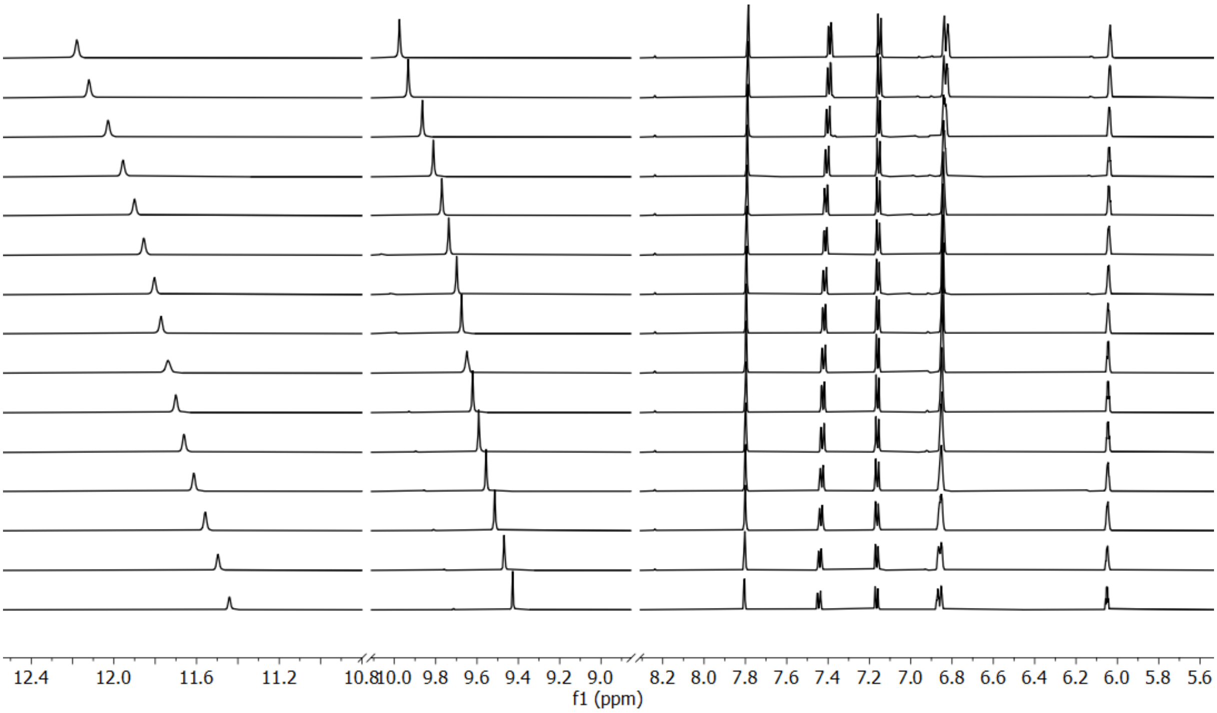

Figure S27. ^{1}H NMR spectral changes (500 MHz, 293 K) of (*Z*)-**1** in DMSO-d_6/0.5%H_2O (2.3 mM) upon the stepwise addition of $[Bu_4N]^+[CH_3COO]^-$ (from bottom to top: 0.00, 0.26, 0.56, 0.82, 1.04, 1.30, 1.52, 1.73, 1.95, 2.38, 2.77, 3.29, 4.07, 5.33, 6.33 equivalents).

Addition of $[Bu_4N]^+[CH_3COO]^-$ to (*Z*)-2:

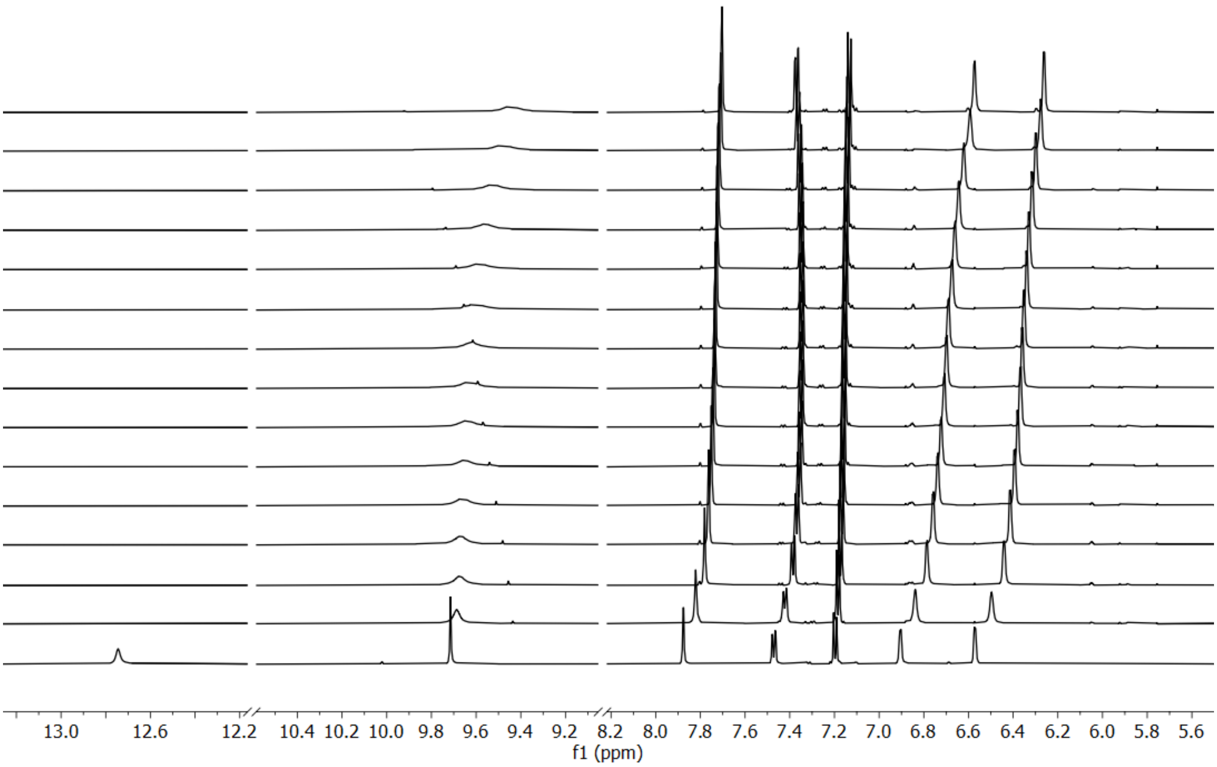

Figure S28. ^1H NMR spectral changes (500 MHz, 293 K) of (*Z*)-**2** in DMSO-d_6/0.5%H_2O (2.1 mM) upon the stepwise addition of $[Bu_4N]^+[CH_3COO]^-$ (from bottom to top: 0.00, 0.33, 0.65, 0.95, 1.24, 1.52, 1.80, 2.06, 2.31, 2.80, 3.25, 3.87, 4.79, 6.29, 7.45 equivalents).

Addition of $[Bu_4N]^+[Cl]^-$ to (*Z*)-1:

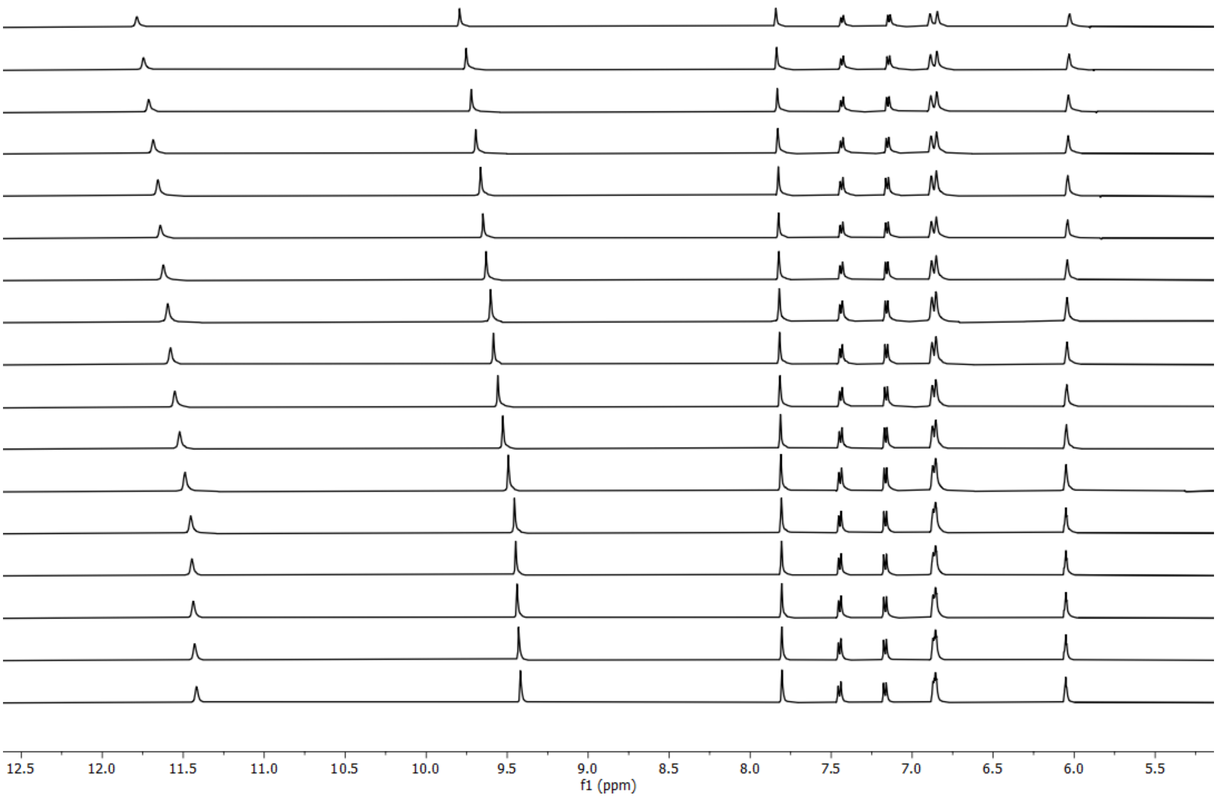

Figure S29. ^{1}H NMR spectral changes (500 MHz, 293 K) of (*Z*)-**1** in DMSO-d_6/0.5%H_2O (5.0 mM) upon the stepwise addition of $[Bu_4N]^+[Cl]^-$ (from bottom to top: 0.00, 0.69, 1.33, 1.93, 2.48, 5.49, 8.40, 13.9, 16.6, 19.1, 21.6, 24.0, 26.3, 28.6, 32.9, 39.0, 48.0 equivalents).

Addition of $[Bu_4N]^+[Cl]^-$ to (*Z*)-2:

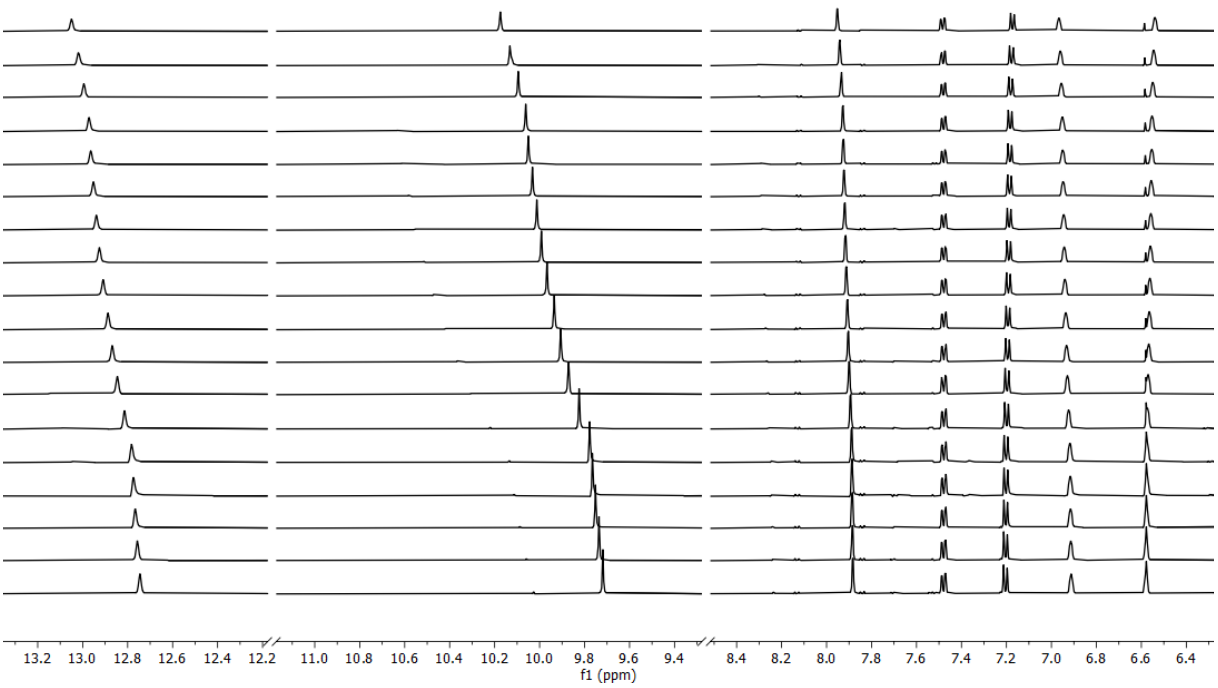

Figure S30. ^{1}H NMR spectral changes (500 MHz, 293 K) of (*Z*)-**2** in DMSO-d_6/0.5%H_2O (5.0 mM) upon the stepwise addition of $[Bu_4N]^+[Cl]^-$ (from bottom to top: 0.00, 0.69, 1.33, 1.93, 2.49, 5.49, 8.40, 11.2, 13.9, 16.6, 19.1, 21.6, 24.0, 26.3, 28.6, 32.9, 39.0, 48.0 equiv.).

Addition of $[Bu_4N]^+[CH_3COO]^-$ to (*E*)-1:

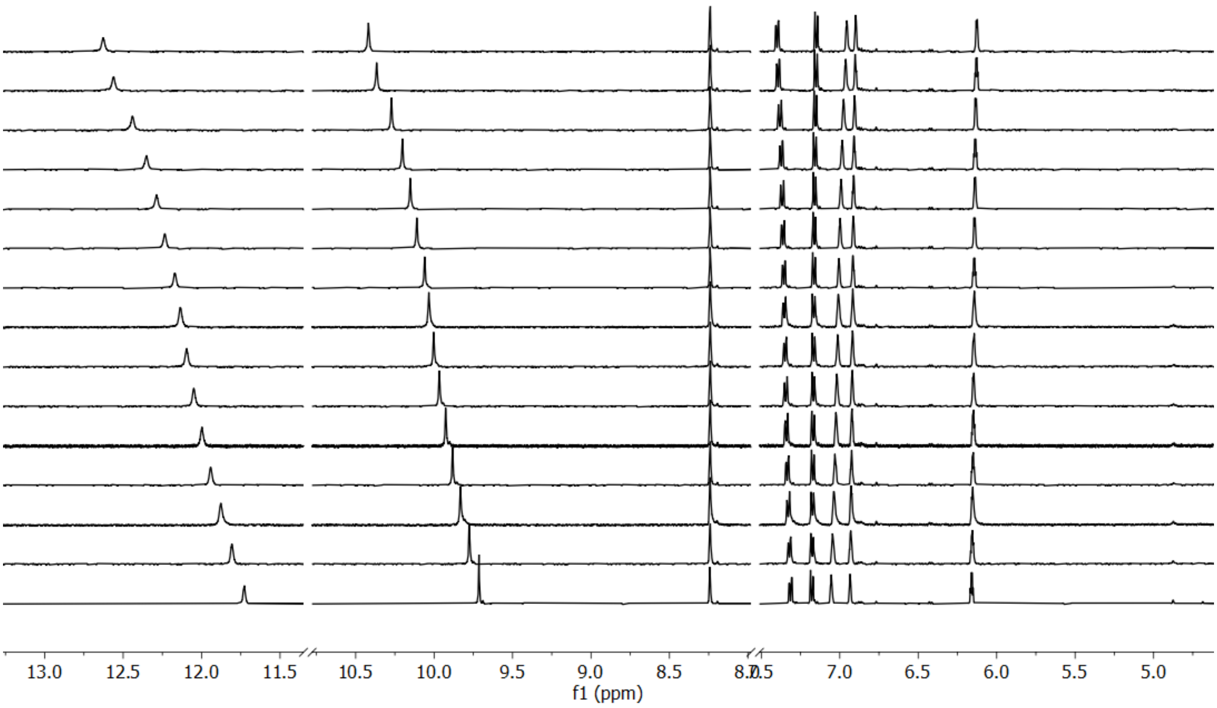

Figure S31. ^{1}H NMR spectral changes (500 MHz, 293 K) of (*E*)-**1** in DMSO-d_6/0.5%H_2O (2.8 mM) upon the stepwise addition of $[Bu_4N]^+[CH_3COO]^-$ (from bottom to top: 0.00, 0.25, 0.47, 0.69, 0.91, 1.13, 1.34, 1.53, 1.71, 2.07, 2.40, 2.87, 3.56, 4.65, 5.53 equivalents).

Addition of $[Bu_4N]^+[CH_3COO]^-$ to (*E*)-2:

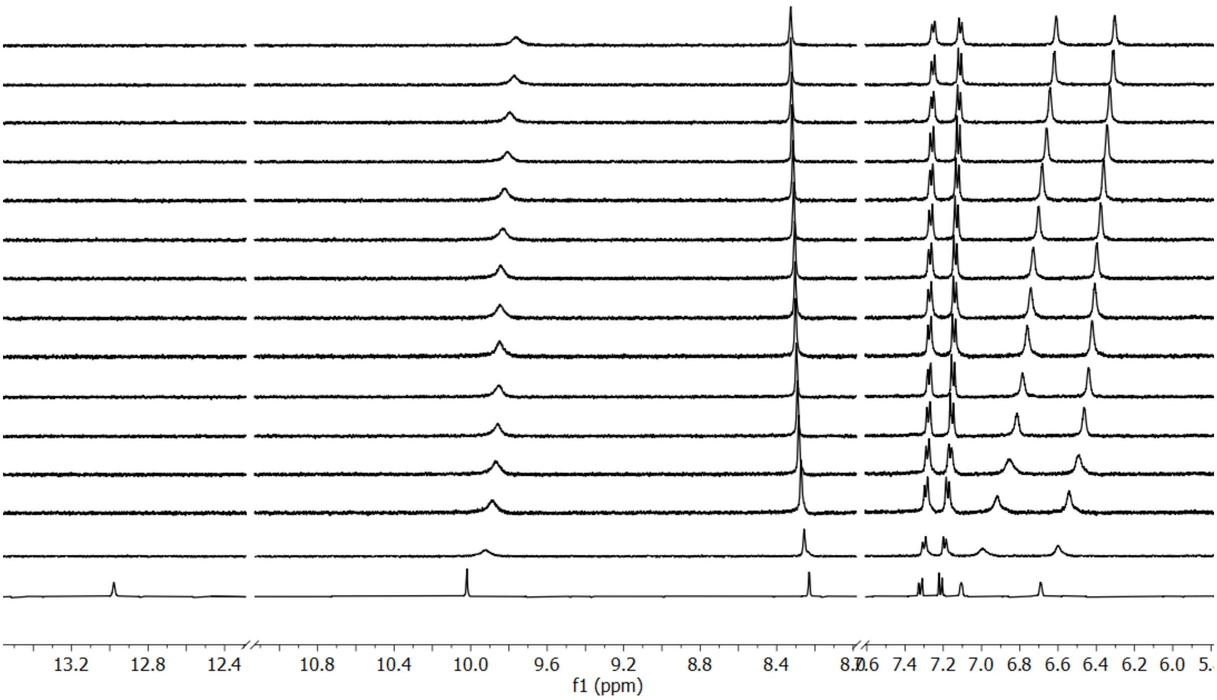

Figure S32. ^{1}H NMR spectral changes (500 MHz, 293 K) of (*E*)-**2** in DMSO-d_6/0.5%H_2O (2.1 mM) upon the stepwise addition of $[Bu_4N]^+[CH_3COO]^-$ (from bottom to top: 0.00, 0.35, 0.68, 1.00, 1.31, 1.61, 1.90, 2.18, 2.45, 2.96, 3.43, 4.09, 5.07, 6.65, 7.89 equivalents).

Addition of $[Bu_4N]^+[Cl]^-$ to (*E*)-1:

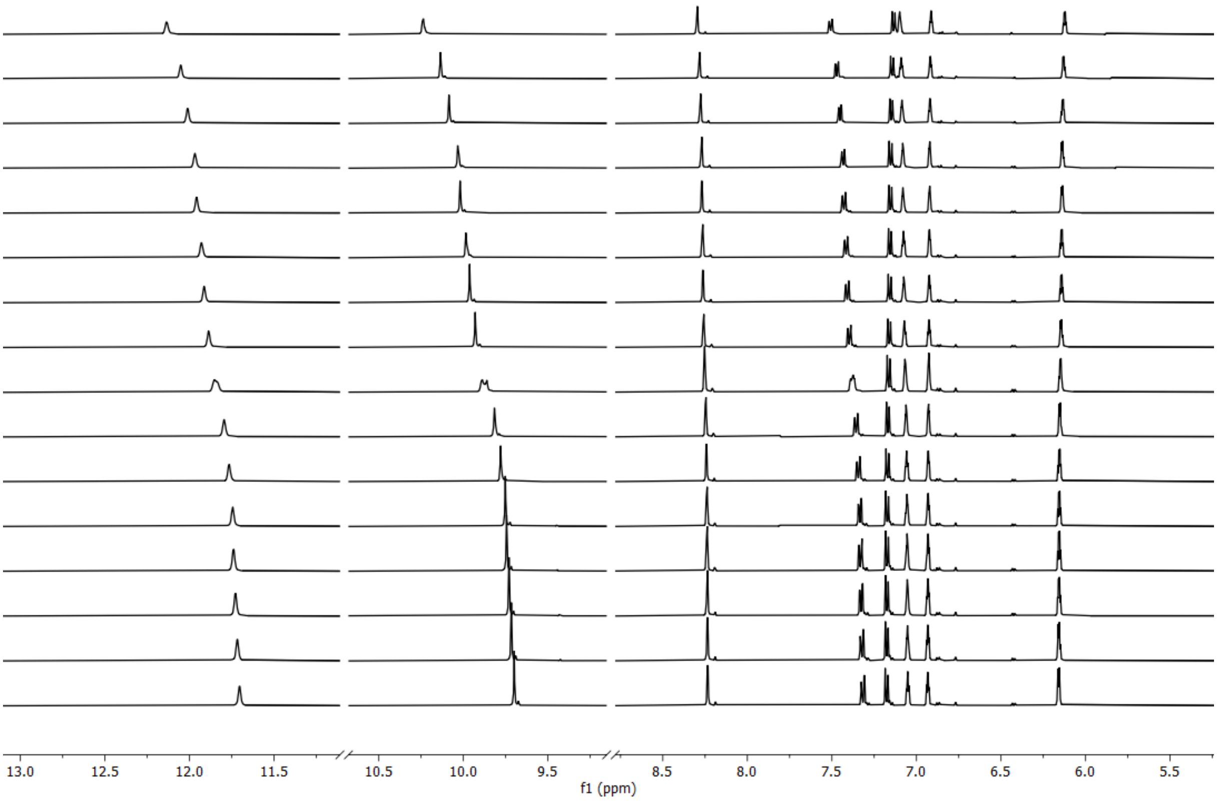

Figure S33. ^{1}H NMR spectral changes (500 MHz, 293 K) of (*E*)-**1** in DMSO-d_6/0.5%H_2O (5.0 mM) upon the stepwise addition of $[Bu_4N]^+[Cl]^-$ (from bottom to top: 0.00, 0.69, 1.33, 1.93, 2.48, 5.49, 8.40, 13.9, 16.6, 19.1, 21.6, 24.0, 26.3, 28.6, 32.9, 48.0 equivalents).

Addition of $[Bu_4N]^+[Cl]^-$ to (*E*)-2:

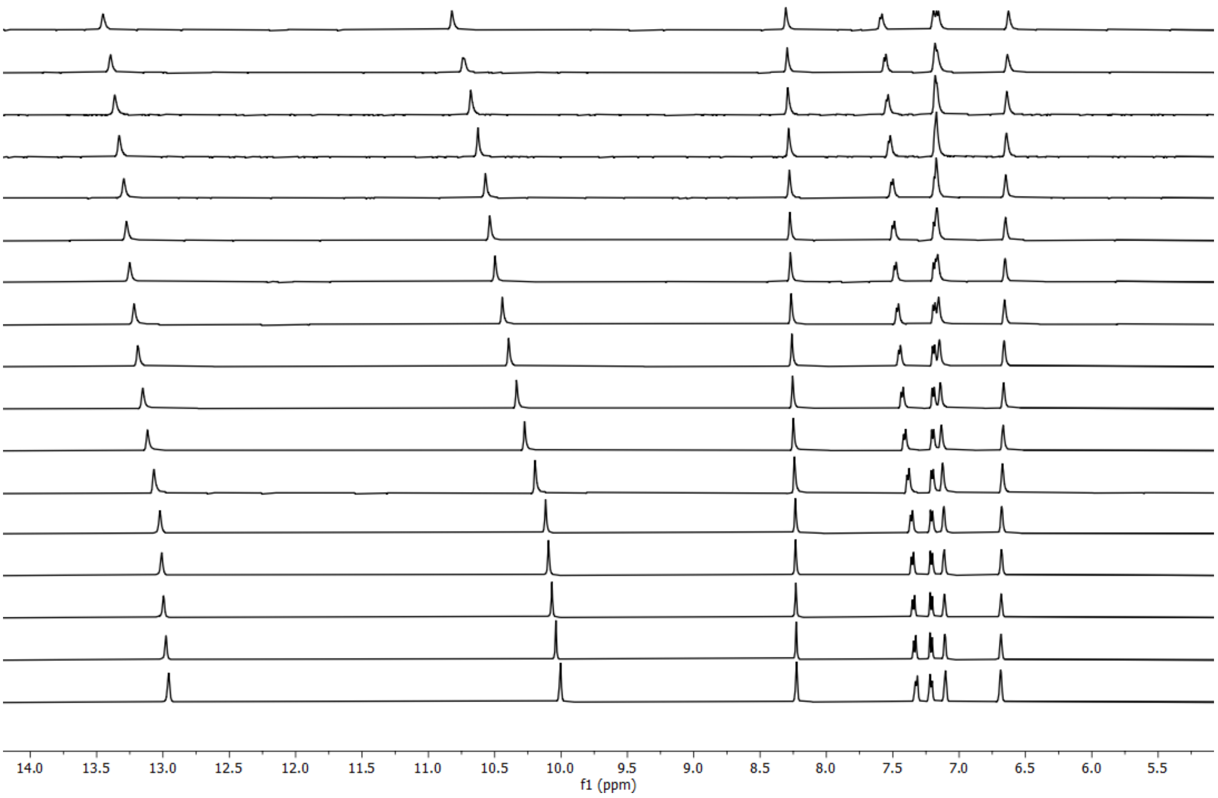

Figure S34. ^{1}H NMR spectral changes (500 MHz, 293 K) of (*E*)-**2** in DMSO-d_6/0.5%H_2O (5.0 mM) upon the stepwise addition of $[Bu_4N]^+[Cl]^-$ (from bottom to top: 0.00, 0.69, 1.33, 1.93, 2.49, 5.49, 8.40, 11.2, 13.9, 16.6, 19.1, 21.6, 24.0, 28.6, 32.9, 39.0, 48.0 equivalents).

^{1}H NMR titration data fitting

Data fitting was performed in the same way as previously described for stiff-stilbene based bis-(thio)urea receptors.[1] That is, as no clear distinction could be made between the ^{1}H NMR chemical shifts of the 1:1 and 1:2 complexes throughout the titration the two amidopyrrole binding sites were treated as equal, i.e. $K_{1m} = K_{2m}$ (cooperativity factor $\alpha = 1$). Furthermore, the data was initially fitted to a 1:1 model to afford K_{11}, after which the stability constant of the 1:2 complex [$K_{21} = (K_{11}/2)^2$] was included. All data fits were performed with HypNMR.[2]

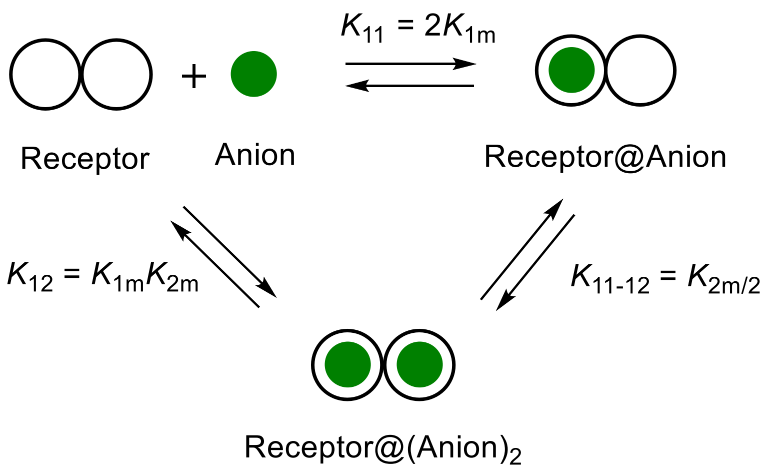

Figure S35. Schematic representation of the equilibria and species involved in the stepwise formation of a 1:2 (receptor/anion) complex. The relation of the stability constants of the 1:1 complex (K_{11}), the 1:2 complex (K_{12}), and the stepwise constant ($K_{11\text{-}12}$) to the microscopic binding constants (K_{1m}, K_{2m}) is additionally shown.

Addition of $[Bu_4N]^+[CH_3COO]^-$ to (*Z*)-1:

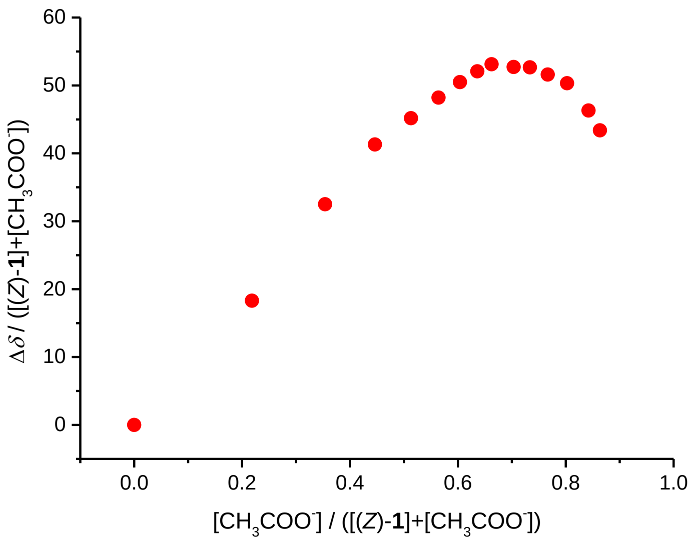

Figure S36. Modified Job plot analysis generated from the addition of $[Bu_4N]^+[CH_3COO]^-$ to (*Z*)-**1** (using the pyrrole-NH chemical shift values) indicating a 1:2 binding stoichiometry.

Addition of $[Bu_4N]^+[CH_3COO]^-$ to (*E*)-1:

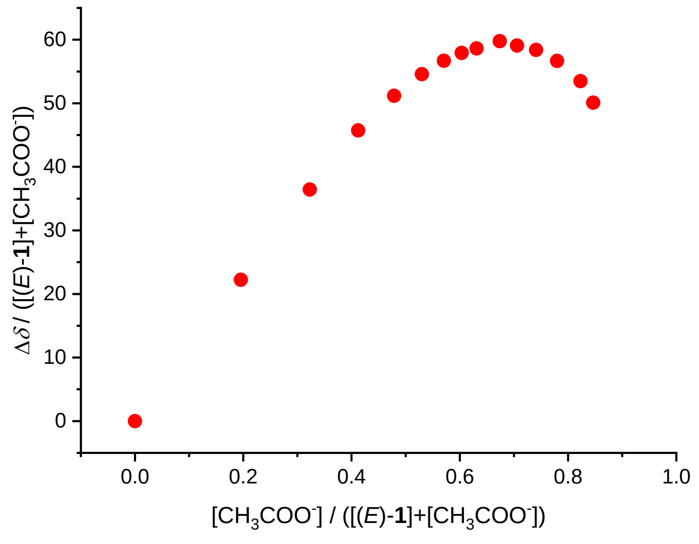

Figure S37. Modified Job plot analysis generated from the addition of $[Bu_4N]^+[CH_3COO]^-$ to (*E*)-**1** (using the pyrrole-NH chemical shift values) indicating a 1:2 binding stoichiometry.

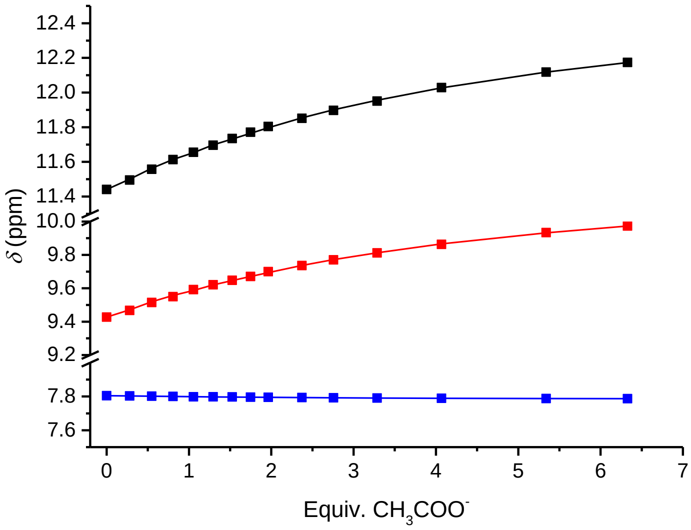

Figure S38. Titration curve for the addition of $[Bu_4N]^+[CH_3COO]^-$ to (*Z*)-**1** and data fit obtained by simultaneous analysis of pyrrole-NH (black), amide-NH (red) and aromatic-H (blue) ^{1}H NMR signals using a 1:2 binding model: K_{11} = 98 M^{-1}; K_{1m} = 49 M^{-1}.

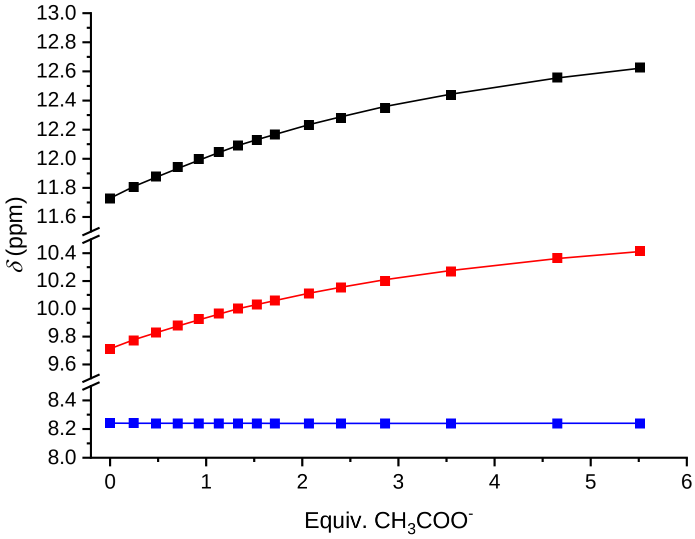

Figure S39. Titration curve for the addition of $[Bu_4N]^+[CH_3COO]^-$ to (*E*)-**1** and data fit obtained by simultaneous analysis of pyrrole-NH (black), amide-NH (red) and aromatic-H (blue) ^{1}H NMR signals using a 1:2 binding model: K_{11} = 102 M^{-1}; K_{1m} = 51 M^{-1}.

Addition of $[Bu_4N]^+[Cl]^-$ to (*Z*)-1:

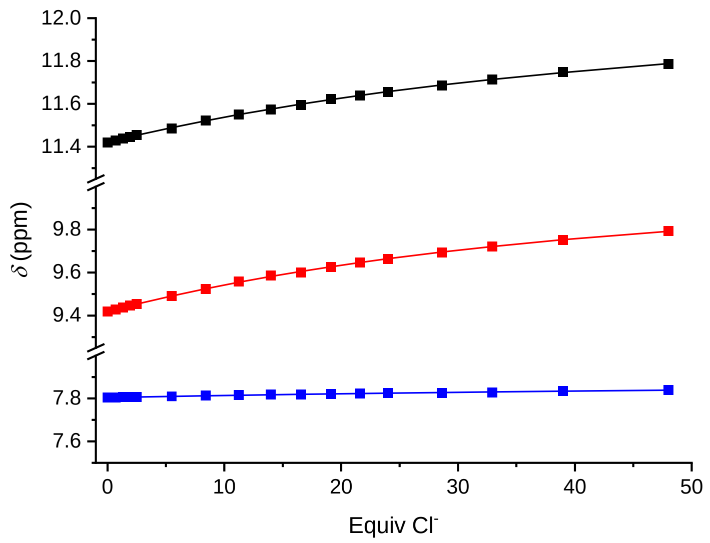

Figure S40. Titration curve for the addition of $[Bu_4N]^+[Cl]^-$ to (*Z*)-**1** and data fit obtained by simultaneous analysis of pyrrole-NH (black), amide-NH (red) and aromatic-H (blue) ^{1}H NMR signals using a 1:2 binding model: K_{11} = 3.5 M^{-1}; K_{1m} = 1.8 M^{-1}.

Addition of $[Bu_4N]^+[Cl]^-$ to (*Z*)-2:

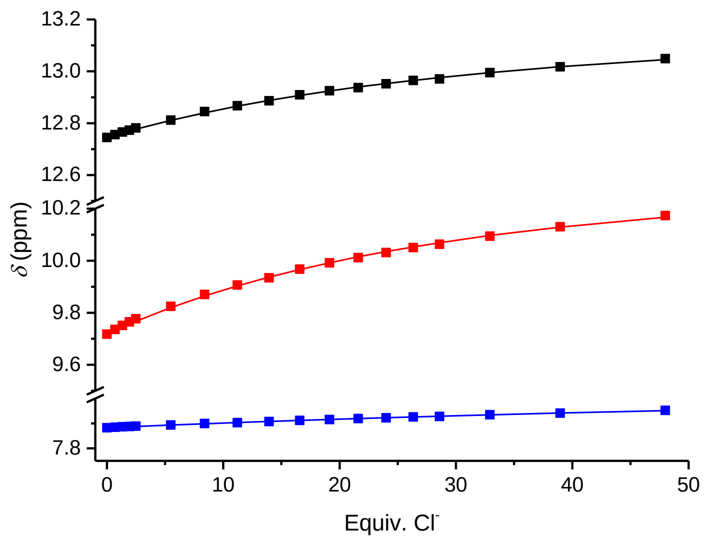

Figure S41. Titration curve for the addition of $[Bu_4N]^+[Cl]^-$ to (*Z*)-**2** and data fit obtained by simultaneous analysis of pyrrole-NH (black), amide-NH (red) and aromatic-H (blue) ^{1}H NMR signals using a 1:2 binding model: K_{11} = 5.5 M^{-1}; K_{1m} = 2.8 M^{-1}.

Addition of $[Bu_4N]^+[Cl]^-$ to (*E*)-1:

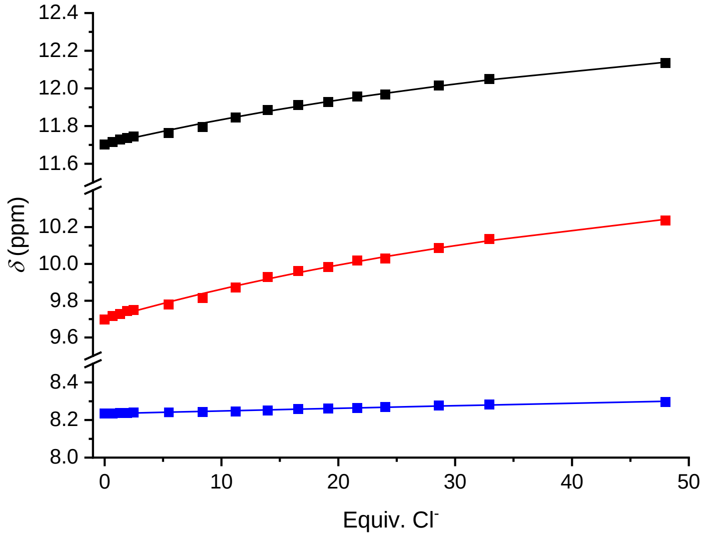

Figure S42. Titration curve for the addition of $[Bu_4N]^+[Cl]^-$ to (*E*)-**1** and data fit obtained by simultaneous analysis of pyrrole-NH (black), amide-NH (red) and aromatic-H (blue) ^{1}H NMR signals using a 1:2 binding model: K_{11} = 2.7 M^{-1}; K_{1m} = 1.3 M^{-1}.

Addition of $[Bu_4N]^+[Cl]^-$ to (*E*)-2:

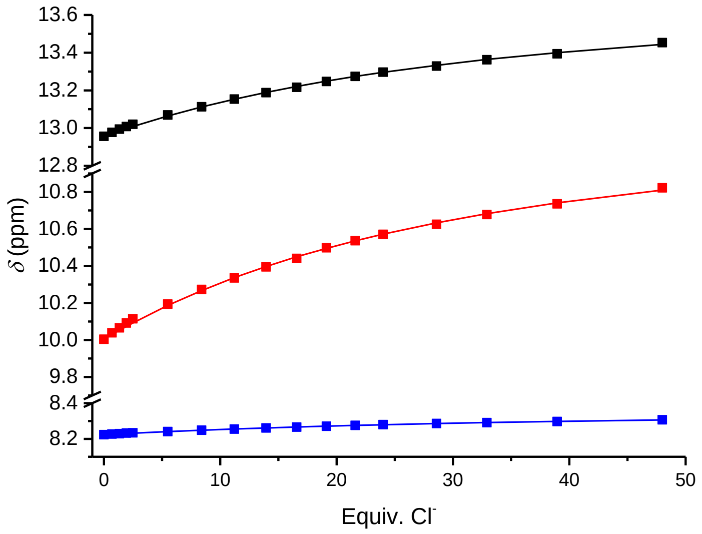

Figure S43. Titration curve for the addition of $[Bu_4N]^+[Cl]^-$ to (*E*)-**2** and data fit obtained by simultaneous analysis of pyrrole-NH (black), amide-NH (red) and aromatic-H (blue) ^{1}H NMR signals using a 1:2 binding model: K_{11} = 5.6 M^{-1}; K_{1m} = 2.8 M^{-1}.

Transmembrane transport experiments

Vesicle preparation – HPTS assay

HPTS transport assays were performed using POPC vesicles loaded with the pH-responsive fluorescent dye HPTS. A 10 mM POPC solution in $CHCl_3$ was prepared in a round-bottom flask and the solvent was evaporated to create a lipid film which was dried under vacuum for at least 12 h. The lipid film was hydrated by vortexing with the internal solution, containing HPTS (1 mM) and NaCl (100 mM) buffered to pH 7.0 with HEPES (10 mM). Subsequently, the suspension was subjected to 9 freeze and thaw cycles, by freezing in a liquid nitrogen bath followed by thawing in a water bath at 45°C. After standing at rt for 30 min, the suspension was extruded 25 times through a 200 nm polycarbonate membrane to obtain unilamellar vesicles. Unencapsulated dye was removed with an Illustra NAP™-10 Sephadex® G-25 column using the external solution containing NaCl (100 mM) buffered to pH 7.0 with HEPES (10 mM), affording a stock solution with a concentration of 6.67 mM in lipid.

Vesicles with transporter pre-incorporated were prepared from a stock solution of 10 mM POPC and 1 mM transporter (10 mol%) in $CHCl_3$, which was diluted with a 10 mM POPC solution to the appropriate molar ratio. Using the above protocol, with the exception that now an Illustra NAP™-25 Sephadex® G-25 columns was used, stock solutions with a concentration of 1.6 mM in lipid and with various concentrations of the transporter were obtained.

For each measurement, the stock solution was diluted with buffer to a standard volume (2.5 mL) in a PS cuvette to obtain a solution with a concentration of 0.1 mM in lipid. The sample was stirred at rt in the fluorimeter, and the fluorescence ratio of HPTS (λ_{ex} = 454 nm, λ_{em} = 511 nm, base form, divided by λ_{ex} = 403 nm, λ_{em} = 511 nm, acid form) was measured over time. At t = 30 s, the compounds were added as a DMSO solution (5.0 µL, varying concentrations by dilution from a 5 mM stock solution). To initiate transport, a pulse of NaOH (25 µL, 0.5 M) was given at t = 60s to generate a pH gradient of pH 7 inside and pH 8 outside. Vesicles were lysed with Triton X-100 (50 µL, 11 wt% in H_2O/DMSO 7:1 *v*/*v*) at t = 370s, and a final reading was taken at t = 430s. The fractional fluorescent intensity (I_f) was then calculated using the following formula:

$$I_f = \frac{R_t - R_0}{R_d - R_0} \quad (\mathbf{S1})$$

Where R_t is the fluorescent ratio at time t, R_0 is the initial fluorescent ratio measured just prior to the base-pulse at t = 59s, and R_d is the final ratio measured at t = 430s.

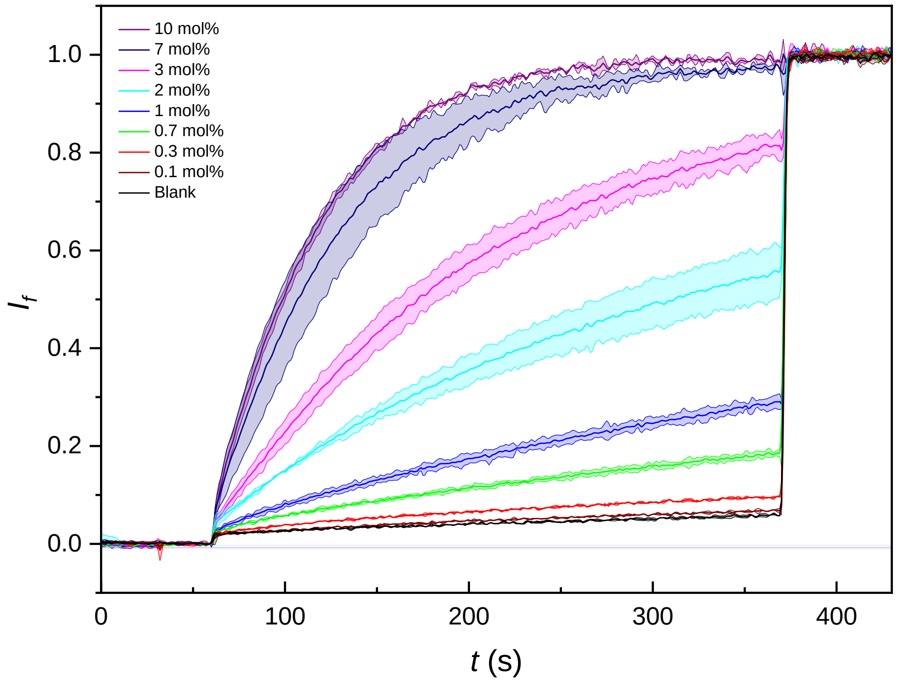

Figure S44. Plots of H^+/Cl^- symport (or Cl^-/OH^- antiport) against time across a POPC membrane facilitated by (*Z*)-**1** after post-addition from a DMSO solution (each measurement done in duplicate).

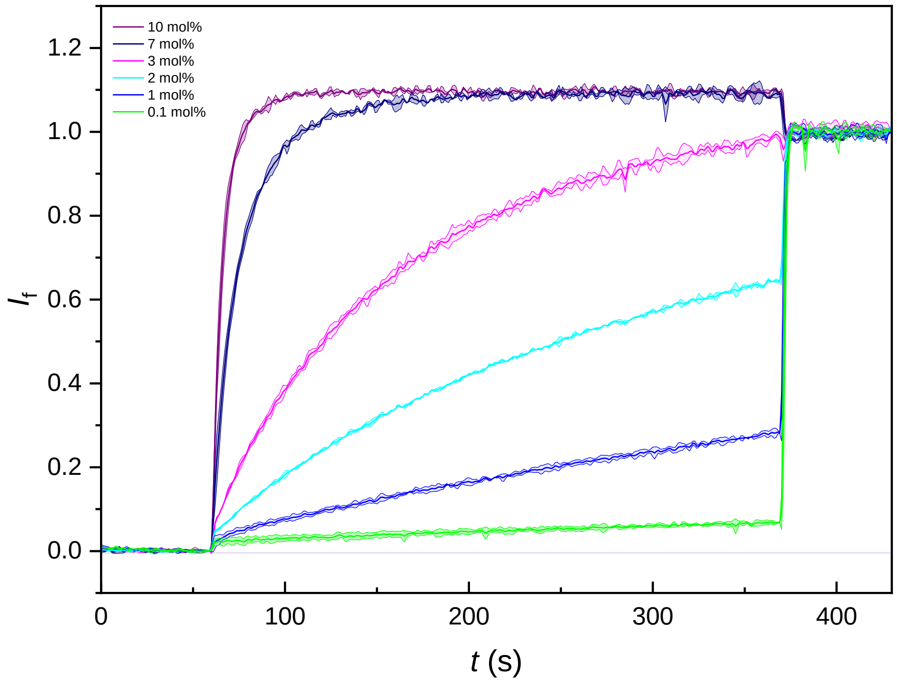

Figure S45. Plots of H^+/Cl^- symport (or Cl^-/OH^- antiport) against time across a POPC membrane facilitated by (*Z*)-**1** that is pre-incorporated in the membrane (each measurement done in duplicate).

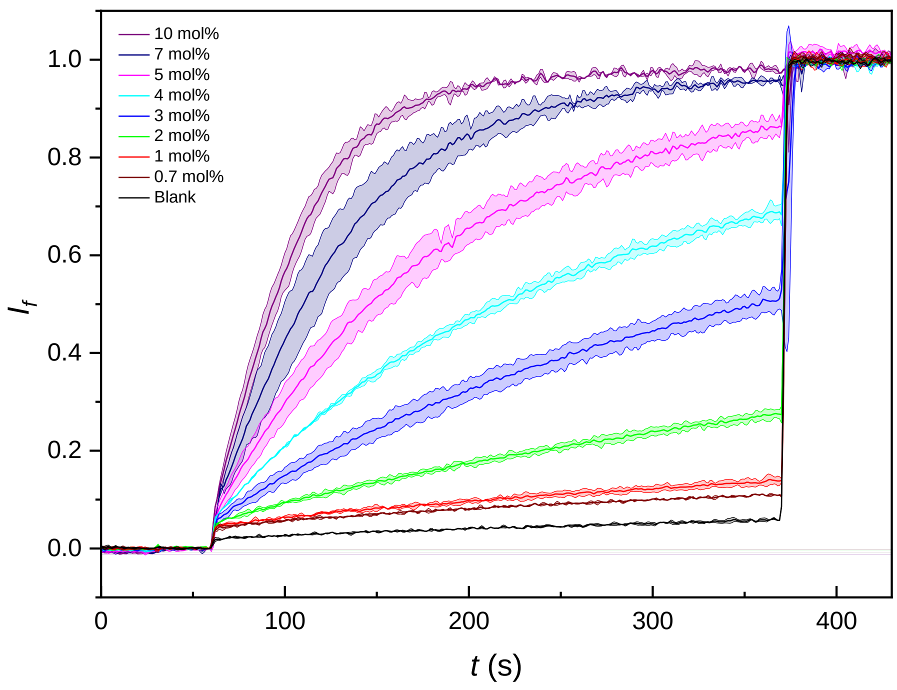

Figure S46. Plots of H^+/Cl^- symport (or Cl^-/OH^- antiport) against time across a POPC membrane facilitated by (*E*)-**1** after post-addition from a DMSO solution (each measurement done in duplicate).

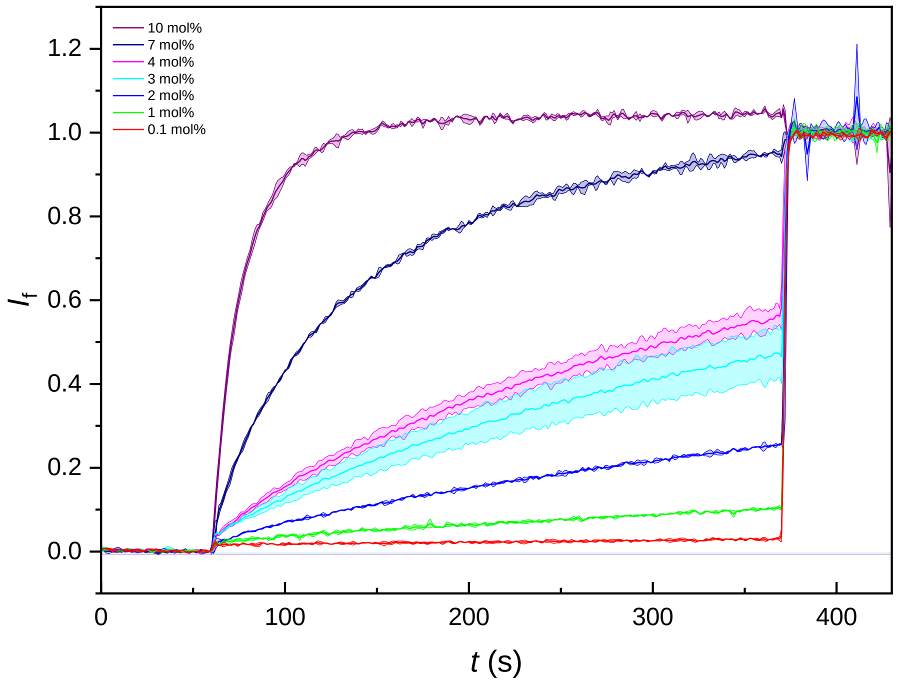

Figure S47. Plots of H^+/Cl^- symport (or Cl^-/OH^- antiport) against time across a POPC membrane facilitated by (*E*)-**1** that is pre-incorporated in the membrane (each measurement done in duplicate).

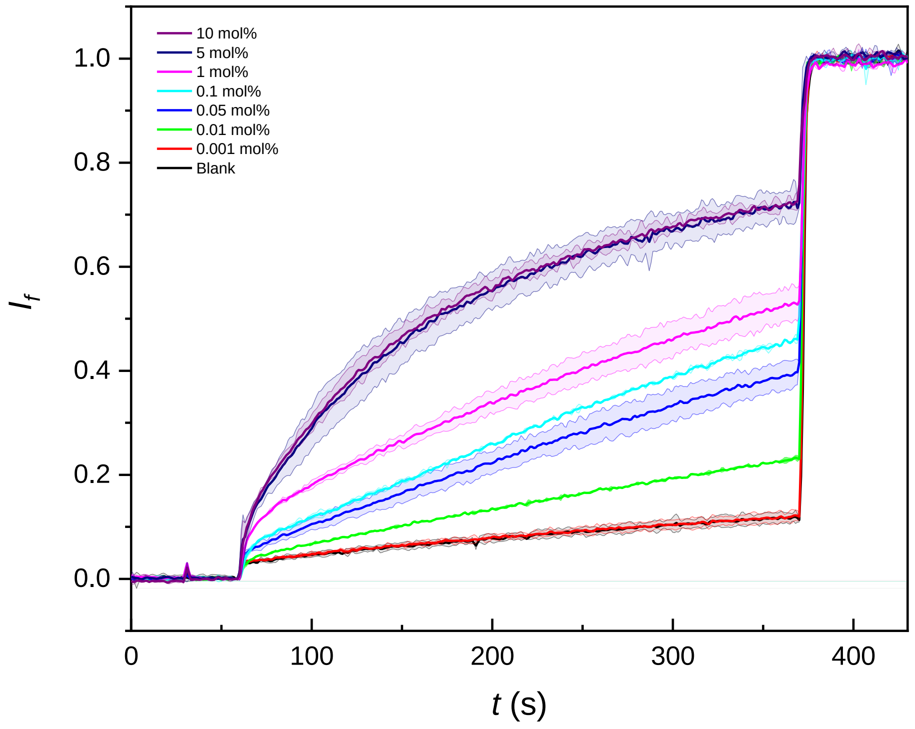

Figure S48. Plots of H^+/Cl^- symport (or Cl^-/OH^- antiport) against time across a POPC membrane facilitated by (*Z*)-**2** after post-addition from a DMSO solution (each measurement done in duplicate).

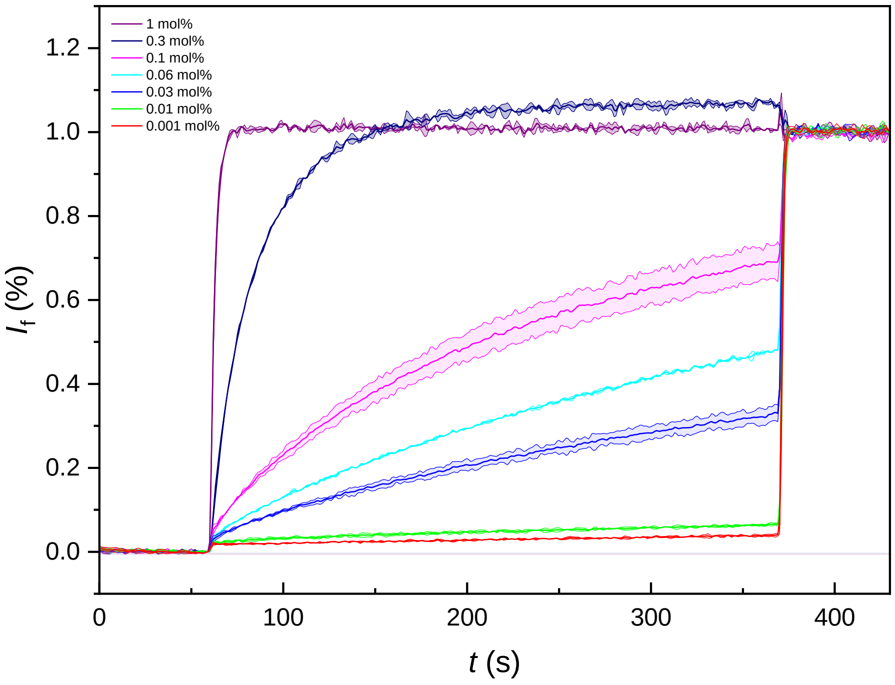

Figure S49. Plots of H^+/Cl^- symport (or Cl^-/OH^- antiport) against time across a POPC membrane facilitated by (*Z*)-**2** that is pre-incorporated in the membrane (each measurement done in duplicate).

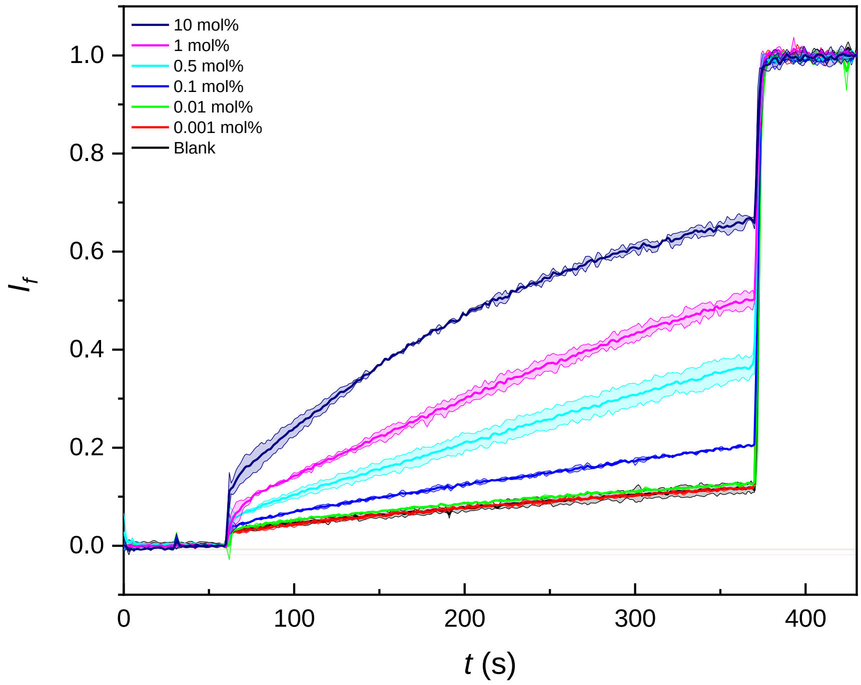

Figure S50. Plots of H^+/Cl^- symport (or Cl^-/OH^- antiport) against time across a POPC membrane facilitated by (*E*)-**2** after post-addition from a DMSO solution (each measurement done in duplicate).

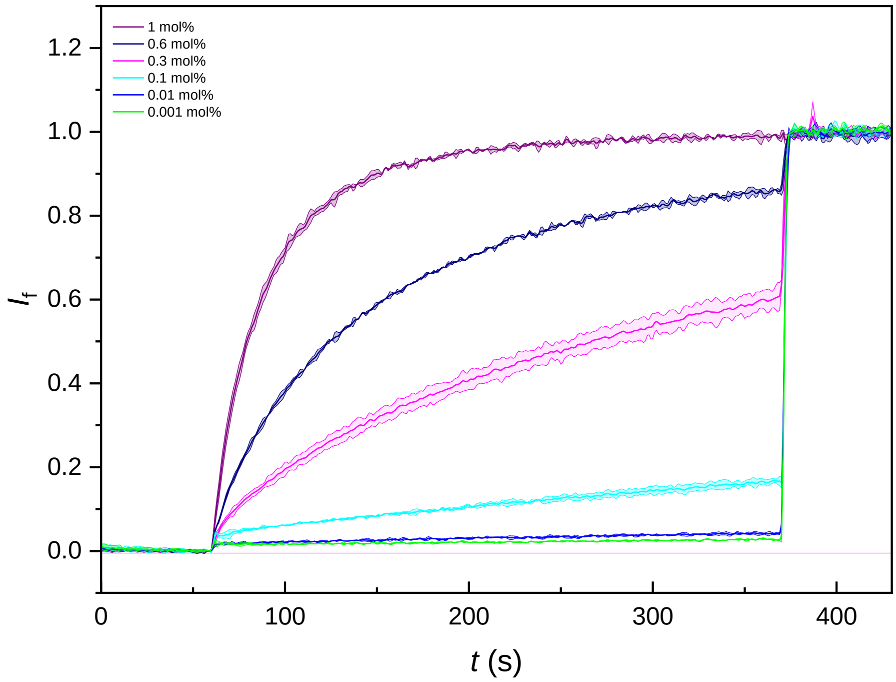

Figure S51. Plots of H^+/Cl^- symport (or Cl^-/OH^- antiport) against time across a POPC membrane facilitated by (*E*)-**2** that is pre-incorporated in the membrane (each measurement done in duplicate).

Hill analysis

The transport activity measured at t = 360s was plotted as a function of transporter concentration. Using Origin 2022 this data was fitted to the Hill equation:

$$y = y_0 + (y_{max} - y_0)\frac{x^n}{k^n + x^n} \quad (\mathbf{S2})$$

Where y is the I_f at t = 360s and x is the transporter concentration (in mol% with respect to lipid). The parameters to be fitted are: y_0 which is the I_f when no transporter is added, y_{max} is the maximum I_f that is obtained by the receptor, k is the concentration needed to get 50% of this observed maximum I_f and n is the Hill coefficient.

The EC_{50} value is defined as the concentration needed to reach 50% of the maximum possible chloride efflux. The value of k will only correspond to EC_{50} if the receptor is able to induce 100% of efflux. In the case that 100% efflux is not reached even at the highest loadings, *e.g.* due to poor deliverability (see Figures S48 and S50), the calculated value of k would instead reflect the concentration where half of the maximum chloride efflux with that receptor can be reached and not where 50% of the chloride is effluxed. To determine the correct EC_{50} value, we therefore derived the following equation:

$$EC_{50} = k\left(\frac{0.5 - 0.5y_0}{y_{max} - 0.5 - 0.5y_0}\right)^{1/n} \quad (\mathbf{S3})$$

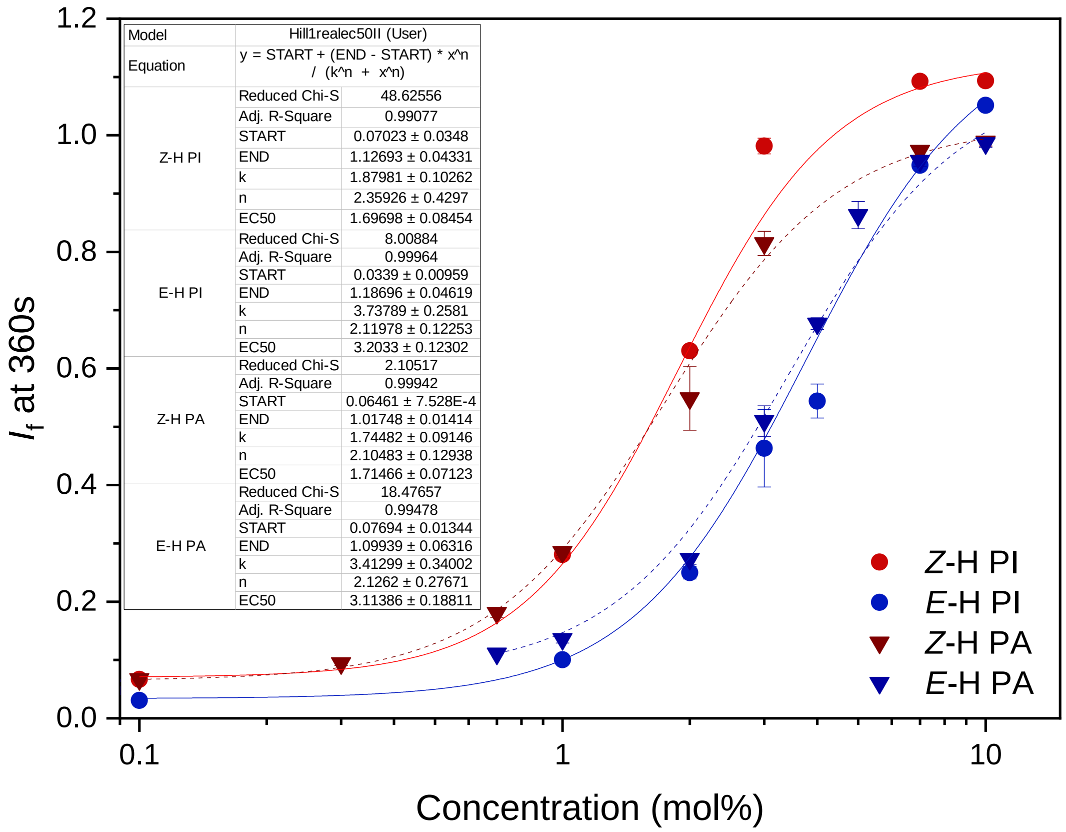

Figure S52. Hill plot of H^+/Cl^- symport (or Cl^-/OH^- antiport) facilitated by (*Z*)-**1** (red) and (*E*)-**1** (blue) in the HPTS assay, comparing the traces from pre-incorporated (solid line with circles) and post-added (dashed line with triangles) experiments.

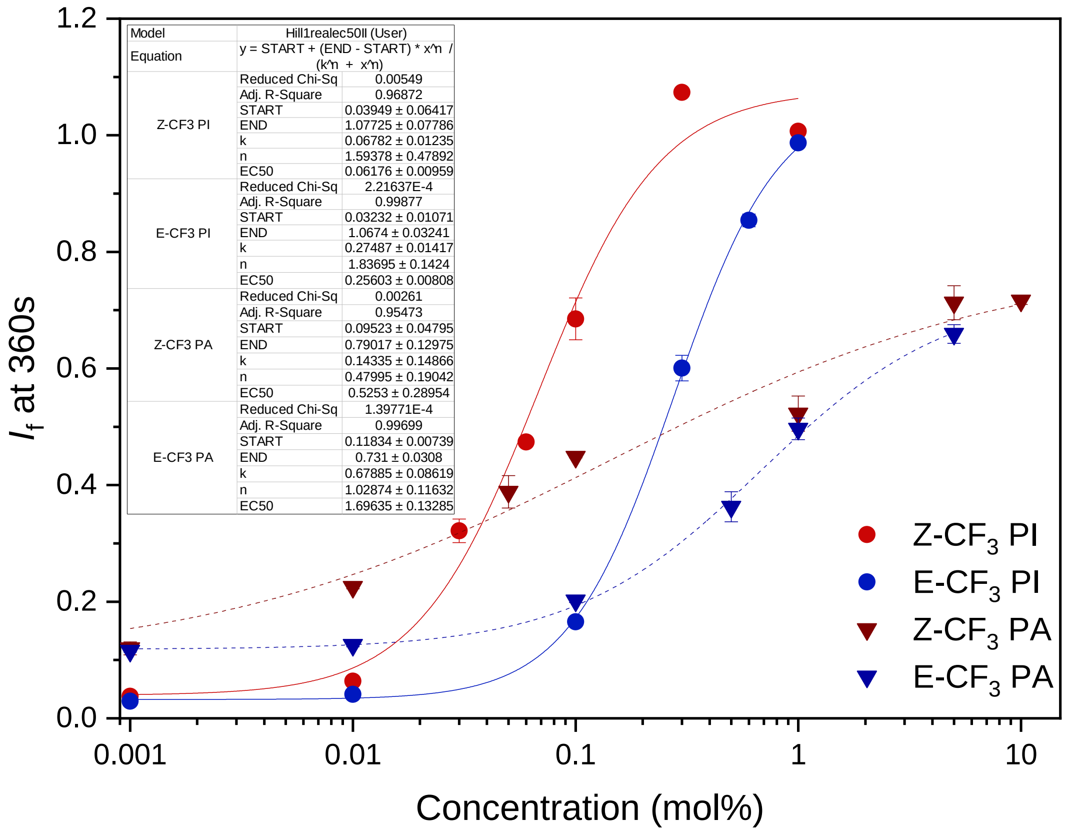

Figure S53. Hill plot of H^+/Cl^- symport (or Cl^-/OH^- antiport) facilitated by (*Z*)-**2** (red) and (*E*)-**2** (blue) in the HPTS assay, comparing the traces from pre-incorporated (solid line with circles) and post-added (dashed line with triangles) experiments.

***In situ* irradiation**

A vesicle solution (0.1 mᴍ in lipids, 2.5 mL) with 0.1 mol% of compound (*E*)-**2** pre-incorporated, was prepared as stated in the above protocol for the HPTS assay. The fluorescence ratio of HPTS was followed over time, and subsequently converted to the fractional fluorescent intensity (I_f) using equation **S1**. At t = 60s, the NaOH base-pulse (25 µL, 0.5 ᴍ) was added to initiate transport. During the experiment samples were irradiated between t = 120s – 150s using a 340 nm or 365 nm LED mounted on the lid of the fluorimeter (see Figure S54). At t = 370s the vesicles were lysed with Triton X-100 (50 µL, 11 wt% in H_2O/DMSO 7:1 *v*/*v*), and a final reading was taken at t = 430 s. The traces of the irradiated samples were compared to traces that were not irradiated.

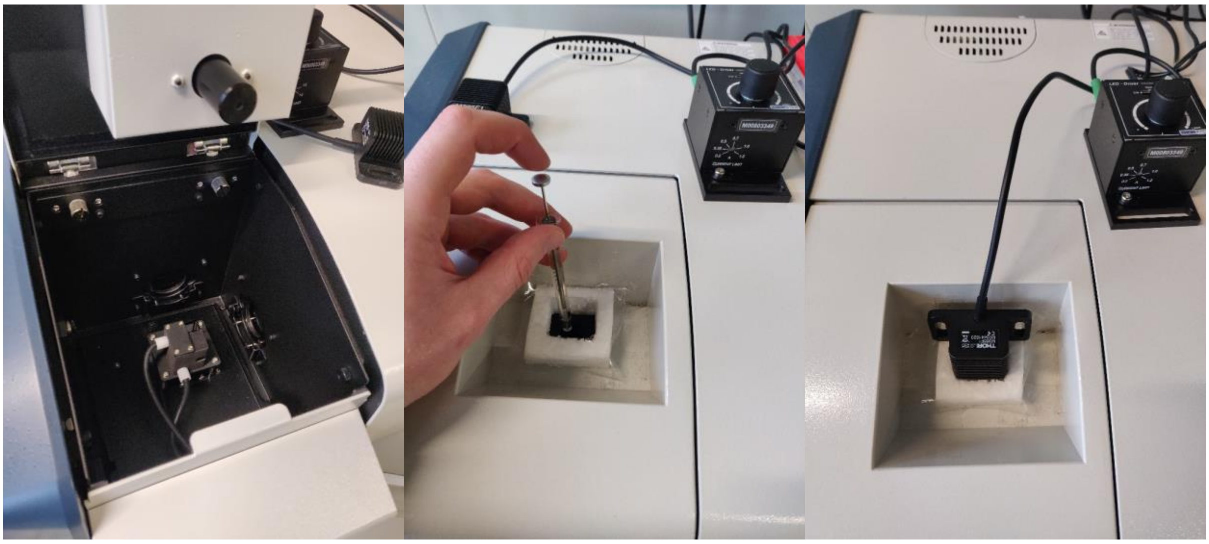

Figure S54. Experimental setup for irradiation inside the fluorimeter during the HPTS assay. Showing the inside of the fluorimeter (left), addition of the solutions through the lid (middle) and mounting of the LED for irradiation of the sample (right).

Cationophore coupled assay

For the cationophore-coupled assays a solution of 37.5 mM POPC and 0.375 mM transporter (1 mol%) in $CHCl_3$ was prepared in a round bottom flask. The solvent was evaporated to create a lipid film which was dried under vacuum for at least 12 h. The film was hydrated by vortexing with the internal solution consisting of KCl (300 mM) buffered to pH 7.20 with HEPES (10 mM). Next, the suspension was subjected to 9 freeze and thaw cycles, using liquid nitrogen and a water bath at 45°C. The solution was left standing at rt for 30 min, and subsequently extruded 25 times through a 200 nm polycarbonate membrane to obtain unilamellar vesicles. The external solution was exchanged for one containing KGlu (300 mM) buffered to pH 7.20 with HEPES (10 mM) using an Illustra NAP™-10 Sephadex® G-25 column, which afforded stock solutions with a concentration between 15-18 mM in lipid.

During the assays the chloride concentration was measured using an Accumet chloride-selective electrode. For each measurement, the stock solution was diluted with buffer to a standard volume (5.0 mL) in a glass vial to obtain a solution with a concentration of 1.0 mM in lipid. The sample was stirred at rt and the potential was measured over time using the electrode. At t = 60s the cationophore (valinomycin or monensin) was added as a DMSO solution (10 µL, 0.5 mM, 0.1 mol%) to initiate transport. Vesicles were lysed with Triton X-100 (50 µL, 11 wt% in H_2O/DMSO 7:1 *v*/*v*) at t = 360s and a final reading corresponding to 100% efflux was taken at t = 480s.

The electrode was calibrated prior to each experiment according to the supplier's manual, by recording the potential of solutions with a known chloride concentration. A calibration curve was generated by fitting to the modified Nernst equation (Equation S4).

$$y = a + b \ln x \quad (\mathbf{S4})$$

Where y is the potential (mV), x is the chloride concentration and a and b are the parameters to be fitted. Using the calibration curve, readings (mV) of the experiment were converted to chloride concentrations, and subsequently to percentages of efflux using equation S5.

$$Efflux = \frac{C_t - C_0}{C_d - C_0} \times 100\% \ (\mathbf{S5})$$

Where C_t is the concentration at time t, C_0 is the initial concentration measured just prior to addition of the cationophore at t = 57s, and C_d is the final ratio measured at t = 480s.

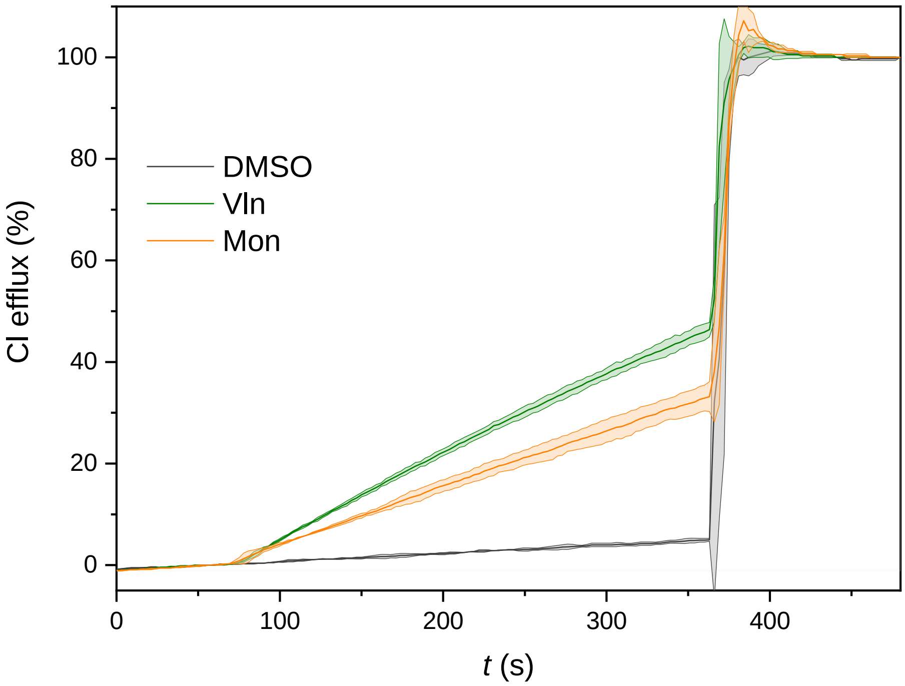

Figure S55. Electrogenic or electroneutral transport facilitated by (*Z*)-**1** (1 mol%) that is pre-incorporated in POPC vesicles, in the presence of valinomycin or monensin (0.1 mol%). DMSO was used as control (each measurement done in duplicate).

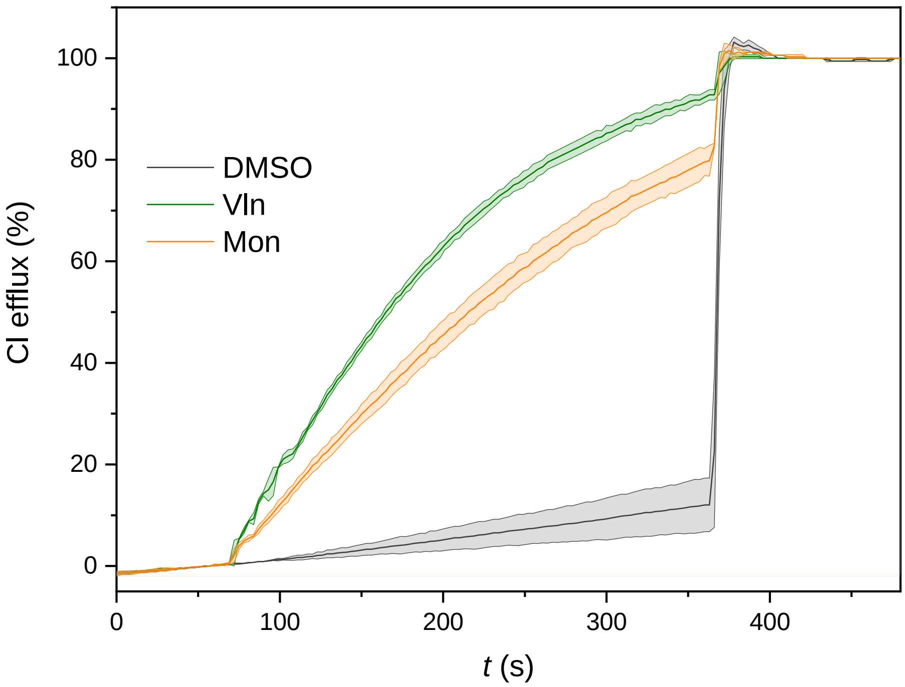

Figure S56. Electrogenic or electroneutral transport facilitated by (*Z*)-**2** (1 mol%) that is pre-incorporated in POPC vesicles, in the presence of valinomycin or monensin (0.1 mol%). DMSO was used as control (each measurement done in duplicate).

Geometry optimization by DFT

Input geometries were generated using ArgusLab.[3] The Gaussian 09 program[4] was used for geometry optimization: First, energy minimization was performed at the semi-empirical PM3 level of theory, and subsequently at the DFT B3LYP/6-31++G(d,p) level of theory using an IEF-PCM DMSO solvation model. The DFT optimized geometries were found to have zero imaginary frequencies.

Table S1. Cartesian coordinates of (*Z*)-**1**⊂Cl⁻.

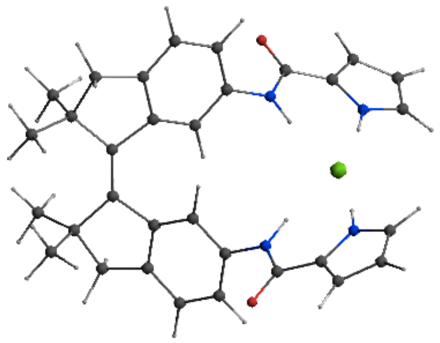

Atom	X	Y	Z
C	3.1818960000	0.4846680000	-0.4820830000
C	3.1818980000	-0.4846460000	0.4820880000
C	1.9735060000	-1.2296100000	0.9414160000
C	2.2154310000	-1.8291730000	2.1894360000
C	3.5836720000	-1.4506190000	2.6700840000
C	4.3460790000	-1.0435680000	1.3622600000
C	1.9735010000	1.2296160000	-0.9414250000
C	2.2154290000	1.8291610000	-2.1894540000
C	3.5836780000	1.4506130000	-2.6700860000
C	4.3460790000	1.0435910000	-1.3622520000
C	0.7892760000	-1.5418100000	0.2534970000
C	-0.1510960000	-2.3914040000	0.8441770000
C	0.0834620000	-2.9484400000	2.1115580000
C	1.2784710000	-2.6765090000	2.7799410000
C	0.7892580000	1.5418080000	-0.2535230000
C	-0.1511220000	2.3913750000	-0.8442300000
C	0.0834420000	2.9483940000	-2.1116170000
C	1.2784630000	2.6764750000	-2.7799820000
C	-1.7719660000	3.9242490000	0.2095960000
O	-1.0505530000	4.9229990000	0.0372590000
C	-1.7719860000	-3.9242740000	-0.2095960000
O	-1.0505790000	-4.9230370000	-0.0373000000
N	-1.3733250000	2.6662140000	-0.1554150000

N	-1.3732820000	-2.6662360000	0.1553260000
Cl	-3.4464430000	-0.0000530000	0.0002070000
N	-4.0316980000	3.0564370000	1.0024460000
C	-5.1429650000	3.5571300000	1.6147460000
C	-4.9402100000	4.9104920000	1.8554810000
C	-3.6524610000	5.2299980000	1.3622010000
C	-3.0992240000	4.0652390000	0.8338020000
N	-4.0317020000	-3.0564120000	-1.0024220000
C	-5.1428770000	-3.5570160000	-1.6149650000
C	-4.9401910000	-4.9104010000	-1.8556250000
C	-3.6525750000	-5.2300110000	-1.3620650000
C	-3.0992380000	-4.0652300000	-0.8338220000
H	-0.6612570000	-3.6017510000	2.5532470000
H	-0.6612870000	3.6016840000	-2.5533230000
H	4.0980380000	-2.2550620000	3.2066860000
H	3.5289860000	-0.5885840000	3.3498130000
H	4.0980380000	2.2550500000	-3.2067000000
H	3.5290040000	0.5885650000	-3.3498010000
H	-1.9676630000	1.8602330000	0.0366070000
H	-1.9676860000	-1.8602670000	-0.0365530000
H	0.5929520000	-1.1522130000	-0.7389860000
H	1.4764730000	-3.1304630000	3.7473350000
H	0.5929300000	1.1522260000	0.7389650000
H	1.4764720000	3.1304190000	-3.7473800000
H	-3.9354920000	2.0832250000	0.7042690000
H	-5.9900160000	2.9214570000	1.8294060000
H	-3.1629420000	6.1930290000	1.3812090000
H	-5.6417110000	5.5822290000	2.3306090000
H	-3.9355180000	-2.0832310000	-0.7041340000
H	-5.9898480000	-2.9212840000	-1.8297690000
H	-3.1631400000	-6.1930870000	-1.3809380000
H	-5.6416700000	-5.5820960000	-2.3308460000
C	4.9534140000	-2.3311880000	0.7517450000
C	5.4585750000	0.0592630000	-1.7552220000
C	5.4585690000	-0.0592460000	1.7552580000
C	4.9534000000	2.3312270000	-0.7517580000
H	4.1784080000	-3.0831220000	0.5697280000
H	5.6848630000	-2.7614810000	1.4458220000
H	5.4602130000	-2.1352510000	-0.1943190000
H	5.0390200000	-0.8783160000	-2.1332040000
H	6.1431800000	-0.1732750000	-0.9399080000
H	6.0565720000	0.5051400000	-2.5588570000
H	6.0565330000	-0.5051240000	2.5589170000
H	5.0390080000	0.8783380000	2.1332200000
H	6.1432030000	0.1732790000	0.9399670000
H	5.4601630000	2.1353210000	0.1943310000
H	4.1783920000	3.0831730000	-0.5697940000
H	5.6848750000	2.7614930000	-1.4458240000

Sum of electronic and zero-point Energies = -2068.104725

Table S2. Cartesian coordinates of (*Z*)-**1**⊂OAc⁻.

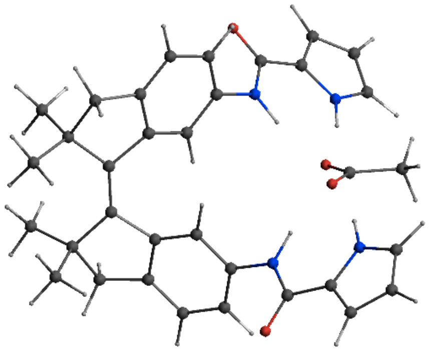

Atom	X	Y	Z
C	-3.2676970000	0.4331820000	0.5287410000
C	-3.2676290000	-0.4322340000	-0.5284880000
C	-2.0596280000	-1.1367730000	-1.0422960000
C	-2.2821850000	-1.5935600000	-2.3525760000
C	-3.6366390000	-1.1413360000	-2.8133720000
C	-4.4208970000	-0.8834910000	-1.4786740000
C	-2.0598300000	1.1380340000	1.0423310000
C	-2.2824810000	1.5954820000	2.3523730000
C	-3.6367740000	1.1430410000	2.8134360000
C	-4.4210320000	0.8844090000	1.4788830000
C	-0.9103180000	-1.5566670000	-0.3535010000
C	0.0164580000	-2.3861250000	-0.9942920000
C	-0.1890720000	-2.7882190000	-2.3244720000
C	-1.3476780000	-2.3999630000	-3.0012340000
C	-0.9105440000	1.5576170000	0.3533880000
C	0.0161860000	2.3874220000	0.9937900000
C	-0.1895160000	2.7904580000	2.3236510000
C	-1.3481650000	2.4024880000	3.0005440000
C	1.4760160000	4.1366500000	0.0648050000
O	0.7561580000	5.0603890000	0.4885510000
C	1.4740050000	-4.1369850000	-0.0660190000
O	0.7522910000	-5.0592840000	-0.4898100000
N	1.1743970000	2.8175100000	0.2787580000
N	1.1744400000	-2.8172990000	-0.2794130000
C	3.6575360000	0.0059390000	0.0136780000
N	3.5964440000	3.5246390000	-1.2070690000
C	4.5880110000	4.1779490000	-1.8760420000
C	4.3369440000	5.5446760000	-1.8193780000
C	3.1414570000	5.7128980000	-1.0809730000
C	2.6941400000	4.4459000000	-0.7066380000
N	3.5969190000	-3.5311950000	1.2057410000
C	4.5880710000	-4.1878400000	1.8720480000
C	4.3342590000	-5.5539050000	1.8125670000
C	3.1374830000	-5.7180810000	1.0753660000
C	2.6920250000	-4.4494460000	0.7043930000

C	-5.0418810000	-2.2296740000	-1.0305050000
C	-5.5238640000	-0.1464610000	1.7631150000
C	-5.5241480000	0.1470350000	-1.7625350000
C	-5.0425110000	2.2302530000	1.0303770000
O	3.0619880000	0.8611330000	-0.7121130000
O	3.0588950000	-0.8632410000	0.7180460000
C	5.1829630000	0.0133240000	0.0117570000
H	0.5445430000	-3.4243680000	-2.8071220000
H	0.5440410000	3.4269020000	2.8059870000
H	-4.1492780000	-1.8704590000	-3.4498220000
H	-3.5612080000	-0.2051090000	-3.3842750000
H	-4.1496110000	1.8722530000	3.4496220000
H	-3.5609880000	0.2070710000	3.3847150000
H	1.7535940000	2.0787550000	-0.1325270000
H	1.7543380000	-2.0788350000	0.1317750000
H	-0.7432360000	-1.2798080000	0.6819590000
H	-1.5232260000	-2.7427940000	-4.0175970000
H	-0.7433400000	1.2801500000	-0.6818860000
H	-1.5238080000	2.7459160000	4.0166900000
H	3.5460950000	2.5005510000	-1.0841290000
H	5.3960900000	3.6303570000	-2.3402900000
H	2.6437020000	6.6415290000	-0.8413470000
H	4.9465500000	6.3214210000	-2.2604180000
H	3.5483570000	-2.5072660000	1.0866230000
H	5.3978870000	-3.6427400000	2.3361800000
H	2.6377310000	-6.6451820000	0.8340110000
H	4.9428510000	-6.3328730000	2.2510770000
H	-4.2719890000	-3.0010770000	-0.9249780000
H	-5.7642890000	-2.5740530000	-1.7799620000
H	-5.5627530000	-2.1403560000	-0.0758900000
H	-5.0960250000	-1.1196250000	2.0242140000
H	-6.2116390000	-0.2852530000	0.9288840000
H	-6.1203570000	0.1962560000	2.6170500000
H	-6.1204960000	-0.1956460000	-2.6165850000
H	-5.0967170000	1.1204600000	-2.0233160000
H	-6.2119890000	0.2852420000	-0.9282640000
H	-5.5635520000	2.1404990000	0.0759070000
H	-4.2728660000	3.0018510000	0.9244600000
H	-5.7648660000	2.5746560000	1.7798730000
H	5.5643070000	1.0379700000	0.0263270000
H	5.5364200000	-0.4550530000	-0.9142950000
H	5.5872790000	-0.5448810000	0.8583610000

Sum of electronic and zero-point Energies = -1836.333204

References

(1) (a) Wezenberg, S. J.; Feringa, B. L. *Org. Lett.* **2017**, *19*, 324–327. (b) Wezenberg. S. J.; Chen. L.-J.; Bos. J. E.; Feringa. B. L.; Howe. E. N; Wu. X.; Siegler. M. A.; Gale. P. A. *J. Am. Chem. Soc*. **2022**, *144*, 331−338.

(2) Frassineti. C.; Ghelli. S.; Gans. P; Sabatini. A.; Moruzzi, M. S.; Vacca. A. *Anal. Biochem.* **1995**, *231*, 374−382.

(3) http://www.arguslab.com/arguslab.com/ArgusLab.html

(4) Gaussian 09, Revision D.01, Frisch, M. J.; Trucks, G. W.; Schlegel, H. B.; Scuseria, G. E.; Robb, M. A.; Cheeseman, J. R.; Scalmani, G.; Barone, V.; Mennucci, B.; Petersson, G. A.; Nakatsuji, H.; Caricato, M.; Li, X.; Hratchian, H. P.; Izmaylov, A. F.; Bloino, J.; Zheng, G.; Sonnenberg, J. L.; Hada, M.; Ehara, M.; Toyota, K.; Fukuda, R.; Hasegawa, J.; Ishida, M.; Nakajima, T.; Honda, Y.; Kitao, O.; Nakai, H.; Vreven, T.; Montgomery, Jr., J. A.; Peralta, J. E.; Ogliaro, F.; Bearpark, M.; Heyd, J. J.; Brothers, E.; Kudin, K. N.; Staroverov, V. N.; Keith, T.; Kobayashi, R.; Normand, J.; Raghavachari, K.; Rendell, A.; Burant, J. C.; Iyengar, S. S.; Tomasi, J.; Cossi, M.; Rega, N.; Millam, J. M.; Klene, M.; Knox, J. E.; Cross, J. B.; Bakken, V.; Adamo, C.; Jaramillo, J.; Gomperts, R.; Stratmann, R. E.; Yazyev, O.; Austin, A. J.; Cammi, R.; Pomelli, C.; Ochterski, J. W.; Martin, R. L.; Morokuma, K.; Zakrzewski, V. G.; Voth, G. A.; Salvador, P.; Dannenberg, J. J.; Dapprich, S.; Daniels, A. D.; Farkas, O.; Foresman, J. B.; Ortiz, J. V.; Cioslowski, J.; Fox, D. J.; Gaussian, Inc., Wallingford CT, **2013**.
